# Seasonal Patterns in Spectral Irradiance and Leaf UV-A Absorbance Under Forest Canopies

**DOI:** 10.3389/fpls.2019.01762

**Published:** 2020-02-18

**Authors:** Saara Maria Hartikainen, Marta Pieristè, Joose Lassila, Thomas Matthew Robson

**Affiliations:** ^1^ Canopy Spectral Ecology and Ecophysiology Group (CanSEE), Organismal and Evolutionary Biology (OEB), Viikki Plant Science Centre (ViPS), Faculty of Biological and Environmental Sciences, University of Helsinki, Helsinki, Finland; ^2^ Normandie Université, UNIROUEN, IRSTEA, ECODIV, FR Scale CNRS 3730, Rouen, France

**Keywords:** flavonoids, flavonol index, phenology, secondary metabolism, spectral irradiance, UV radiation

## Abstract

Plants commonly respond to UV radiation through the accumulation of flavonoids and related phenolic compounds which potentially ameliorate UV-damage to crucial internal structures. However, the seasonal dynamics of leaf flavonoids corresponding to epidermal UV absorbance is highly variable in nature, and it remains uncertain how environmental factors combine to govern flavonoid accumulation and degradation. We studied leaf UV-A absorbance of species composing the understorey plant community throughout two growing seasons under five adjacent tree canopies in southern Finland. We compared the relationship between leaf flavonol index (I_flav_—repeatedly measured with an optical leaf clip Dualex) and measured spectral irradiance, understorey and canopy phenology, air temperature and snowpack variables, whole leaf flavonoid extracts, and leaf age. Strong seasonal patterns and stand-related differences were apparent in I_flav_ of both understorey plant communities and individual species, including divergent trends in I_flav_ during spring and autumn. Comparing the heterogeneity of the understorey light environment and its spectral composition in looking for potential drivers of seasonal changes in I_flav_, we found that unweighted UV-A irradiance, or the effective UV dose calculated according to the biological spectral weighting function (BSWF) for plant growth (PG action spectrum), in understorey shade had a strong relationship with I_flav_. Furthermore, understorey species seemed to adjust I_flav_ to low background diffuse irradiance rather than infrequent high direct-beam irradiance in sunflecks during summer, since leaves produced during or after canopy closure had low I_flav_. In conclusion, we found the level of epidermal flavonoids in forest understorey species to be plastic, adjusting to climatic conditions, and differing according to species' leaf retention strategy and new leaf production, all of which contribute to the seasonal trends in leaf flavonoids found within forest stands.

## Introduction

Spectral irradiance changes in forest understoreys by season with solar elevation angle and with canopy leaf-out, as incident solar radiation is selectively absorbed in leaves (Grace, 1983; Endler, 1993). Furthermore, spectral regions penetrate differently through forest canopies and differ in their contribution to direct and diffuse radiation (Grant, 1997; Hertel et al., 2011; Dengel et al., 2015). Spring-time changes in deciduous forest canopies are well-known to affect the species in understorey plant communities, which adjust to changing light conditions to optimise their growth strategy and survival (Rothstein and Zak, 2001; Augspurger et al., 2017; Heberling et al., 2019). There is ample evidence that plants can perceive and respond to changes in the spectral composition of received irradiance in forest understoreys (Smith, 1982; Valladares, 2003). However, most research has focussed on the response of understorey plants to photosynthetically active radiation (PAR, 400–700 nm), while ultraviolet radiation (UV-B 280–315 nm, and UV-A 315–400 nm) in forests has received less attention (Grant, 1997; Flint and Caldwell, 1998; Grant et al., 2005). UV-B-induced responses in plants often depend on the interplay between different spectral regions, namely PAR, UV-B, and UV-A radiation, involved in high-light acclimation and repair processes (Caldwell et al., 1994; Jansen et al., 1998; Verdaguer et al., 2017). Historically, UV-B radiation was perceived as a source of stress and research focused on experiments exposing plants to high doses of UV-B radiation, or studying plants in environments with high UV-B radiation (Rozema et al., 1997; Searles et al., 2001; Björn, 2015). However, our current understanding of UV-A and UV-B radiation as triggers of regulatory responses has shifted emphasis towards studying the effects of realistic and low UV doses on plants (e.g. Kolb et al., 2001; Hectors et al., 2007; Brelsford et al., 2019). These realistic UV radiation conditions often act as eustress, stimulating responses and possible cross-tolerance through mechanisms that are not yet well elucidated (Hideg et al., 2013).

Plants are known to produce flavonoids and related phenolic compounds in response to UV-B radiation (Searles et al., 2001). Flavonoids are considered to have a diversity of functions, including the potential to act as antioxidants in mesophyll cells and reducing harmful effects of those reactive oxygen species (ROS) produced under stress (Hernández et al., 2009; Agati and Tattini, 2010). Furthermore, these compounds may act as a selective filter in the leaf epidermis, attenuating most of the incident UV radiation, preventing cellular damage within (Day et al., 1993; Ålenius et al., 1995; Cockell and Knowland, 1999). This feature is common among plant taxa (Robberecht et al., 1980; Day et al., 1992; Qi et al., 2010), but although high leaf flavonoid content is typical of plants growing in high UV radiation environments (Ziska et al., 1992; Rozema et al., 1997), many studies have found no-more-than a weak relationship between UV radiation and UV-screening or associated flavonoids both in nature and under controlled conditions (Liakoura et al., 2001; Nybakken et al., 2004b; Barnes et al., 2016b). Furthermore, the accumulation of UV-screening compounds can be induced in absence of UV radiation by low temperature (Bilger et al., 2007) and by PAR in developing leaves (Barnes et al., 2013). Their multiplicity of roles complicates our interpretation of the relationship between flavonoid induction and their function in complex natural environments. Since flavonoids are a diverse metabolite group (Harborne and Williams, 2000), many qualitative and quantitative differences have been found, e.g. seasonally (Liakoura et al., 2001; Kotilainen et al., 2010), with leaf longevity (Semerdjieva et al., 2003), with leaf development (Laitinen et al., 2002), and within same species grown under differing environments (Comont et al., 2012; Castagna et al., 2017).

Most studies have found UV-screening in plant species to adjust to different environments (Krause et al., 2003; Nybakken et al., 2004a) or to be flexible in short-term (i.e. diurnal changes) (Barnes et al., 2008; Barnes et al., 2016a), but very few experiments have tested the extent of plasticity within species. However, where tested, a few species or populations have been found to attain high constitutive UV absorbance varying little with the environment (Ziska et al., 1992; Nybakken et al., 2004a). Generally, we still lack knowledge of the mechanisms underpinning variation in leaf UV absorbance among plant species, and interactions with different environmental factors that produce such variation in leaf epidermal flavonoids and related phenolic compounds. Traditionally, UV-screening studies had to rely on invasive techniques (Day et al., 1992; Aphalo et al., 2012), but recent developments in optics-based methods are now enabling repeated *in vivo* sampling by for instance Dualex Scientific^+^ (Cerovic et al., 2012; FORCE-A, Paris-Orsay, FR) which provides insight into long-term seasonal trends in epidermal UV absorbance. The resulting index representing leaf epidermal UV-A absorbance measured with Dualex Scientific^+^ is ostensibly controlled by flavonoids, with potential contribution from hydroxycinnamic acid derivatives (HCAs) (Cerovic et al., 2005; Agati et al., 2008).

Many questions on the ecological role of UV responses remain unanswered (Barnes, 2016c; Robson et al., 2019), including how plant secondary metabolite responses controlling UV absorbance may be modified by climatic and environmental factors. This consideration is particularly relevant for forest understorey species whose growth may be limited by the light environment (Valladares, 2003; Heberling et al., 2019). As canopy tree species is known to affect spectral irradiance in the understorey (Canham et al., 1994; Hertel et al., 2011), we aimed to account for this variation by including different forest stands with contrasting evergreen and deciduous canopies, of different ages in our study. To better understand the ecology of understorey plant UV responses we investigated 1) how leaf epidermal flavonoids change seasonally within and among different understorey species; 2) whether changes in understorey plants' epidermal flavonoids relate to changes in spectral irradiance during spring and summer in different forest stands; and 3) how other factors that are known to sometimes interact with leaf flavonoid accumulation (e.g. the timing of leaf and plant phenology; **Table S1**), affect seasonal trends in UV-A absorbance. We focused on seasonal changes within the UV region measured from forest understoreys i.e. the unweighted or effective doses of UV radiation calculated according to different biological spectral weighting functions (BSWF). To answer these questions, we collected spectral irradiance data ranging from UV radiation to near infrared radiation (280–900 nm), and optically measured leaf epidermal UV-A absorbance i.e. I_flav_ from 35 understorey species over spring and summer.

## Materials and Methods

### Experimental Design and Description of the Site

Forest stands at Lammi Biological Station (LBS) (N 61°3'14.6”, E 25°2'13.8”) represent typical managed forests in Finland, but have been left to grow naturally through the latter 20^th^ century. Five forest stands were chosen based on the canopy species, age, and stand structure; three different aged deciduous *Betula* sp. L. -dominated stands (henceforth: *Betula* old, young, and mixed with other canopy species), one deciduous *Quercus robur* L. stand, and one evergreen *Picea abies* (L.) H. Karst. stand. Detailed stand characteristics are given in **Table S2**, and the Plant Area Index (PAI) calculated from hemispherical photographs during spring and summer 2015 are given in **Figure 5** and **Supplementary A3**. The *Q. robur* stand was planted in the 1950's, but was used in our study due to our particular interest in canopies of different architecture and phenology. All stands have understorey vegetation reflecting their edaphic environment and are typical of forests with these canopy species in Finland. Four “measurement points” approximately equidistance between the nearest trees were established in each stand. We considered this the minimum number of replicate patches required to describe small scale variation in irradiance (Hartikainen et al., 2018) and in the plant community in each stand, while remaining feasible to measure close to solar noon.

Over spring and summer of 2015, four to five repeated sets of irradiance measurements and corresponding optical leaf trait measurements were made at each measurement point. Over the growing season of 2016, optical leaf trait measurements focussed on six common understorey species in the same stands, beyond a 3-m radius from the measurement points. Daily temperature (mean, max, min) and snowpack evolution data were recorded by the LBS weather station managed by the Finnish Meteorological Institute situated < 600 m from the forest stands (**Table S3**). The seasons were defined as periods with the mean daily air temperature continuously above 0°C (spring), above +10°C (summer), below +10°C (autumn), or below 0°C degrees (winter). The respective dates for the two sampling years are given in **Table S3**.

### Repeated Optical Leaf Trait Measurements From Understorey Plants

The optical leaf measurements were made on all individual plants (or possibly ramets) of understorey species growing within a 3-m radius of the measurement points: ≥ four individuals per species per measuring point where present. Absorbance by flavonoids (flavonols in dicots and flavones in monocots), anthocyanins, and leaf chlorophyll content, was measured from the leaf adaxial side non-invasively with optical leaf clip Dualex Scientific ^+^ (henceforth Dualex) during 2015. In 2016 measurements were made from both leaf sides (adaxial and abaxial). Based on the relative change in chlorophyll fluorescence, the Dualex obtains an index of UV-A absorbance at 375 nm which lies within the tail of the flavonoid spectral absorption peak (Cerovic et al., 2012). The three absorbance indices were considered to be approximately linear compared to the respective content within a leaf over the range of values obtained (unpublished data). The measurements were done around solar noon (approximately ± 3 hours) to exclude potential major diurnal variation in UV absorbance by flavonols and chloroplast movement (Williams et al., 2003; Barnes et al., 2016a). Measurements were made on the first distal mature leaf of the main stem, usually the 3^rd^ or 4^th^ leaf from the top which was not shaded by other leaves. Further measurements were made to compare this standard against younger and older leaves, to record and account for changes related to leaf age. These measurements were made in species with overwintering leaves: *Fragaria vesca* L., *Hepatica nobilis* Schreb., *Oxalis acetosella* L., *Vaccinium vitis-idaea* L., and in summer green species: *Campanula persicifolia* L. and *Convallaria majalis* L. Only visibly healthy leaves were measured to avoid confounding results due to herbivory or other damage. Understorey species abundances were recorded within a 3-m radius of the measurement points, and species' phenology (i.e. timing of emergence, leaf opening, flowering, seed production, and senescence) was recorded at the stand level. Community weighted means (CWM) for flavonol index (I_flav_) were calculated for each measurement point on each DOY, by multiplying Dualex values by relative abundances of each species measured.

### Comparison of Optically Measured I_flav_ and Extracted Flavonoids

During the spring and summer of 2016, leaf samples of understorey species were collected from two contrasting forest stands, those with a *Q. robur* and *P. abies* canopies, to test the relationship between the flavonoid content measured in intact leaves using a Dualex and from leaf extracts using a spectrophotometer. Spectrophotometric readings of extracted flavonoids were taken from same leaves measured with the Dualex in three prevalent understorey species: *H. nobilis* and *O. acetosella* from the *P. abies* stand; *Aegopodium podagraria* L. and *C. majalis* from the *Q. robur* stand, and *Anemone nemorosa* L. from both stands. Fifteen individuals per species per stand were sampled on five to six occasions. Dualex measurements were made in the middle section of the lamina of sampled leaves avoiding major veins, prior to leaf-disk sampling.

Two leaf-disks from each leaf were punched (2 × 0.28 cm^2^ area) *in situ* directly into 3 ml of acidified methanol (99,9% MeOH acidified with HCl 1:200). All samples were kept dark and on ice in a cold box in the field during sampling and subsequently at +6°C overnight. Extracts were analysed with a spectrophotometer (Shimadzu UV-2501 PC UV-VIS, Kyoto, Japan) recording absorption spectra from 190 to 900 nm using a quartz cuvette and samples were diluted if necessary to keep absorbance values ≤ 2. To test the relationship with Dualex measurements four values were compared: absorbance at 375 nm, mean absorbance within the UV-B spectrum, UV-A spectrum, and the whole UV (UV-B plus UV-A) spectrum.

Additional equivalent leaf-disks were taken to obtain fresh and dry weights from the same leaves. These leaf-disks were kept in sealed plastic bags in the cold and dark until weighing for fresh weight shortly after sampling. Leaf-disk dry weights were measured after >24 hours drying at +50°C. The absorbance of leaf extracts was normalised for sample volume and leaf fresh weight.

### Irradiance Measurements Below Forest Canopies

Solar spectral irradiance under the forest canopies was measured with portable CCD array spectroradiometer Maya 2000 pro (Ocean Optics, Dunedin, FL, USA) with D7-H-SMA cosine diffuser (Bentham Instruments Ltd., Reading, UK) with spectral range of 200–1100 nm. The spectrometer was calibrated by Finnish Radiation and Nuclear Safety Authority (Ylianttila et al., 2005; Aphalo et al., 2016; Aphalo, 2017) for accurate outdoor solar radiation measurement from UV-B to near-infrared radiation. The detailed measurement and post-processing protocol used is described in Hartikainen et al. (2018). The final replicate of irradiance readings from *P. abies* stand and three measurement points from *Betula* young stand are missing, since the fibre-optic cable to the diffuser broke.

All irradiance measurements were made within 3 hours of peak solar irradiance at solar noon, under weather conditions that were as close to clear sky as occurred at the field site each month (**Figure S1**). To achieve measurements which encompassed the range of variation in under-canopy irradiance, three sets of measurements were made at each measurement point: 1) in a *sunfleck* consisting mostly of direct radiation (Smith and Berry, 2013), 2) within an umbra (*shade*) of a tree trunk consisting of diffuse radiation, and 3) at a point where radiation penetrated through a canopy of leaves (henceforth understorey *leaf* position) (Hartikainen et al., 2018).

### Data Analyses

The stand-related differences in I_flav_ trends were compared by inspecting any overlap between 95% confidence intervals (CI) (Di Stefano, 2004; Martínez-Abraín, 2007) of loess-based fits (obtained with R function “loess”) based on values from individual plants and averages per measurement point. The same approach was used to compare year-to-year consistency of I_flav_ trends, species-specific patterns, and differences in trends in spectral irradiance. Additionally, differences in I_flav_, or in CWMs of I_flav_, between stands were tested using ANOVA for each DOY. Likewise, ANOVAs were used to test for differences in I_flav_ between different-aged leaves and species-specific patterns in I_flav_ between stands. A two-sample Student's *t*-test or non-parametric Wilcoxon test was used to test for differences between I_flav_ of the adaxial and abaxial leaf sides. The same approach was used to test for differences between I_flav_ of new spring leaves of *H. nobilis* at their initial emergence and during summer, and between I_flav_ of overwintered leaves at first and last measurement.

Weather station data were compared with I_flav_ from 2015 and 2016 to test whether temperature was an important driver of the observed I_flav_ trend. To assess any differences between the two consecutive years with respect to the spring onset of the growing season, the following weather variables were calculated: *days post snowmelt* in spring, *days prior to first marked snowfall* in winter, *days from beginning of thermal growing season*, and *the effective temperature sum* for a given DOY. The relationship of these variables with mean I_flav_ was investigated through Pearson's correlation. The relationship between leaf adaxial or abaxial I_flav_ and minimum daily air temperature was investigated through generalised additive mixed models (GAMMs, R package NLME, Pinheiro et al., 2019). As all weather-related variables were co-linear (**Figure S2**), we chose minimum air temperature as the explanatory variable in the model based on its high negative correlation with I_flav_ and the expected response of I_flav_ to low temperatures. The relationship between different methods of quantifying leaf flavonoids i.e. Dualex measurements vs. leaf extracts, was investigated with Pearson's correlation and with GAMM. Appropriate variance structures were assigned to the species-specific models, allowing for residual spread to change for different DOY (R function varIdent), over different values of the explanatory variable (R function varFixed), or for both (R function varComb), and a temporal correlation structure (compound symmetry auto-correlation structure, R function corCompSymm). Equivalent analyses to these were used to test the relationship between mean I_flav_ and understorey spectral irradiance (unweighted and BSWFs) in different understorey positions (sunfleck, shade, *leaf*). Different variance structures e.g. allowing for residual spread to change for different stands (R function varIdent) and a temporal correlation structure (R function corCompSymm) were tested. All data exploration and analysis were performed in R version 3.5.2 (2018, The R Foundation for Statistical Computing, Vienna, Austria). All figures were created with R package ggplot2 (Wickham, 2016).

## Results

### Repeated Optical Leaf Trait Measurements From Understorey Plants

#### Seasonal Trends in I_flav_ From Plants in Forest Stands Across Consecutive Years

**Figure 1:**
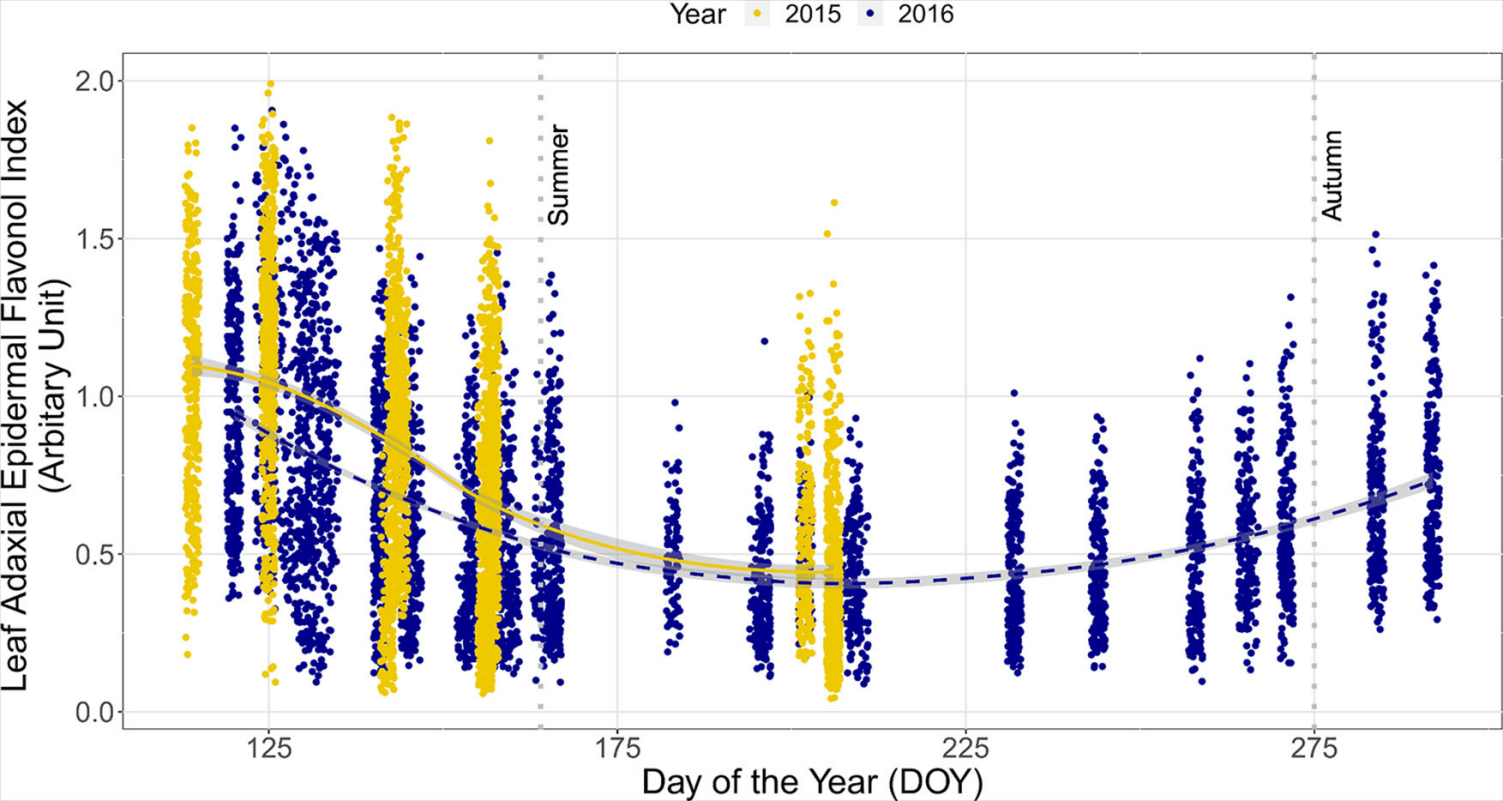
Trends in the leaf adaxial epidermal flavonol index (I_flav_) from understorey species growing in total of five forest stands in two consecutive years. The trend lines are given by a loess fit to the cloud of points for each year with 95% CI (grey band). Data from 2015 include all 35 understorey species at specific measurement points, whereas data from 2016 comprise of six species growing across the whole of each stand. The vertical grey dotted lines indicate the approximate beginning of summer and autumn with respective mean daily air temperatures continuously above +10°C degrees and subsequently below +10°C. Restricting the data fitted by the smoother to the same time period and same species in both years did not affect the result. Further details of weather during the sampling years are provided in **Table S3**.

**Figure 2:**
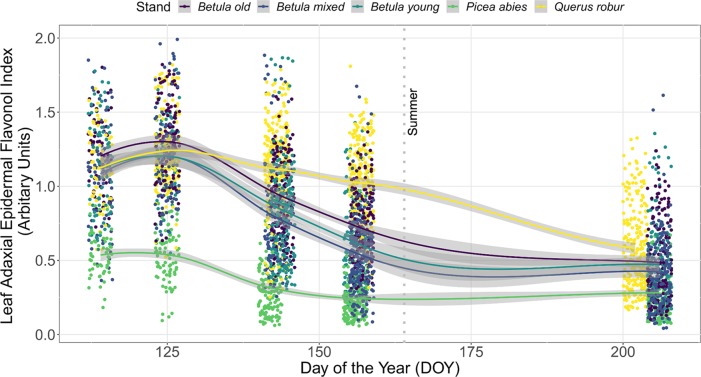
Leaf adaxial epidermal flavonol index (I_flav_) from understorey species growing in five forest stands measured during spring and summer in 2015. A total of 35 understorey species were present in different stands. The trend lines are given by a loess fit to the cloud of points for each stand with 95% CI (grey band). Each point represents a measurement from an individual plant. The vertical grey dotted line indicates the approximate beginning of summer with respective mean daily air temperatures continuously above +10°C degrees. Further details of weather during 2015 are provided in **Table S3**.

The seasonal trend in I_flav_ with DOY was similar between consecutive years, although I_flav_ was slightly higher in the spring of 2015 than 2016 (non-overlapping CIs, **Figure 1**). This trend differed among stands whereby understorey I_flav_ was clearly lowest in the *P. abies* stand, whereas trends in I_flav_ were indistinguishable among the three *Betula* stands with considerable 95% CI overlap throughout spring and summer (**Figure 2**, **Table S4**). These differences among stands held whether comparing individual plants (**Figure 2**) or averages across the measurement points (**Figure S3**), but were slightly less distinct in 2016 than 2015 (**Figure S4**). The 2015 trend in understorey I_flav_ from the *Q. robur* stand differed from all the other stands, since I_flav_ declined there most gradually (from DOY 142–144 onwards) from its initial spring peak (**Figure 2**). However, based on CI-overlap this difference was only evident on a per plant basis in 2015, not from the measurement point averages (**Figure S3**), or per plant basis in 2016 (**Figure S4**). The understorey I_flav_ from each stand followed a similar time course: initially high values on DOY 114 or 125 with a subsequent decline to a minimum by DOY 202/206 in the deciduous stands, and by DOY 156/157 in the *P. abies* stand (**Figure 2**, **Table S4**).

In addition to the seasonal trends, significant differences among stands in understorey I_flav_ were identified on each measurement date (**Table S4**). When only deciduous stands were compared, these differences were less evident, but still significant from DOY 125 onward (**Table S4**). Measurements of six species extending late into the 2016 growing season to DOY 292, revealed that the I_flav_ of two species with overwintering leaves had no significant differences among stands by mid-July (DOY 195–202) when I_flav_ reached its lowest values in the deciduous stands, while in the autumn divergent trends in I_flav_ among stands resembled those occurring in spring (**Table S5**).

The seasonal trend in I_flav_ remained similar irrespective of whether the composition of the understorey plant community was accounted for using simple averages, or as a CWMs (**Figure S5** and **Table S4**). However, stand-related differences were less distinct as CWMs; whereby all stands were indistinguishable at the beginning (DOY 114) and at the end (DOY 202/206) of the 2015 measurement period (**Table S4**). Furthermore, the difference between plants growing in the *Q. robur* and *Betula* stands late in the spring was no longer as evident when CWMs for I_flav_ were used (**Figure S5**: overlap in 95% CIs, **Table S4**).

#### Seasonal Differences Between Adaxial and Abaxial I_flav_

The relationship between I_flav_ measured from the leaf adaxial and abaxial sides varied seasonally in a species-specific manner (**Figure S6**). In *A. nemorosa* and *C. majalis* I_flav_ on either leaf side differed throughout the growing season, whereas in *A. podagraria* this difference was only present during spring (**Figure S6**). Furthermore, I_flav_ differed between leaf sides both in spring and autumn in species with overwintering leaves, *F. vesca* and *H. nobilis*, although not in *O. acetosella* which provided the exception (**Figure S6**). Among those species where I_flav_ differed according to leaf side, values for the adaxial side typically increased in spring, autumn, or both, and were higher than those of the abaxial side with a few exceptions at low I_flav_ values in *F. vesca* (**Figure S6**).

#### Effect of the Weather in Consecutive Years on Seasonal Changes in I_flav_

Over 2015 I_flav_ correlated negatively (*r* = −0.66) with variables describing progression through the growing season (days prior to winter snowfall, days post spring snowmelt, days from the beginning of thermal growing season), while its relationship with minimum daily air temperature was weaker (r = −0.59) (**Table S6**). On the contrary, over 2016 I_flav_ correlated best with minimum daily air temperature (*r =* −0.41) (**Table S6**). In comparison to 2015, the relationships between I_flav_ and other weather-related variables were weaker in 2016 (**Table S6**). The model combining both years with minimum air temperature as explanatory variable only poorly explained changes in I_flav_ on both leaf sides (R^2^ -values: 0.47; 0.33 for models with adaxial and abaxial I_flav_ respectively) (**Supplementary A2** and **Figure S2**).

### Understorey Species-Specific Patterns in I_flav_

**Figure 3:**
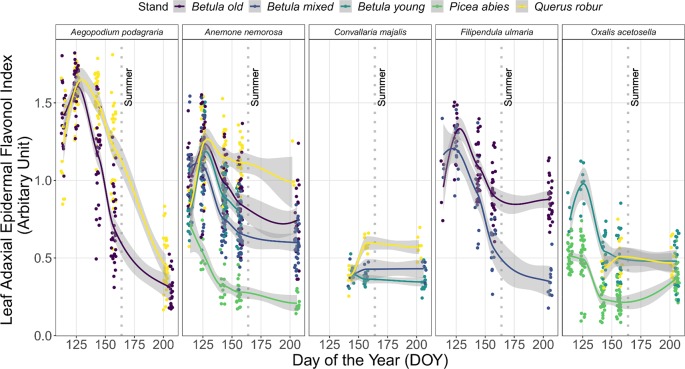
Trends in leaf epidermal flavonol index (I_flav_) from five understorey species (*A. podagraria*, *A. nemorosa*, *C. majalis*, *F. ulmaria*, and *O. acetosella*) measured on five occasions during spring and summer 2015. The trend lines are given by loess fits to the cloud of points for each stand with 95% CI (grey band). The vertical grey dotted lines indicate the approximate beginning of summer with respective mean daily air temperatures continuously above +10°C degrees. Further details of weather during 2015 are provided in **Table S3**.

In both years, I_flav_ differed significantly among species within each stand on each DOY (*p* < 0.001 in each case) with only two exceptions (in the *Betula* old stand in 2016: on DOY 131 *p* = 0.07, on DOY 141 *p =* 0.045). The trends in I_flav_ for five abundant understorey species in 2015 are highlighted in **Figure 3**. Of these species, *A. podagraria* and *Filipendula ulmaria* (L.) Maxim. followed similar patterns to the general trend described above. Although there were stand-specific differences in I_flav_ in these two species, this seasonal pattern was consistent across all stands where they were measured (**Figure 3** and **Table S7**). There were also stand-related differences in I_flav_ trends in *A. nemorosa,* which had lowest I_flav_ values in the *P. abies* stand, and distinct trend in the *Q. robur* stand compared with the *Betula* stands from DOY 142–144 onward (**Figure 3**: no overlap in 95% CIs, **Table S7**). *C. majalis* was the last of the five understorey species to emerge in spring, and its I_flav_ attained modest values compared to other species (**Figure 3** and **Table S7**). New leaves of *O. acetosella* were produced later (around DOY 125) in the *Q. robur* stand than in the other stands, with only a minor increase in I_flav_ throughout summer (**Figure 3** and **Table S7**), thus creating a very different time-course pattern compared to the same species growing elsewhere. Species-specific patterns mostly held in the following year, although more frequent measurements revealed some additional differences during autumn in species with overwintering leaves (**Figure S7**).

### Relating Leaf Retention Strategy and Leaf Age to I_flav_

The I_flav_ differed between concurrently measured leaves of different ages (**Table S8**). This was based on the comparison of two summer green species (**Table S8**). Furthermore, differences in the phenology of the four species with overwintering leaves affected species-specific patterns in I_flav_ related to the timing of leaf production, because new leaves produced after the start of the growing season had significantly lower I_flav_ values than mature leaves measured on the same dates (**Table S8** and **Figure S8**). A lower I_flav_ in new leaves was found both among species producing a distinct cohort of new leaves once during the growing season (i.e. *H. nobilis*), and those species producing new leaves throughout the growing season (i.e. *F. vesca*) (**Table S8**). Similarly, when I_flav_ of different aged leaves of *H. nobilis* was compared during 2016 in four stands, old and new spring leaves were significantly different (*p* < 0.01 each time) throughout spring until the senescence of old leaves (DOY 133–166), excluding initial emergence of new leaves in the two stands where no difference was found (data not shown). Further tests of different aged leaves from this species revealed that I_flav_ changes within young spring leaves were largely responsible for the species' average seasonal decline (**Table S9**). On the contrary, the I_flav_ of the overwintered leaves did not differ during spring in two stands, and only in overwintering leaves from *Q. robur* stand I_flav_ declined during spring (**Table S9**).

### Comparison of Optically Measured I_flav_ and Extracted Flavonoids

The I_flav_ measured at 375 nm by Dualex, had a stronger relationship with the absorbance of leaf-disk extracts when mean absorbance over the spectral regions of UV-B, UV-A or the entire UV spectrum was used than it did with absorbance of extracts at 375 nm (**Table S10**). There were species-specific differences in the strength of this relationship which was stronger in general for *A. nemorosa* and *H. nobilis* than for *C. majalis* (**Table S10**). The best selected species-specific GAMMs or GLS for *A. podagraria*, *C. majalis*, and *O. acetosella* used mean absorbance of extracts over the UV-B region as the explanatory variable, while for *A. nemorosa* and *H. nobilis* mean absorbance of extracts over UV-A region gave the best fits (all details in **Supplementary A2**). However, the highest peak in absorbance in the UV spectrum was at ~330 nm throughout the spring and summer season for *A. podagraria, A. nemorosa,* and *H. nobilis*, but this peak was not as distinctive in *O. acetosella* and *C. majalis* (**Figure S9**).

There were seasonal differences in the relationship between I_flav_ and flavonoid extracts over spring and summer (**Supplementary A2**, **Figure S9**). The modest initial early-season peak on DOY 125 in I_flav_, found in all species but *C. majalis* on that date, was only visible in extracts of *H. nobilis* and *A. nemorosa* from the *P. abies* stand (**Table S10**, **Supplementary A2**). Despite these inconsistencies, both methods revealed differences in flavonoids between *A. nemorosa* growing in those stands with contrasting light environments (**Table S11**); which we used to verify the reliability of these approaches.

### Irradiance Measurements Below Forest Canopies

**Figure 4:**
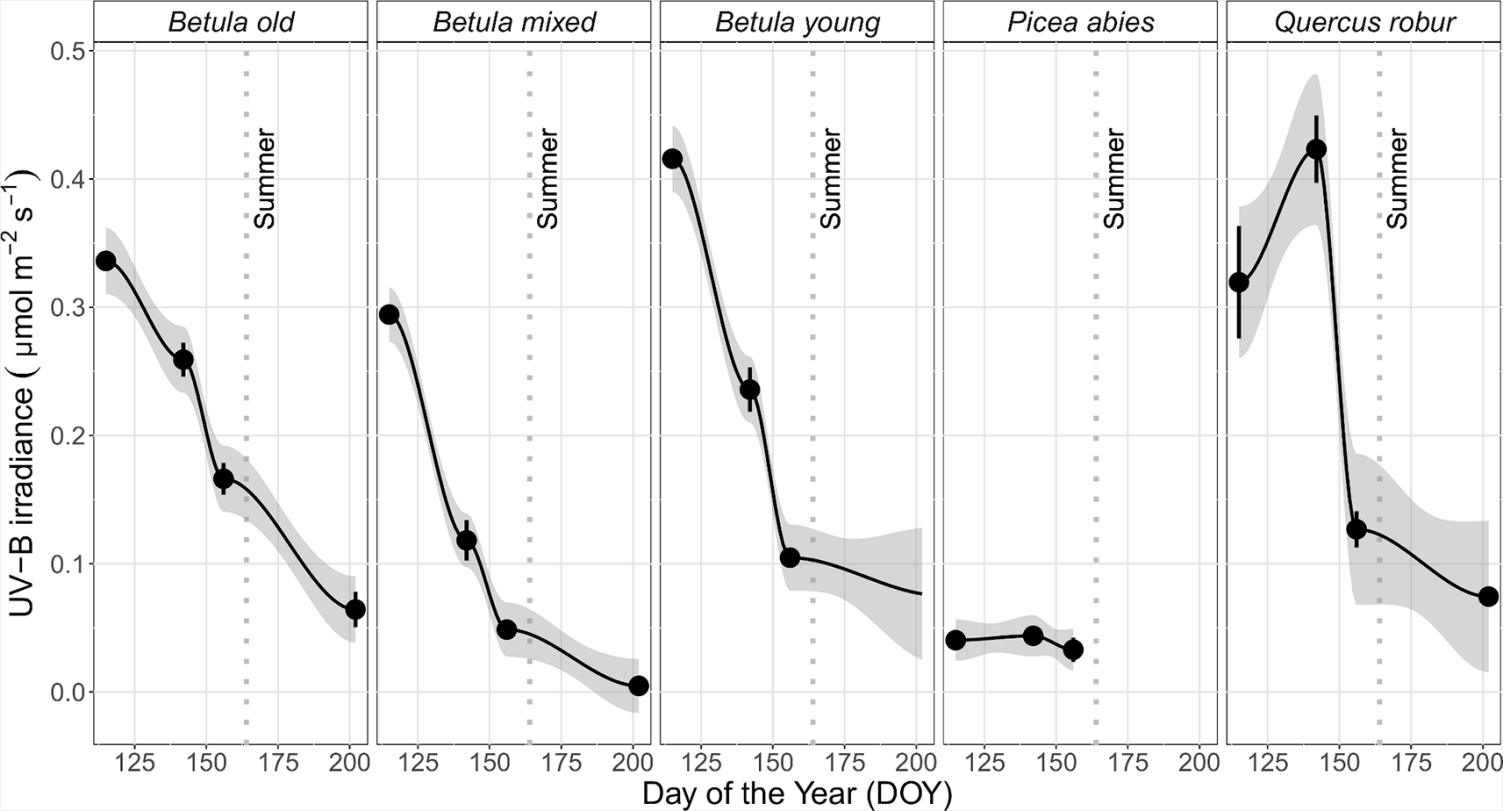
Mean (± SE) of unweighted UV-B irradiance (µmol m^−2^ s^−1^) in understorey shade by DOY in five studied stands differing in canopy composition (deciduous *Betula* old, mixed and young; evergreen *Picea abies*; deciduous *Quercus robur*) measured during spring and summer, 2015. Measurements were made with a calibrated array spectroradiometer connected *via* a fibre-optic cable to a levelled cosine diffuser. The trend lines are given by a loess fit for each stand with 95% CI (grey band). The vertical grey dotted lines indicate the approximate beginning of summer with respective mean daily air temperatures continuously above +10°C degrees. Further details of weather during 2015 are provided in **Table S3**.

**Figure 5:**
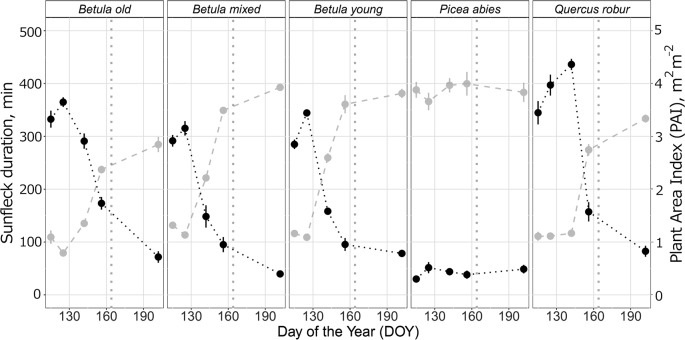
Mean (± SE) sunfleck duration in minutes for each measurement day (black) and corresponding Plant Area Index, m^2^ m^−2^ (light grey) in five different stands (deciduous *Betula* old, mixed and young; evergreen *Picea abies*; deciduous *Quercus robur*) during spring and summer 2015. The data were acquired from hemispherical photographs and all details of the calculation protocol are provided in **Supplementary A3**. The vertical grey dotted lines indicate the approximate beginning of summer with respective mean daily air temperatures continuously above +10°C degrees.

In general, the irradiance in understorey sunflecks was more variable across the measured spectrum than irradiance in understorey shade or in the *leaf* position (**Table 1**, **Figure S10**, **Table S12**). Our previous analysis (Hartikainen et al., 2018) found that the shape of the spectra persisted in sunflecks among stands and through the spring and summer season, which suggests this variation stemmed from differences in the size of the sunflecks. The time-course changes within each spectral region differed among the stands, especially in understorey shade, and in particular trends from the *P. abies* stand differed from all the other stands both in shade and in sunflecks (**Figure 4** and **Figure S10**: non-overlapping CIs in early spring). Time-course changes in irradiance in the *P. abies* stand mainly reflected its noticeably higher plant area index (PAI) early in the growing season in comparison to other stands, and seasonal differences in solar elevation angle (**Figures 4** and **5** and **Figure S10**).

**Table 1:** Mean (± SE) PAR, UV-B, and UV-A photon irradiance (µmol m^−2^ s^−1^) and effective UV dose (µmol m^−2^ s^−1^) calculated according to biological spectral weighting function for plant growth (PG action spectrum, ) measured in understorey sunflecks, shade and *leaf* positions, where *leaf* position refers to radiation that is transmitted through the canopy of leaves. Open reference measurements were taken in an open field area well outside the forest.

Stand	DOY	PAR (PPFD) ± SE			UV-B ± SE			UV-A ± SE			PG ± SE		
		Sunfleck	*Leaf* position	Shade	Sunfleck	*Leaf* position	Shade	Sunfleck	*Leaf* position	Shade	Sunfleck	*Leaf* position	Shade
*Betula* old	115	882.01 ± 131.97		144.05 ± 8.16	0.60 ± 0.04		0.336 ± 0.005	61.33 ± 6.83		22.47 ± 0.19	1.071 ± 0.110		0.430 ± 0.003
*Betula* old	142/144	729.89 ± 205.98	391.82 ± 42.71	85.15 ± 4.75	0.58 ± 0.10	0.43 ± 0.03	0.259 ± 0.013	50.04 ± 11.32	31.41 ± 2.41	14.03 ± 0.46	0.882 ± 0.190	0.577 ± 0.041	0.279 ± 0.010
*Betula* old	156	1269.03 ± 88.25	627.18 ± 209.19	49.07 ± 4.58	0.89 ± 0.23	0.59 ± 0.16	0.166 ± 0.012	80.87 ± 6.22	43.36 ± 13.01	7.79 ± 0.48	1.418 ± 0.132	0.778 ± 0.229	0.157 ± 0.010
*Betula* old	202	286.61 ± 64.48	149.75 ± 37.14	17.30 ± 2.26	0.23 ± 0.05	0.19 ± 0.03	0.064 ± 0.014	19.09 ± 3.60	11.61 ± 2.13	3.10 ± 0.54	0.338 ± 0.062	0.219 ± 0.038	0.062 ± 0.011
*Betula* mixed	115	519.80 ± 91.63		161.81 ± 10.68	0.44 ± 0.06		0.294 ± 0.007	40.58 ± 5.13		20.94 ± 0.43	0.723 ± 0.089		0.395 ± 0.006
*Betula* mixed	142/144	380.77 ± 102.47	97.63 ± 18.63	54.46 ± 1.63	0.27 ± 0.04	0.13 ± 0.02	0.118 ± 0.016	25.40 ± 5.09	10.08 ± 1.51	7.86 ± 0.61	0.445 ± 0.083	0.187 ± 0.027	0.151 ± 0.013
*Betula* mixed	156	570.20 ± 225.01	170.23 ± 22.51	32.31 ± 7.94	0.44 ± 0.17	0.15 ± 0.01	0.049 ± 0.007	35.51 ± 13.60	11.89 ± 1.10	3.25 ± 0.53	0.627 ± 0.242	0.210 ± 0.018	0.061 ± 0.009
*Betula* mixed	202	113.80 ± 24.17	17.19 ± 9.65	4.70 ± 0.37	0.06 ± 0.01	0.01 ± 0.01	0.005 ± 0.003	6.46 ± 1.27	1.23 ± 0.59	0.65 ± 0.12	0.109 ± 0.022	0.021 ± 0.010	0.012 ± 0.002
*Betula* young	115	963.39 ± 114.98		206.41 ± 22.73	0.78 ± 0.05		0.416 ± 0.008	68.98 ± 6.35		26.10 ± 1.07	1.222 ± 0.105		0.500 ± 0.016
*Betula* young	142/144	1043.08 ± 71.90	557.49 ± 105.01	111.83 ± 2.29	0.88 ± 0.04	0.55 ± 0.09	0.236 ± 0.017	68.66 ± 4.25	40.17 ± 6.41	13.55 ± 0.22	1.225 ± 0.070	0.728 ± 0.115	0.262 ± 0.006
*Betula* young	156	746.76 ± 98.06	199.24 ± 65.62	40.21 ± 3.03	0.47 ± 0.04	0.20 ± 0.03	0.105 ± 0.004	46.37 ± 5.32	15.07 ± 3.52	6.00 ± 0.14	0.795 ± 0.088	0.274 ± 0.058	0.118 ± 0.002
*Betula* young	202	814.06	140.86	38.74	0.43	0.03	0.076	49.53	9.33	5.01	0.835 ± 0.000	0.156 ± 0.000	0.093
*Picea abies*	115	86.42 ± 26.51		20.36 ± 3.11	0.06 ± 0.01		0.040 ± 0.006	6.53 ± 1.58		2.86 ± 0.34	0.114 ± 0.026		0.054 ± 0.007
*Picea abies*	142/144	521.44 ± 75.47	324.60 ± 63.03	29.90 ± 3.77	0.34 ± 0.04	0.16 ± 0.05	0.044 ± 0.005	32.76 ± 4.55	20.84 ± 3.47	3.43 ± 0.19	0.565 ± 0.077	0.353 ± 0.058	0.063 ± 0.004
*Picea abies*	156	871.34 ± 128.67	112.52 ± 56.49	17.61 ± 7.66	0.53 ± 0.07	0.07 ± 0.01	0.033 ± 0.009	52.84 ± 7.20	8.25 ± 3.36	2.68 ± 0.30	0.927 ± 0.113	0.142 ± 0.054	0.050 ± 0.004
*Picea abies*	202												
*Quercus robur*	115	1095.77 ± 107.79		190.61 ± 21.70	0.72 ± 0.11		0.319 ± 0.044	74.85 ± 6.41		24.79 ± 1.35	1.301 ± 0.107		0.464 ± 0.026
*Quercus robur*	142/144	1049.05 ± 134.73		93.13 ± 4.15	0.99 ± 0.11		0.423 ± 0.026	76.37 ± 8.41		19.69 ± 1.14	1.371 ± 0.150		0.403 ± 0.024
*Quercus robur*	156	756.19 ± 114.37	304.50 ± 117.60	51.45 ± 2.99	0.46 ± 0.05	0.26 ± 0.07	0.127 ± 0.014	46.81 ± 6.20	21.78 ± 7.15	7.32 ± 0.63	0.806 ± 0.104	0.389 ± 0.121	0.144 ± 0.013
*Quercus robur*	202	561.53 ± 31.00	207.29 ± 58.54	19.69 ± 0.46	0.47 ± 0.04	0.19 ± 0.05	0.074 ± 0.005	35.39 ± 2.10	14.51 ± 3.77	3.30 ± 0.14	0.625 ± 0.039	0.259 ± 0.067	0.066 ± 0.003

		**Open**			**Open**			**Open**			**Open**		
Open	115	1348.59 ± 103.64			1.20 ± 0.07			104.64 ± 5.78			0.095 ± 1.869		
Open	142/144	1515.90 ± 153.71			1.54 ± 0.26			120.69 ± 12.78			0.247 ± 2.179		
Open	156	1477.38 ± 134.60			1.76 ± 0.32			121.44 ± 11.55			0.240 ± 2.238		
Open	202	1320.42 ± 136.20			1.64 ± 0.41			108.03 ± 12.73			0.277 ± 1.994		

No standard error of the mean is provided for Betula young and no data is provided for Picea abies stands on DOY 202 since the spectroradiometer fibre optic cable broke after first measurement set from Betula young stand. Otherwise measurements come from 4 measurement points in each stand.

Otherwise, the UV irradiance trend in shade in the *Q. robur* stand differed from the three *Betula* stands, whereby UV irradiance was higher on DOY 142/144 in the *Q. robur* stand (**Figure 4** and **Figure S10**: non-overlapping CIs on DOY 142/144). However, unlike trends in UV irradiance, no differences in PAR were found between the *Q. robur* and *Betula* stands (**Table 1** and **Figure S10**). Time-course changes in irradiance from the *Q. robur* stand partly stemmed from its delayed canopy phenology compared with that of *Betula* (**Figure S10** and **Figure 5**), albeit this difference was not as distinctly reflected in PAI as it was in sunfleck duration and surveyed phenology (**Figure 5** and **Figure S11**).

### Relating Understorey I_flav_ and Understorey Spectral Irradiance

**Figure 6:**
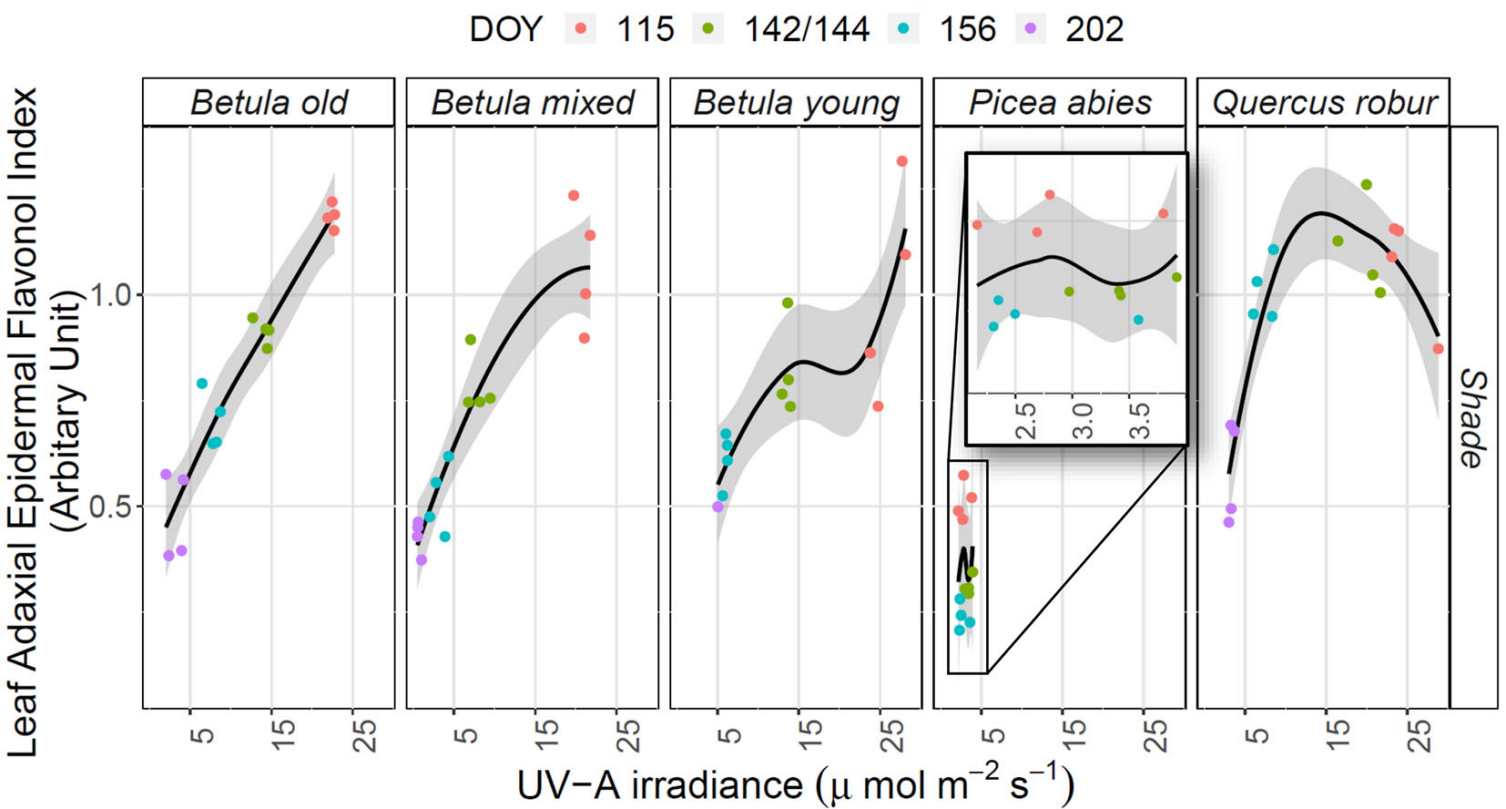
The relationship between mean adaxial flavonol index (I_flav_) and unweighted UV-A irradiance in understorey shade in five different stands (deciduous *Betula* old, mixed and young; evergreen *Picea abies*; deciduous *Quercus robur*) studied during spring and summer 2015. The trend lines are given by a loess fit to the cloud of points with 95% CI (grey band). The degree of smoothing is adjusted for each stand (the default value for parameter α = 1, except in *Betula* mixed α = 2 and in *Betula* young α = 1.1). Different coloured points represent measurements made on different DOYs.

There was a strong positive relationship between mean I_flav_ and spectral irradiance measured in understorey shade (**Table 2** and **Supplementary A2**). On the contrary, the relationship between I_flav_ and spectral irradiance measured in sunflecks or in *leaf* position was mostly weak or non-significant, except for stronger relationship found from the *Betula* stands between I_flav_ and R:FR-ratio measured in sunflecks (**Table 2** and **Supplementary A2**). However, stand-related differences conditioned these relationships; particularly for the *P. abies* stand where spectral irradiance in sunflecks was strongly negatively correlated with I_flav_ (**Table 2**). In understorey shade, of all but the *Q. robur* and *Betula* young stands, a positive linear relationship gave a good fit between different spectral regions and mean I_flav_ (**Figure 6** and **Supplementary A2**).

**Table 2:** The relationship between flavonol index (I_flav_) and spectral irradiance (µmol m^−2^ s^−1^) or effective UV dose calculated according to different spectral weighting functions in different understorey positions.

Stand	Spectral region	Sunfleck		*Leaf* position		Shade	
		*r*	Sig. level	*r*	Sig. level	*r*	Sig. level
*Betula* old	UV-B	0.21	NS	0.41	NS	0.94	***
	UV-A	0.37	NS	0.40	NS	0.96	***
	PAR	0.29	NS	0.31	NS	0.96	***
	FLAV^†^	0.29	NS	0.44	NS	0.95	***
	PG^‡^	0.37	NS	0.42	NS	0.96	***
	GEN(G)^§^	0.05	NS	0.37	NS	0.89	***
	R:FR^¶^	0.61	*	0.62	*	0.94	***
*Betula* mixed	UV-B	0.50	*	0.51	NS	0.91	***
	UV-A	0.57	*	0.51	NS	0.92	***
	PAR	0.42	NS	0.28	NS	0.91	***
	FLAV^†^	0.55	*	0.57	NS	0.91	***
	PG^‡^	0.57	*	0.54	NS	0.91	***
	GEN(G)^§^	0.36	NS	0.39	NS	0.88	***
	R:FR^¶^	0.63	**	0.28	NS	0.92	***
*Betula* young	UV-B	0.50	NS	0.69	*	0.77	**
	UV-A	0.38	NS	0.60	NS	0.82	***
	PAR	0.18	NS	0.53	NS	0.86	***
	FLAV^†^	0.51	NS	0.68	*	0.78	**
	PG^‡^	0.43	NS	0.62	NS	0.81	***
	GEN(G)^§^	0.48	NS	0.70	*	0.70	**
	R:FR^¶^	0.56	*	0.47	NS	0.76	**
*Picea abies*	UV-B	-0.83	**	0.32	NS	0.25	NS
	UV-A	-0.88	***	0.48	NS	0.02	NS
	PAR	-0.87	***	0.46	NS	-0.02	NS
	FLAV^†^	-0.80	**	0.43	NS	0.22	NS
	PG^‡^	-0.88	***	0.48	NS	0.08	NS
	GEN(G)^§^	-0.47	NS	0.23	NS	0.23	NS
	R:FR^¶^	-0.81	**	0.32	NS	0.30	NS
*Quercus robur*	UV-B	0.49	NS	0.56	NS	0.63	**
	UV-A	0.67	**	0.57	NS	0.65	**
	PAR	0.63	**	0.51	NS	0.51	*
	FLAV^†^	0.58	*	0.60	NS	0.67	**
	PG^‡^	0.66	**	0.58	NS	0.67	**
	GEN(G)^§^	0.34	NS	0.55	NS	0.53	*
	R:FR^¶^	0.44	NS	0.32	NS	0.61	*

n = 16, significance levels: * <0.05, **≤0.01, ***≤0.001. Effective doses (µmol m^−2^ s^−1^) calculated according to biological spectral weighting functions for (†) flavonoid accumulation [FLAV action spectrum, (Ibdah et al., 2002)], for (‡) plant growth [PG action spectrum, (Flint and Caldwell, 2003)] and for (§) mathematical formulation for generalised plant action spectrum [GEN(G), (Green et al., 1974)]. The red:far-red photon ratio (¶) was calculated according to Sellaro et al. (2010).

The best selected models used unweighted UV-A irradiation or the effective UV dose calculated according to BSWF for plant growth (PG action spectrum, Flint and Caldwell, 2003) which spans the UV-B and UV-A regions, as explanatory variables for the changes in I_flav_ in understorey shade (all details in **Supplementary A2** and **Table 2**). Model testing for understorey sunflecks gave similar results to shade, except for the *P. abies* stand where contrary to all other stands there was a negative relationship between I_flav_ and spectral irradiance in sunflecks (**Table 2** and **Supplementary A2**). Testing several models with co-linear explanatory variables can lead to error propagation, so these results should be treated with caution, and the relationships do not imply causation.

## Discussion

### Does UV Radiation Explain the Trends in I_flav_?

We found clear stand-related differences in I_flav_ of understorey plants, which were the result of intraspecific and to a lesser extent interspecific differences among the plant communities in these contrasting stands. In species with overwintering leaves, this stand-level divergence was also evident in autumn. Furthermore, we found a seasonal trend in I_flav_ of understorey plants which was consistent among the stands, and which persisted over two consecutive years despite their differing spring weather. All these trends point towards a strong role for environmental factors driving the differences in I_flav_ especially during spring and autumn. Stand-related trends in I_flav_ were related to spectral irradiance in deciduous stands, particularly to effective UV dose calculated according to BSWF for plant growth (PG) and unweighted UV-A, measured in understorey shade. However, the seasonally higher values of I_flav_ found during early spring in plants growing in the evergreen *P. abies* stand were not related to spectral irradiance in spring. We might have expected seasonal changes in I_flav_ to have correlated better with UV-B than UV-A radiation, since seasonal variation in UV-B is more pronounced than that of UV-A (Verdaguer et al., 2017), but this was not the case. Our results resemble the seasonal trend in flavonoids from unshaded leaves of *Betula pubescens* trees growing in Finland, in which high early season values could not be explained by UV radiation alone (Kotilainen et al., 2010). Likewise, flavonoid-glycosides from leaves of *B. pubescens* subsp. *czerepanovii* growing close to the treeline followed a seasonal time-course from bud burst to senescence (Riipi et al., 2002). Similarly, seasonal changes in the short-term accumulation of flavonoids in *Arabidopsis* were also found to persist even when UV was attenuated (Coffey et al., 2017). The trends reported in these studies and ours all point to other environmental factors or developmental processes that co-vary seasonally with UV radiation also being implicated in driving trends in flavonoids (Liakoura et al., 2001; Kotilainen et al., 2010; Nenadis et al., 2015; Coffey and Jansen, 2019). In the next sections we try to disentangle these possible mechanisms affecting epidermal UV absorbance and flavonoid accumulation seasonally.

### Potential Interactions of Seasonal Changes in Temperature and Solar Radiation With Trends in I_flav_

The seasonal I_flav_ trend for higher values in spring and autumn, is likely partly explained by low temperatures experienced by understorey species in these periods. Our I_flav_ trends resembled the seasonal patterns in flavonoids attributed to temperature in an outdoor experiment attenuating UV radiation from different *Arabidopsis* accessions and genotypes (Coffey et al., 2017; Coffey and Jansen, 2019). Earlier studies have found changes in epidermal UV-transmittance at moderate temperatures ranging from + 9 to 21°C (Bilger et al., 2007), which likely falls within the range of summertime fluctuations in the stands we studied (e.g. in 2016 on DOY 166: +5.2–19.8°C outside the forest). However, we did not find that I_flav_ increased with low summer air temperatures (**Figure S12**) and likewise the minimum air temperature on measurement days had poor explanatory value in our data both for seasonal changes in adaxial and abaxial I_flav_ (**Supplementary A2**). Low temperature-enhanced flavonol synthesis is dependent on light (Bhatia et al., 2018), and although this could have relevance especially in high latitudes with extended daylight hours during summer, it seems unlikely to be the only mechanism producing the differences in I_flav_ between adaxial and abaxial leaf sides that we found (**Figure S6**). A combination of harsh environmental conditions, including excessive irradiance and low temperatures during early spring, could feasibly explain the trends in I_flav_, as reported for partially exposed plants compared to those under snowpack in winter (Solanki et al., 2019). This implies that differences in snow cover and winter PAI between the stands in our study might to some extent explain early spring stand-level differences in I_flav._

### Understorey Species-Specific Patterns in I_flav_

Interspecific differences in I_flav_ among understorey species were significant throughout the season, and variation in I_flav_ was found in early spring species as well as in later emerging species, whereby most later emerging species did have low I_flav_ values, but not necessarily lower than other understorey species in a stand on the same DOY. We found seasonal patterns and plasticity in both species with summer green and overwintering leaves, although I_flav_ and leaf flavonoid values remained relatively high in *H. nobilis* meaning that its seasonal or stand-related trends were not as drastic as those of most summer green species (**Figures S7** and **S9**). Even so, the high early spring I_flav_ values of understorey plants found in the *P. abies* stand may be partly attributed to species composition, whereby the *P. abies* stand had a higher proportion of species with overwintering leaves compared to other stands. Similarly, the differences between I_flav_ of plants growing in the *Betula* and *Q. robur* stands were no longer evident when expressed as CWMs, potentially because of understorey species with differing phenological strategies growing in these stands.

Differences in the phenolic profiles of understorey species probably partly explain the species-specific relationships between I_flav_ and mean absorbance of flavonoid extracts in the UV-A and UV-B region, and some of the seasonal heterogeneity in this relationship (**Supplementary A2**). Quantitative measures of total flavonoids often do not reflect qualitative compositional changes (Markham et al., 1998; Stark et al., 2008) which can be functionally meaningful. Furthermore, UV-B -screening was found to be consistent compared to UV-A -screening across multiple species (Robberecht et al., 1980), and within both sun and shade leaves (Liakoura et al., 2003) which might relate to qualitative differences. Nevertheless, the seasonal dynamics in I_flav_ that we report, resembling for instance those of trees (Kotilainen et al., 2010), indicates that qualitative differences alone did not explain species-specific trends in I_flav_ through the growing season.

### Understorey Species-Specific Differences Between Adaxial and Abaxial I_flav_

The relationship between I_flav_ from the leaf adaxial and abaxial side changed seasonally in a species-specific manner (**Figure S6**). The greatest differences between leaf sides were during spring and autumn, suggesting that flavonols located in the epidermis (and possibly immediate subepidermal cells) were primarily responsible for the high I_flav_ during spring, and in autumn for species with overwintering leaves. In contrast, during summer I_flav_ was low and no differences were detected between leaf sides. However, the stronger correlation in most species between flavonoid extracts and I_flav_ during this period, compared to early spring, indicates that in summer epidermal flavonoids (though low) contributed a large proportion of the whole leaf soluble flavonoid content (**Table S10** and **Supplementary A2**). In some circumstances epidermal flavonoids can represent only a small fraction of the whole leaf flavonoids (Csepregi et al., 2019), while in others they can be a major constituent (e.g. Burchard et al., 2000; Bilger et al., 2007). One potential explanation for the seasonally variable relationship that we found between I_flav_ and flavonoid extracts (e.g. in *A. podagraria* during early spring) is that the proportion of the whole leaf flavonoid pool found in the adaxial epidermis changes seasonally. Such a pattern could be created by e.g. flavonoid relocation within the leaf tissue, conformational changes (Barnes et al., 2008; Julkunen-Tiitto et al., 2015) or differing proportions of insoluble phenolics (Semerdjieva et al., 2003). The species-specific differences in I_flav_ of leaf adaxial and abaxial sides may also stem from anatomical differences, e.g. *O. acetosella* which mostly had similar I_flav_ on both leaf sides, has thin leaves and convex epidermal cells focusing direct light to mesophyll (Myers et al., 1994).

### Effects of Leaf Retention Strategy, Leaf Age, and Climatic Factors on Trends in I_flav_

We found that the seasonal trend for decreasing I_flav_ in summer persisted both when comparing leaves of different ages and leaves of standardised age (**Table S8** and **Figure 2**). Although many leaves of summer green species were still immature at the very beginning of the sampling period and leaf age has been found to affect flavonoids in some species (Liakoura et al., 2001; Laitinen et al., 2002), this did not appear to distort our results. Similarly, although in the autumn some old leaves of *A. podagraria* and *H. nobilis* became damaged with age, which could affect the I_flav_, on testing we did not find this to have any major effect on I_flav_. Overwintering leaves of *V. vitis-idaea* maintained high I_flav_ until new leaves with significantly lower I_flav_ were produced (DOY 156/157). Therefore, during spring the relatively large differences between stands in I_flav_ of *V. vitis-idaea* mainly derived from old leaves (**Figure S8**). Hence, while leaf age was an important contributor to the seasonal trend in I_flav_, the flavonoid content of leaves was responsive to the prevailing environment either mainly during leaf development, or throughout leaf lifespan. Seasonal variation in flavonoids and related phenolics have sometimes been attributed to leaf surface features such as pubescent or glabrous leaves, changing with leaf age e.g. in some Mediterranean species (Liakoura et al., 2001). Among the species we surveyed, leaf characteristics may vary, and for instance the overwintering leaves of *F. vesca* have more leaf hairs than leaves produced during summer (Åström et al., 2015). However, the consistent seasonal I_flav_ trend we found from species with varying leaf phenology (e.g. continuous leaf production during spring and summer) suggests that leaf morphological features contributed little to these seasonal trends in leaf flavonoids.

The rate of accumulation of leaf flavonoids often varies over a period of a few days to over a week in response to UV-B radiation (Sullivan et al., 2007; Hectors et al., 2014), but far less is known about the rate of their down-regulation or degradation (Olsen et al., 2009). The noticeable late spring decline in I_flav_ that we report may be partly related to leaf production and the subsequent acclimation of new leaves or individuals to their environment according to their prevailing conditions (e.g. temperature, spectral irradiance, canopy closure). However, the significant differences in I_flav_ between spring and summer (>20 days) among new spring leaves of *H. nobilis*, suggests down-regulation or degradation of flavonoids within a same leaf or individual in this species (**Table S9**).

### Effect of Transient Light in the Understorey on Trends in I_flav_

Mature leaves acclimated to low irradiance can increase their UV-screening when exposed to high irradiance (Barnes et al., 2013; Talhouët et al., 2019), as might happen after canopy leaf fall, compounded by temperature fluctuations, in our deciduous stands. In comparison, leaves acclimated to high irradiance conditions are often found to maintain high UV-screening across various species in nature (Krause et al., 2003; Liakoura et al., 2003), or even when plants acclimated to high irradiance are transferred to conditions with low irradiance (Barnes et al., 2013). This along with our results implies that any damage induced by infrequent high irradiance from sunflecks or sunpatches after canopy closure may be ameliorated by the activation of photoprotection mechanisms independent of flavonoid accumulation. Supporting this assertion, in the evergreen *P. abies* stand the effective UV dose calculated from the spectral irradiance in sunflecks according to BSWF for flavonoid accumulation (Ibdah et al., 2002) indicated a trend of increasing flavonoids towards the highest mid-summer irradiance (**Figure S10**). Although this result is expected in the evergreen stand where understorey irradiance is mostly defined by seasonal changes in solar elevation angle, this increasing trend was the opposite of the decreasing trend in I_flav_ we recorded. It is noteworthy that many brief or less intense sunflecks than those we measured pass through the understorey, and that after canopy closure a larger proportion of the sunflecks will be penumbral compared to direct beam radiation (Chazdon and Pearcy, 1991; Smith and Berry, 2013). Furthermore, overcast weather decreases the occurrence of direct-beam radiation sunflecks in the understorey (Way and Pearcy, 2012). Many plant physiological and morphological features under a closed canopy understorey are acclimated to shaded conditions (Pearcy and Sims, 1994), and more rapid high-light response mechanisms e.g. chloroplast relocation (Williams et al., 2003; Way and Pearcy, 2012) or nastic movement as in *O. acetosella* (Kumke, 1982) may be used during sunflecks to adjust to transient high irradiance. A combination of these explanations could explain why I_flav_ seems not to be adjusted to high irradiance in sunflecks during summer in the understorey species we studied. However, since seasonal variation in I_flav_ was consistent among understorey plant communities, it seems their level of flavonoids was sufficient to minimise damaging effects of high spectral irradiance, or at least that lower flavonoids during summer did not result in a significant trade-off increasing photodamage and repair, and in turn reducing fitness. In line with this, previous studies suggest plants rarely experience severe damage due to UV-B radiation in nature (Paul and Gwynn-Jones, 2003). Furthermore, low UV radiation might provide cross-tolerance to high UV-B radiation (Hideg et al., 2013; Coffey et al., 2017), or high solar irradiance in general (Klem et al., 2015), which might be beneficial during infrequent high irradiance sunflecks or sunpatches.

## Conclusions

We found that consistent seasonal trends and stand-related differences in the epidermal UV-A absorbance of understorey species, reflected climatic conditions, species leaf retention strategy, and new leaf production. Furthermore, we found that understorey plants adjust their epidermal flavonoids to low background shade irradiance compared to infrequent high direct irradiance in sunflecks after canopy closure during summer. Climate change is expected to: 1) affect phenology by extending deciduous canopy cover, hence potentially negatively affecting carbon gain of some understorey plants, and 2) increase the probability of frost damage because of reduced snow cover during winter. In the context of our results, these two effects would result in reduced investment in photoprotective secondary metabolites, such as leaf flavonoids, among understorey species with summer green leaves, but increasing allocation to flavonoids, especially during autumn and winter, in species with overwintering leaves.

## Data Availability Statement

The datasets generated for this study are available on request to the corresponding authors.

## Author Contributions

SH and TR conceived the ideas for the manuscript and designed the field methodology. SH collected and analysed the data. TR analysed the spectroradiometer data. JL collected the additional optical leaf trait dataset in 2016. MP and JL contributed to writing of the manuscript. SH wrote the manuscript, and TR supervised all stages. All authors gave editorial input and final approval for publication.

## Funding

This work was supported by the Academy of Finland (funding decisions 266523, 304519, and 324555).

## Conflict of Interest

The authors declare that the research was conducted in the absence of any commercial or financial relationships that could be construed as a potential conflict of interest.

## Supplementary Material

All the Supplementary Tables and Figures (referred in the main text as **Table S1–S12**, **Figure S1–S12**) are in **Supplementary Appendix 1** (**Supplementary Materials, Data Sheet 1**).

## Abbreviations

BSWF, biological spectral weighting function; CI, confidence interval; CWM, community weighted mean; DOY, day of the year; FLAV, UV action spectrum for accumulation of flavonoids (Ibdah et al., 2002); GEN(G), mathematical formulation of the generalised plant action spectrum (Green et al., 1974); HCAs, hydroxycinnamic acid derivatives; I_flav_, flavonol index measured with an optical leaf clip Dualex Scientific+; LBS, Lammi biological station; PAI, plant area index; PAR, photosynthetically active radiation, 400–700 nm; PG, UV action spectrum for plant growth (Flint and Caldwell, 2003); UV-A, ultraviolet-A radiation, 315–400 nm; UV-B, ultraviolet-B radiation, 280–315 nm.
