# Supplementary material for: Seasonal Patterns in Spectral Irradiance and Leaf UV-A Absorbance Under Forest Canopies
Source: Front Plant Sci. 2020 Feb 18;10:1762. doi: 10.3389/fpls.2019.01762 (PMC7040076; doi:10.3389/fpls.2019.01762)
Supplement: Supplementary file 1 [file DataSheet_1.pdf]

Supplementary Material Appendix 1: Figures and Tables

Order: Tables S1-12, Figures S1-12

Table S1. Details of nine understorey species' strategies for leaf retention, leaf production and form

Species	Growth form	Leaf retention	Leaf production	Leaf form
Anemone nemorosa	Perennial herb	Spring green	Once in lifespan of a ramet	Trifoliolate whorl
Aegopodium podagraria	Perennial herb	Summer green	During growing season	Broadly triangular, 2 times with 3 leaflets
Campanula persicifolia	Perennial herb	Summer green	During growing season	Basal: ligulate, stem: narrowly obovate
Convallaria majalis	Perennial herb	Summer green	Once in lifespan of a ramet	Lanceolate
Filipendula ulmaria	Perennial herb	Summer green	During growing season	Pinnate, 3-5 pairs
Fragaria vesca	Perennial herb	Overwintering leaves	Dimorphic summer/winter leaves	Palmate, 3 leaflets
Hepatica nobilis	Perennial herb	Overwintering leaves	Annual	3-lobed
Oxalis acetosella	Perennial herb	Overwintering leaves	During growing season	Trifoliolate
Vaccinium vitis-idaea	Dwarf shrub	Evergreen	During growing season	Elliptic-obovate

Adjusted from Uemera 1994; Hämet-Ahti et al., 1998 and Åström et al., 2015

Table S2. Stand characteristics around the measurement points and dominant canopy tree species characteristics.

Stand	Mean DBH[†], cm	Mean tree basal area, m^2 ha^{-1}	Mean tree density, ha^{-1}	Mean seedling density, ha^{-1}	Leaf retention strategy	Leaf form
Betula old	22	34	1200	1100	Deciduous	Simple, ovate-triangular
Betula mixed	33	23	900	8250	Deciduous	Simple, ovate-triangular
Betula young	14	22	1550	500	Deciduous	Simple, ovate-triangular
Picea abies	34	39	750	12200	Evergreen	Needle
Quercus robur	31	22	400	9500	Deciduous	Obovate, pinnately lobed

†diameter at breast height

Table S3. Mean monthly air temperature and snowpack evolution from Lammi weather station.

Month	Mean monthly temperature 2015, °C	Mean monthly temperature 2016, °C	Mean monthly snowpack depth 2015, cm	Mean monthly snowpack depth 2016, cm	Beginning of seasons in 2015, DOY	Beginning of seasons in 2016, DOY
1	-3.9	-12.5	18.8	12.2		
2	-1.4	-1.6	31.1	17.3		
3	0.5	-0.7	7.6	15.3[§]	87, spring	85, spring
4	4.2[†]	4.2[†]	[‡]			
5	8.8	12.9				
6	12.4	14.6			167, summer	164, summer
7	15.1	16.6				
8	15.7	14.8				
9	11.5	11				
10	4.3	3.4			276, autumn	275, autumn
11	3.4	-1.9		3.6[§]		
12	1.1	-2.2	-0.7[‡]	4.7	360, winter	362, winter

† Beginning of thermal growing season in 2015 was on 30.4. (DOY 120) and in 2016 on 27.4. (DOY 118). ‡ Final snowmelt in spring 2015 was on 1.4. (DOY 91), first more persistent snowfall was on 18.12. (DOY 352) in winter 2015. § Final snowmelt in spring 2016 was in 29.3. (DOY 89), first more persistent snowfall was on 3.11. (DOY 308) in winter 2016. Seasons were defined as periods when mean daily air temperature was continuously above 0 °C (spring), above +10 °C (summer), below +10 °C (autumn) or below 0 °C (winter).

Table S4. Adaxial epidermal flavonols and anthocyanins, and leaf chlorophyll indices of understorey plants. Presented as unweighted mean (± SE), and for flavonols as community weighted means (CWM) ± SE. Differences in flavonol index, I_{flav} (unweighted means and CWMs) among all stands and only among deciduous stands are presented for each DOY.

DOY	Stand	Flavonol index (AU)†	Chlorophyll index (AU)†	Anthocyanin index (AU)†	*n*	CWMs for I_{flav}	Stands tested	F(df)	Stand-related differences in I_{flav}, sig.	F(df)	Stand-related differences in CWMs for I_{flav}, sig.
114	*Betula* old	1.20±0.03	23.99±1.15	0.19±0.00	74	0.69±0.12	All stands	52.6 (4, 327)	***	1.2 (4, 66)	NS
	Betula mixed	1.08±0.04	24.52±0.99	0.18±0.01	81	0.49±0.13	Deciduous	2.1 (3, 273)	NS	1.4 (3, 56)	NS
	Betula young	1.12±0.04	27.22±1.21	0.17±0.01	64	0.54±0.11					
	Picea abies	0.54±0.02	25.43±1.25	0.15±0.00	55	0.49±0.13					
	Quercus robur	1.12±0.04	24.23±1.15	0.22±0.01	58	0.86±0.20					
125	*Betula* old	1.30±0.03	24.26±0.71	0.17±0.00	112	0.60±0.11	All stands	140 (4, 539)	***	6.4 (4, 91)	***
	Betula mixed	1.20±0.04	23.63±0.81	0.17±0.01	88	0.87±0.14	Deciduous	2.9 (3, 451)	*	3.5 (3, 74)	*
	Betula young	1.20±0.03	24.17±0.86	0.17±0.00	85	0.59±0.11					
	Picea abies	0.54±0.02	25.65±0.95	0.14±0.00	89	0.20±0.04					
	Quercus robur	1.24±0.02	24.17±0.53	0.17±0.00	170	0.40±0.07					
142-144	*Betula* old	0.93±0.02	22.16±0.56	0.14±0.00	161	0.27±0.04	All stands	180.1 (4, 914)	***	4.2 (4, 164)	**
	Betula mixed	0.79±0.02	21.21±0.61	0.14±0.00	180	0.14±0.03	Deciduous	50.8 (3, 773)	***	2.9 (3, 143)	*
	Betula young	0.84±0.02	20.72±0.54	0.15±0.00	184	0.17±0.03					
	Picea abies	0.31±0.02	15.78±0.76	0.15±0.00	142	0.09±0.01					
	Quercus robur	1.12±0.02	22.27±0.46	0.15±0.00	252	0.21±0.02					
156/157	*Betula* old	0.72±0.02	24.15±0.45	0.13±0.00	187	0.19±0.03	All stands	292.4 (4, 994)	***	6.9 (4, 178)	***
	Betula mixed	0.53±0.02	22.34±0.44	0.13±0.00	197	0.09±0.02	Deciduous	150.5 (3, 808)	***	4.0 (3, 149)	**
	Betula young	0.62±0.02	19.46±0.49	0.14±0.00	171	0.13±0.02					
	Picea abies	0.24±0.01	19.00±0.40	0.15±0.00	187	0.05±0.01					
	Quercus robur	1.02±0.02	19.50±0.37	0.15±0.00	257	0.17±0.02					
202/206	*Betula* old	0.49±0.02	24.13±0.52	0.12±0.00	148	0.15±0.03	All stands	35.9 (4, 860)	***	2.0 (4, 150)	NS
	Betula mixed	0.43±0.02	24.60±0.41	0.11±0.00	160	0.10±0.02	Deciduous	11.7 (3, 686)	***	1.1 (3, 121)	NS
	Betula young	0.46±0.02	26.22±0.52	0.12±0.00	151	0.12±0.02					
	Picea abies	0.28±0.01	23.13±0.38	0.12±0.00	175	0.07±0.01					
	Quercus robur	0.58±0.02	19.82±0.34	0.13±0.00	231	0.11±0.01					

† AU= Arbitary Units. Significance levels: * <0.05, **≤0.01, ***≤0.001

Table S5. Stand-related differences in optically measured adaxial epidermal flavonol indices (I_{flav}) of five understorey species from spring to autumn (DOY).

DOY†	*Aegopodium podagraria*		*Anemone nemorosa*		*Fragaria vesca*		*Hepatica nobilis*		*Oxalis acetosella*	
	F (df)	Stands	F (df)	Stands	F (df)	Stands	F (df)	Stands	F (df)	Stands
120	0.6 (1, 23)	NS	19.2 (4, 60)	***	45.3 (4, 44)	***	43.3 (3, 69)	***	3.4 (3, 51)	*
126	7.4 (1, 43)	**	128.3 (3, 86)	***	81.1 (3, 43)	***	20.0 (3, 61)	***	11.0 (3, 76)	***
131	14.2 (1, 28)	***	140.6 (3, 56)	***	103.1 (2, 42)	***	10.5 (3, 56)	***	9.1 (3, 56)	***
133	6.9 (1, 28)	*	148.6 (3, 56)	***	126.3 (2, 42)	***	5.3 (3, 50)	**	44.9 (3, 56)	***
141	0.8 (1, 28)	NS	103.2 (3, 56)	***	25.8 (3, 43)	***	12.7 (3, 56)	***	24.2 (3, 56)	***
145	0.4 (1, 28)	NS	72.6 (3, 56)	***	11.7 (3, 43)	***	2.6 (3, 53)	NS	12.9 (3, 56)	***
154	15.7 (1, 28)	***	80.9 (3, 56)	***	14.8 (3, 43)	***	3.0 (3, 56)	*	3.7 (3, 56)	*
159	4.3 (1, 28)	*	53.7 (3, 56)	***	20.9 (3, 44)	***	3.5 (3, 56)	*	2.9 (3, 56)	*
165	4.0 (1, 43)	NS	127.6 (3, 86)	***	14.9 (2, 42)	***	7.9 (3, 71)	***	2.0 (3, 71)	NS
183			159.6 (1, 28)	***						
195	5.2 (1, 28)	*			0.5 (2, 42)	NS	4.3 (3, 56)	**	2.1 (3, 56)	NS
202	13.6 (1, 28)	***					3.9 (3, 56)	*		
209	8.3 (1, 28)	**			2.9 (2, 42)	NS	10.8 (3, 56)	***	4.4 (3, 56)	**
232	17.0 (1, 28)	***			25.7 (2, 42)	***	9.7 (3, 56)	***	0.9 (3, 56)	NS
244					47.7 (2, 42)	***	10.6 (3, 56)	***	1.4 (3, 56)	NS
258					58.0 (2, 42)	***	11.8 (3, 56)	***	5.5 (3, 56)	**
266	21.8 (1, 28)	***			81.4 (2, 42)	***	11.7 (3, 56)	***	11.2 (3, 56)	***
271	30.5 (1, 33)	***			127.3 (2, 42)	***	14.3 (3, 56)	***	29.9 (3, 56)	***
284	2.5 (1, 28)	NS			129.7 (3, 56)	***	28.6 (3, 56)	***	32.4 (3, 56)	***
292	1.9 (1, 28)	NS			140.0 (3, 56)	***	22.9 (3, 56)	***	59.1 (3, 56)	***

†N.B. Non-equispaced measurement intervals. Significance levels: * <0.05, **≤0.01, ***≤0.001

Table S6. Relationship between optically measured flavonol index (I_{flav}) and temperature or snowpack variables

Year	Measurement	Pearson's correlation coefficient, *r*
2015	Mean daily °C	-0.64***
	Minimum daily °C	-0.59**
	Maximum daily °C	-0.65***
	Days post snowmelt	-0.66***
	Days prior to snowfall	-0.66***
	Days from beginning of TGS†	-0.66***
	Effective temperature sum	-0.62***
2016	Mean daily °C	-0.29**
	Minimum daily °C	-0.41***
	Maximum daily °C	-0.22*
	Days post snowmelt	-0.35***
	Days prior to snowfall	-0.38***
	Days from beginning of TGS†	-0.35***
	Effective temperature sum	-0.38***
2015 and 2016	Mean daily °C	-0.42***
	Minimum daily °C	-0.48***
	Maximum daily °C	-0.37***
	Days post snowmelt	-0.45***
	Days prior to snowfall	-0.48***
	Days from beginning of TGS†	-0.45***
	Effective temperature sum	-0.45***

2015: $n = 25$, 2016: $n = 97$. † Thermal growing season (TGS). Significance levels: * <0.05, **≤0.01, ***≤0.001

Table S7. Differences between stands in flavonol index (I_{flav}) of five understorey species through spring and summer (DOY).

DOY	Species	Mean ± SE *Betula* old	Mean ± SE *Betula* mixed	Mean ± SE *Betula* young	Mean ± SE *Picea abies*	Mean ± SE *Quercus robur*	F (df)	Signifi-cance
114	*Aegopodium podagraria*	1.42±0.035				1.24±0.086	5.4 (1, 34)	*
	Anemone nemorosa	1.02±0.029	1.08±0.044	0.80±0.025	0.71	0.83±0.087	10.6 (4, 67)	***
	Convallaria majalis							
	Filipendula ulmaria	0.96±0.094	1.16±0.118				1.9 (1, 5)	NS
	Oxalis acetosella			0.75±0.060	0.51±0.015		28.4 (1, 35)	***
125	*Aegopodium podagraria*	1.60±0.025				1.61±0.028	0.0 (1, 58)	NS
	Anemone nemorosa	1.23±0.040	1.09±0.039	1.17±0.035	0.53±0.041	1.23±0.036	20.7 (4, 124)	***
	Convallaria majalis							
	Filipendula ulmaria	1.31±0.031	1.20±0.045				4.5 (1, 29)	*
	Oxalis acetosella			0.98±0.043	0.49±0.026		65.3 (1, 42)	***
142-144	*Aegopodium podagraria*	1.16±0.047				1.51±0.031	39.5 (1, 60)	***
	Anemone nemorosa	0.98±0.026	0.75±0.026	0.89±0.022	0.32±0.028	1.15±0.034	80.6 (4, 131)	***
	Convallaria majalis	0.39±0.037		0.40±0.024		0.39±0.019	0.0 (2, 19)	NS
	Filipendula ulmaria	1.09±0.030	0.92±0.070				7.0 (1, 37)	*
	Oxalis acetosella			0.54±0.026	0.23±0.014	0.43±0.024	73.4 (2, 56)	***
156/157	*Aegopodium podagraria*	0.70±0.042				1.22±0.051	62.9 (1, 59)	***
	Anemone nemorosa	0.85±0.036	0.66±0.028	0.81±0.027	0.28±0.020	1.11±0.027	84.0 (4, 136)	***
	Convallaria majalis	0.43±0.023		0.36±0.008		0.60±0.017	93.8 (2, 24)	***
	Filipendula ulmaria	0.91±0.033	0.57±0.043				34.8 (1, 38)	***
	Oxalis acetosella			0.50±0.035	0.22±0.013	0.51±0.037	49.8 (2, 61)	***
202/206	*Aegopodium podagraria*	0.31±0.022				0.45±0.030	12.6 (1, 59)	***
	Anemone nemorosa	0.74±0.052	0.60±0.021		0.21±0.015	0.99±0.106	37.0 (3, 63)	***
	Convallaria majalis	0.43±0.025		0.35±0.017		0.57±0.025	30.1 (2, 23)	***
	Filipendula ulmaria	0.88±0.023	0.35±0.031				169.2 (1, 38)	***
	Oxalis acetosella			0.48±0.043	0.37±0.016	0.47±0.030	5.4 (2, 57)	**

Significance levels: * <0.05, **≤0.01, ***≤0.001

Table S8. Leaf adaxial flavonol index (I_{flav}) from different-aged leaves tested independently for each measurement date when different aged leaves were present

Species	Leaf retention strategy	DOY	Stands included, *n*	F(df)	Significance
Campanula persicifolia	Deciduous leaves	125	1	0.1(1, 18)	NS
		142-144	1	20.2(1, 24)	***
		156/157	1	30.1(2, 9)	***
Convallaria majalis	Deciduous leaves	142-144	2†	56.7(1, 109)	***
Fragaria vesca	Overwintering leaves	125	1	5.3(1, 37)	*
		142-144	2†	62.3(1, 80)	***
		156/157	2‡	31.6(1, 107)	***/NS‡
Hepatica nobilis	Overwintering leaves	142-144	1	22.0(1, 19)	***
Oxalis acetosella	Overwintering leaves	125	1	79.1(1, 34)	***
Vaccinium vitis-idaea	Overwintering leaves	156/157	2†	580.6(1, 25)	***

† Stands were different, but the results for leaf age were similar as shown by separate tests for both stands. ‡ Stands were different and results for leaf age were significant for the other stand, but non-significant for the other stand. Significance levels: * <0.05, **≤0.01, ***≤0.001

Table S9. Differences in adaxial flavonol index (I_{flav}) of *H. nobilis* overwintered leaves between the first measurement in spring and the last measurement during leaf senescence (DOY 120 vs 166). Differences in I_{flav} of newly produced leaves between the time of their emergence and the lowest values reached in summer (DOY 133 vs 154†/195). Student's *t*-test was used when variances were homogenous between the two groups, but Welch *t*-test was used when variances differed. A non-parametric Wilcoxon test was used for non-normally distributed data. Measurements taken within two days of each other were combined.

Stand	Leaf age	Mean on DOY 120/133	*n*	Mean on DOY 166/195†	*n*	*t/W*	Significance
Betula mixed	overwintered leaves	1.16	5	1.06	7	1.13	NS
	new spring leaves	0.71	7	0.59	15	4.46	***
Betula young	overwintered leaves	0.96	21	1.32	5	-7.45	***‡
	new spring leaves	0.83	11	0.61	15	8.43	***
Picea abies	overwintered leaves	0.84	21	0.74	7	2.01	NS
	new spring leaves	0.55	8	0.48	8	51.50	*
Quercus robur	overwintered leaves	1.26	21	1.00	6	3.89	***§
	new spring leaves	0.91	10	0.71	15	2.55	*

† Lowest values of I_{flav} for the *Picea abies* stand were reached on DOY 154. ‡ I_{flav} was significantly higher on DOY 166 compared to DOY 120. § I_{flav} was significantly lower on DOY 166 compared to DOY 120. Significance levels: * <0.05, **≤0.01, ***≤0.001.

Table S10. Species-specific relationship of optically measured flavonol index (I_{flav}) and extracted leaf flavonoids measured at different wavelengths with a spectrophotometer.

Species	Wavelength range	DOY 125		DOY 132		DOY 145		DOY 164		DOY 183		DOY 208		*n*	All dates[†]		*n*
		r	Sig.	*r*	Sig.	*r*	Sig.	*r*	Sig.	*r*	Sig.	*r*	Sig.		*r*	Sig.	
Aegopodium podagraria	375 nm	0.11	NS	0.39	NS	0.37	NS	0.10	NS	-0.22	NS	0.15	NS	15	-0.36	***	90
	UV-B	-0.18	NS	0.14	NS	0.88	***	0.87	***	0.82	***	0.86	***		0.65	***	
	UV-A	-0.24	NS	0.13	NS	0.87	***	0.79	***	0.72	**	0.81	***		0.59	***	
	UV-B & -A	-0.22	NS	0.14	NS	0.88	***	0.83	***	0.77	***	0.83	***		0.62	***	
Anemone nemorosa in *Picea abies* stand	375 nm	0.04	NS	0.35	NS	-0.28	NS	0.62	*	0.19	NS			15	0.81	***	150
	UV-B	0.64	**	0.77	***	0.41	NS	0.86	***	0.82	***				0.90	***	
	UV-A	0.61	*	0.71	**	0.22	NS	0.85	***	0.80	***				0.90	***	
	UV-B & -A	0.63	*	0.73	**	0.29	NS	0.86	***	0.81	***				0.90	***	
Anemone nemorosa in *Quercus robur* stand	375 nm	0.48	NS	-0.26	NS	0.39	NS	0.23	NS	0.42	NS			15			
	UV-B	0.35	NS	-0.28	NS	0.46	NS	0.24	NS	0.65	**						
	UV-A	0.37	NS	-0.28	NS	0.44	NS	0.25	NS	0.66	**						
	UV-B & -A	0.36	NS	-0.28	NS	0.45	NS	0.25	NS	0.66	**						
Convallaria majalis	375 nm			-0.05	NS	0.45	NS	0.20	NS	-0.05	NS	0.09	NS	15	0.33	**	75
	UV-B			-0.02	NS	0.28	NS	0.43	NS	0.60	*	0.77	***		0.47	***	
	UV-A			-0.03	NS	0.43	NS	0.36	NS	0.40	NS	0.42	NS		0.46	***	
	UV-B & -A			-0.03	NS	0.37	NS	0.53	*	0.60	*	0.77	***		0.47	***	
Hepatica nobilis	375 nm	0.48	NS	0.88	***	0.69	**	0.30	NS	0.63	*	0.09	NS	15	0.63	***	90
	UV-B	0.76	***	0.88	***	0.85	***	0.92	***	0.88	NS	0.57	NS		0.88	***	
	UV-A	0.72	**	0.90	***	0.84	***	0.89	***	0.86	***	0.53	NS		0.86	***	
	UV-B & -A	0.74	**	0.89	***	0.84	***	0.90	***	0.87	***	0.54	NS		0.87	***	
Oxalis acetosella	375 nm	0.40	NS	0.59	*	0.46	NS	0.03	NS	0.02	NS	0.52	*	15	0.51	***	90
	UV-B	0.11	NS	0.92	***	0.92	***	0.50	NS	0.49	NS	0.80	***		0.63	***	
	UV-A	0.50	NS	0.85	***	0.80	***	0.54	*	0.35	NS	0.76	**		0.67	***	
	UV-B & -A	0.38	NS	0.88	***	0.85	***	0.53	*	0.39	NS	0.78	***		0.66	***	

† For *A. nemorosa* correlations combining all dates are presented in *Picea abies* stand -section for clarity, although all individuals from both stands are included. Significance levels: * <0.05, **≤0.01, ***≤0.001

Table S11. Differences in *Anemone nemorosa* flavonoids from two contrasting stands measured with Dualex optical leaf clip (flavonol index, I_{flav}) and absorbance of leaf extracts measured spectrophotometrically over different wavelength regions. Differences were tested with Student's *t*-test or non-parametric Wilcoxon test.

DOY	Wavelength region & measurement	Mean in *Picea abies* stand	Mean in *Quercus robur* stand	*t/W*	Signifi-cance
125	I_{flav}	0.54	1.12	0.00	***
	375nm	0.17	0.30	-8.68	***
	UV-B	0.60	1.15	-9.84	***
	UV-A	0.48	0.91	-10.14	***
	UV-B & -A	0.51	0.98	-10.06	***
132	I_{flav}	0.31	1.14	-16.78	***
	375 nm	0.17	0.28	-12.87	***
	UV-B	0.42	1.12	-15.32	***
	UV-A	0.36	0.89	-15.27	***
	UV-B & -A	0.38	0.96	-15.31	***
145	I_{flav}	0.28	0.91	-16.83	***
	375 nm	0.18	0.23	-4.72	***
	UV-B	0.37	0.85	-9.71	***
	UV-A	0.33	0.69	-9.67	***
	UV-B & -A	0.34	0.74	-9.70	***
164	I_{flav}	0.30	0.86	-15.59	***
	375 nm	0.13	0.22	0.00	***
	UV-B	0.41	0.89	0.00	***
	UV-A	0.35	0.69	0.00	***
	UV-B & -A	0.37	0.75	0.00	***
183	I_{flav}	0.29	0.72	-12.63	***
	375 nm	0.17	0.22	-5.86	***
	UV-B	0.48	0.83	-9.05	***
	UV-A	0.39	0.65	-8.98	***
	UV-B & -A	0.42	0.70	-9.03	***

n=15 /DOY/stand. Lack of data on DOY 208 is due to species short life cycle, meaning no plants were present at this time. Significance levels: * <0.05, **≤0.01, ***≤0.001

Table S12. Mean (± SE) photon ratio for UV-B:PAR and effective UV dose (μmol m^{-2} s^{-1}) calculated according to biological spectral weighting function for flavonoid accumulation (FLAV action spectrum, Ibdah et al. 2002) and for the mathematical formulation for generalised plant action spectrum (GEN(G), Green et al. 1974) measured in understorey sunflecks, shade and *leaf* positions, where *leaf* position refers to radiation that is transmitted through the canopy of leaves. Open reference measurements were taken in an open field area well outside the forest.

Stand	DOY	FLAV ± SE			GEN(G) ± SE			UV-B:PAR ± SE		
		Sunfleck	*Leaf* position	Shade	Sunfleck	*Leaf* position	Shade	Sunfleck	*Leaf* position	Shade
Betula old	115	0.31±0.02		0.165±0.001	0.068±0.003		0.036±0.001	0.72±0.09		2.35±0.12
Betula old	142/144	0.28±0.05	0.21±0.01	0.121±0.006	0.068±0.012	0.057±0.004	0.032±0.003	0.88±0.11	1.12±0.06	3.05±0.02
Betula old	156	0.44±0.08	0.27±0.07	0.073±0.005	0.126±0.041	0.079±0.025	0.020±0.002	0.68±0.16	1.03±0.09	3.43±0.29
Betula old	202	0.11±0.02	0.09±0.02	0.028±0.006	0.030±0.010	0.029±0.008	0.007±0.002	0.87±0.16	1.51±0.35	3.61±0.39
Betula mixed	115	0.23±0.03		0.146±0.003	0.051±0.011		0.030±0.002	0.87±0.05		1.85±0.14
Betula mixed	142/144	0.14±0.02	0.07±0.01	0.059±0.006	0.032±0.004	0.012±0.003	0.011±0.002	0.82±0.14	1.39±0.07	2.18±0.29
Betula mixed	156	0.20±0.08	0.07±0.01	0.024±0.003	0.063±0.029	0.015±0.001	0.005±0.002	0.76±0.03	0.94±0.18	1.80±0.52
Betula mixed	202	0.03±0.01	0.01±0.00	0.003±0.001	0.007±0.002	0.000±0.000	0.000±0.000	0.55±0.02	0.28±0.14	0.91±0.45
Betula young	115	0.39±0.03		0.197±0.003	0.093±0.006		0.049±0.002	0.83±0.05	0.00±0.00	2.11±0.29
Betula young	142/144	0.41±0.02	0.26±0.04	0.108±0.005	0.121±0.005	0.072±0.015	0.024±0.003	0.85±0.03	1.01±0.04	2.10±0.12
Betula young	156	0.23±0.02	0.10±0.01	0.050±0.002	0.051±0.004	0.024±0.004	0.010±0.001	0.63±0.03	1.21±0.21	2.66±0.26
Betula young	202	0.22	0.03	0.036	0.033±0.000	0.001±0.000	0.005	0.53	0.24	1.97
Picea abies	115	0.03±0.01		0.020±0.003	0.005±0.001		0.004±0.001	0.84±0.22		2.01±0.13
Picea abies	142/144	0.17±0.02	0.09±0.02	0.022±0.002	0.040±0.005	0.015±0.007	0.004±0.001	0.66±0.03	0.52±0.15	1.49±0.19
Picea abies	156	0.28±0.04	0.04±0.01	0.018±0.002	0.083±0.037	0.005±0.002	0.003±0.001	0.64±0.10	1.29±0.55	3.15±1.08
Picea abies	202									
Quercus robur	115	0.38±0.04		0.165±0.015	0.079±0.021		0.031±0.009	0.67±0.10		1.68±0.16
Quercus robur	142/144	0.47±0.05		0.189±0.012	0.127±0.018		0.056±0.004	0.95±0.02		4.53±0.10
Quercus robur	156	0.23±0.03	0.13±0.03	0.061±0.006	0.054±0.008	0.029±0.007	0.013±0.002	0.61±0.03	0.95±0.10	2.44±0.14
Quercus robur	202	0.21±0.02	0.09±0.02	0.032±0.002	0.063±0.008	0.022±0.008	0.008±0.001	0.83±0.04	0.98±0.07	3.78±0.22
			Open			Open			Open	
Open	115		0.60 ± 0.03			0.143±0.005			0.89 ± 0.04	
Open	142/144		0.74 ± 0.11			0.191±0.042			1.00 ± 0.08	
Open	156		0.82 ± 0.13			0.241±0.055			1.17 ± 0.13	
Open	202		0.74 ± 0.16			0.217±0.079			1.22 ± 0.18	

No standard error of the mean is provided for *Betula* young and no data is provided for *Picea abies* stands on DOY 202 since the spectroradiometer fibre optic cable broke after first measurement set from *Betula* young stand. Otherwise measurements come from 4 measurement points in each stand.

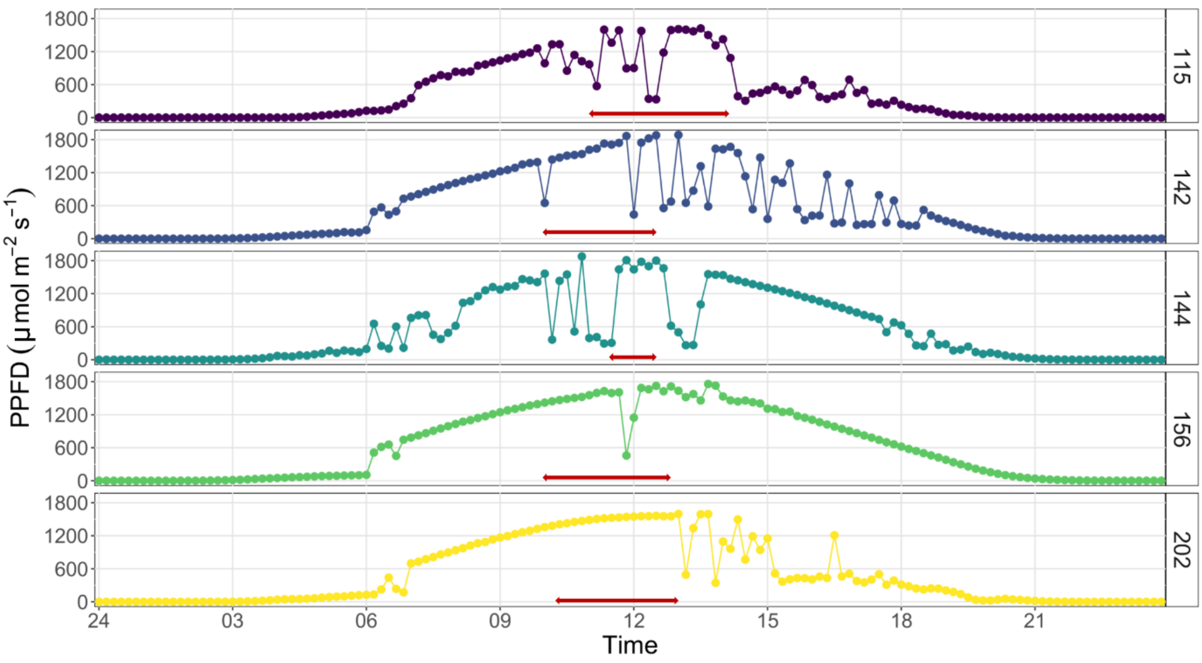

Figure S1. Photosynthetic photon flux density (PPFD) measured at Lammi weather station on the same sampling dates as spectroradiometer measurements i.e. DOY 115, 142/144, 156 and 202 in 2015. These were the closest to clear-sky conditions during the sampling period. Arrows are added to show duration of the spectroradiometer measurement period on a given date. Declines in PPFD are mostly due to clouds, whereas spectroradiometer measurements were made only when no clouds were blocking the sun and sunlight was unobscured. Hence, measurements on DOY 142 were stopped early due to increasing cloud cover and the remaining measurements from the *Picea abies* stand were completed on DOY 144.

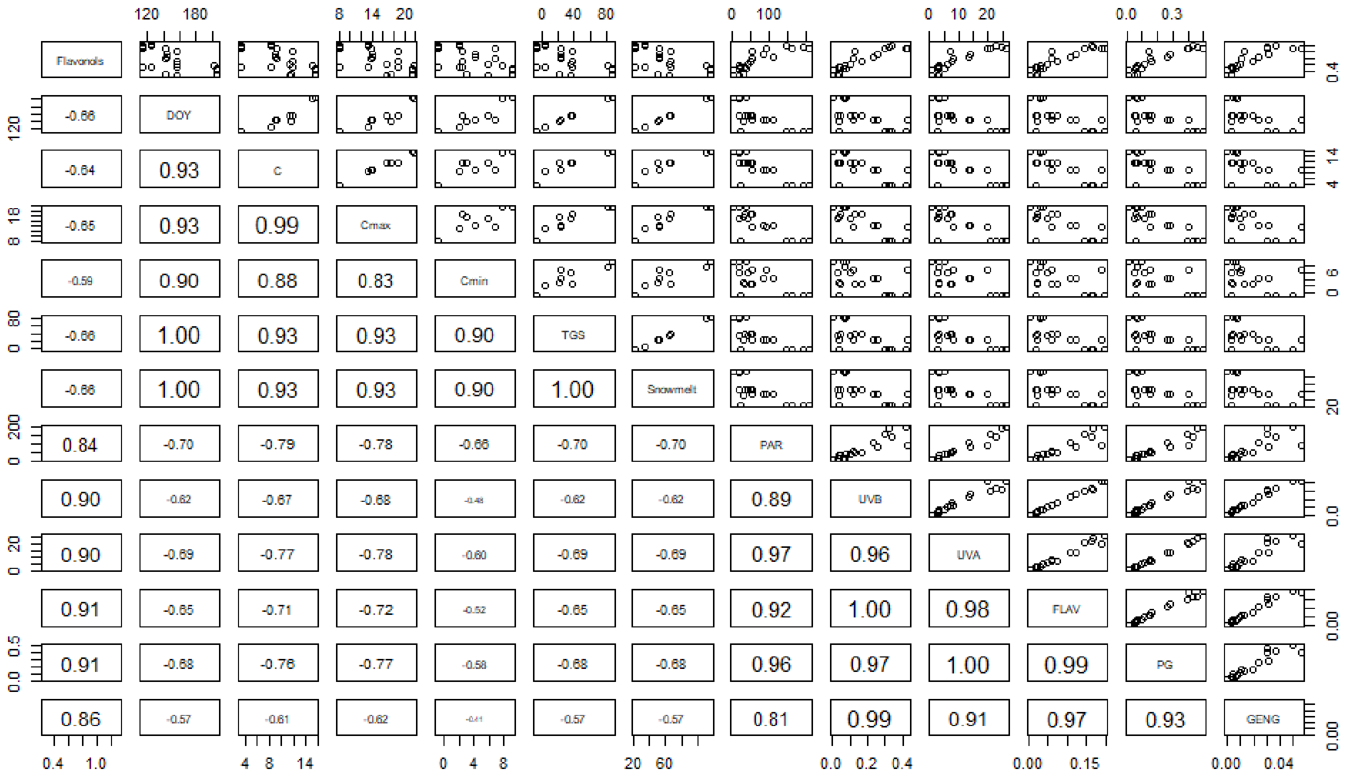

Figure S2: The relationship between mean flavonol index (I_{flav}) over each measurement point (Flavonols) and different climatic factors in 2015. Abbreviations: DOY= day of the year, C=mean daily air temperature, Cmax= maximum daily air temperature, Cmin=minimum daily air temperature, TGS= days from beginning of thermal growing season, Snowmelt = days post snowmelt, PAR = photosynthetic photon flux density (μmol m^{-2} s^{-1}) in understorey shade, UV-B and UV-A irradiance in understorey shade (μmol m^{-2} s^{-1}), and the effective weighted UV doses; FLAV= calculated according to the biological spectral weighting function (BSWF) for flavonoid accumulation (Ibdah et al., 2002), for plant growth BSWF (PG, Flint & Caldwell, 2003) and for the mathematical formulation for generalised plant action spectrum (GENG, Green et al., 1974).

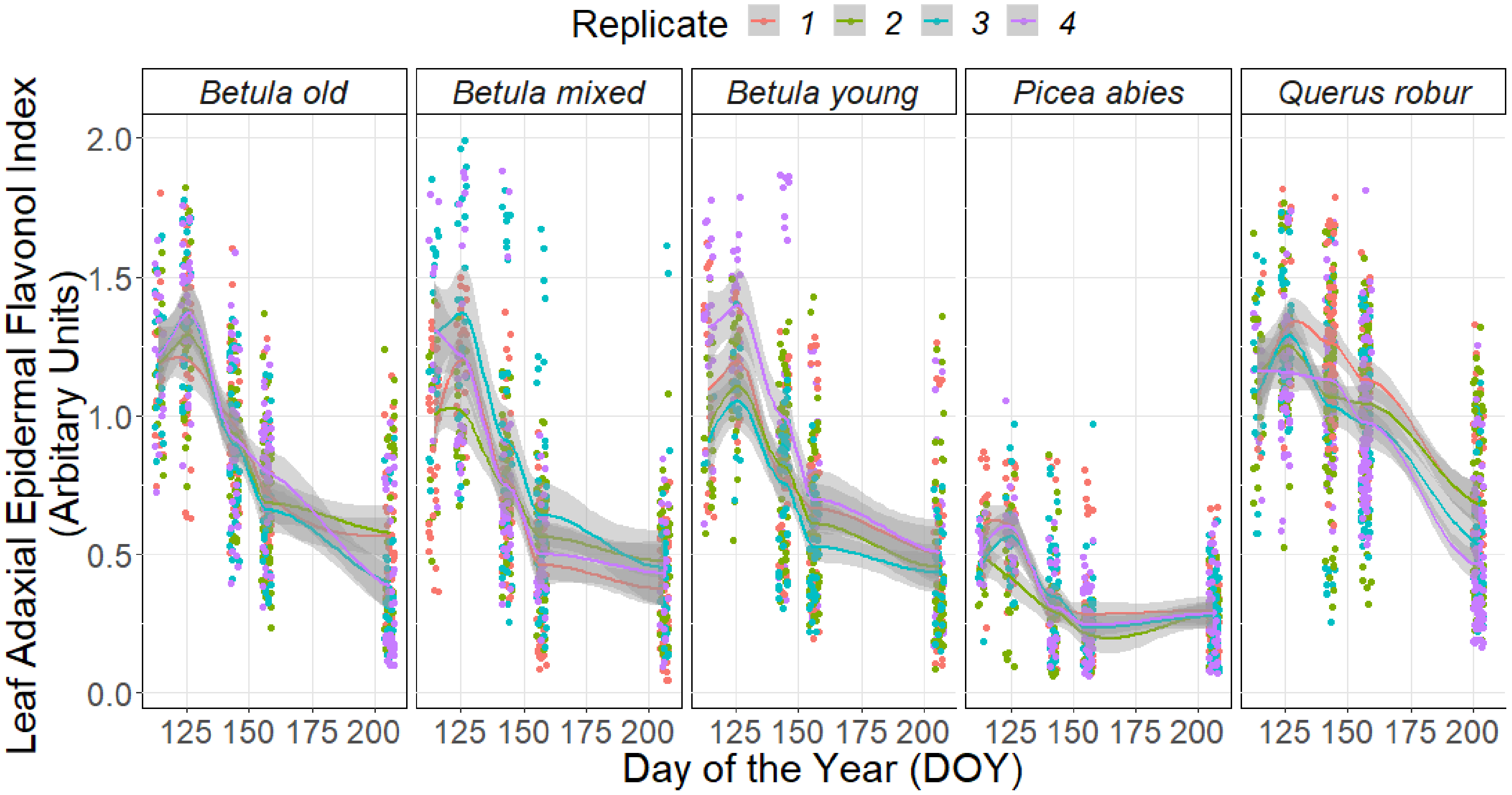

Figure S3. Leaf epidermal flavonol index (I_{flav}) measured during spring and summer 2015 from 35 understorey species present at four measurement points (replicates) in each of the five different stands (deciduous *Betula* old, mixed and young; evergreen *Picea abies*; deciduous *Quercus robur*). The trend lines are given by a loess fit to the cloud of points for each measurement point (replicate) with 95 % CI (grey band).

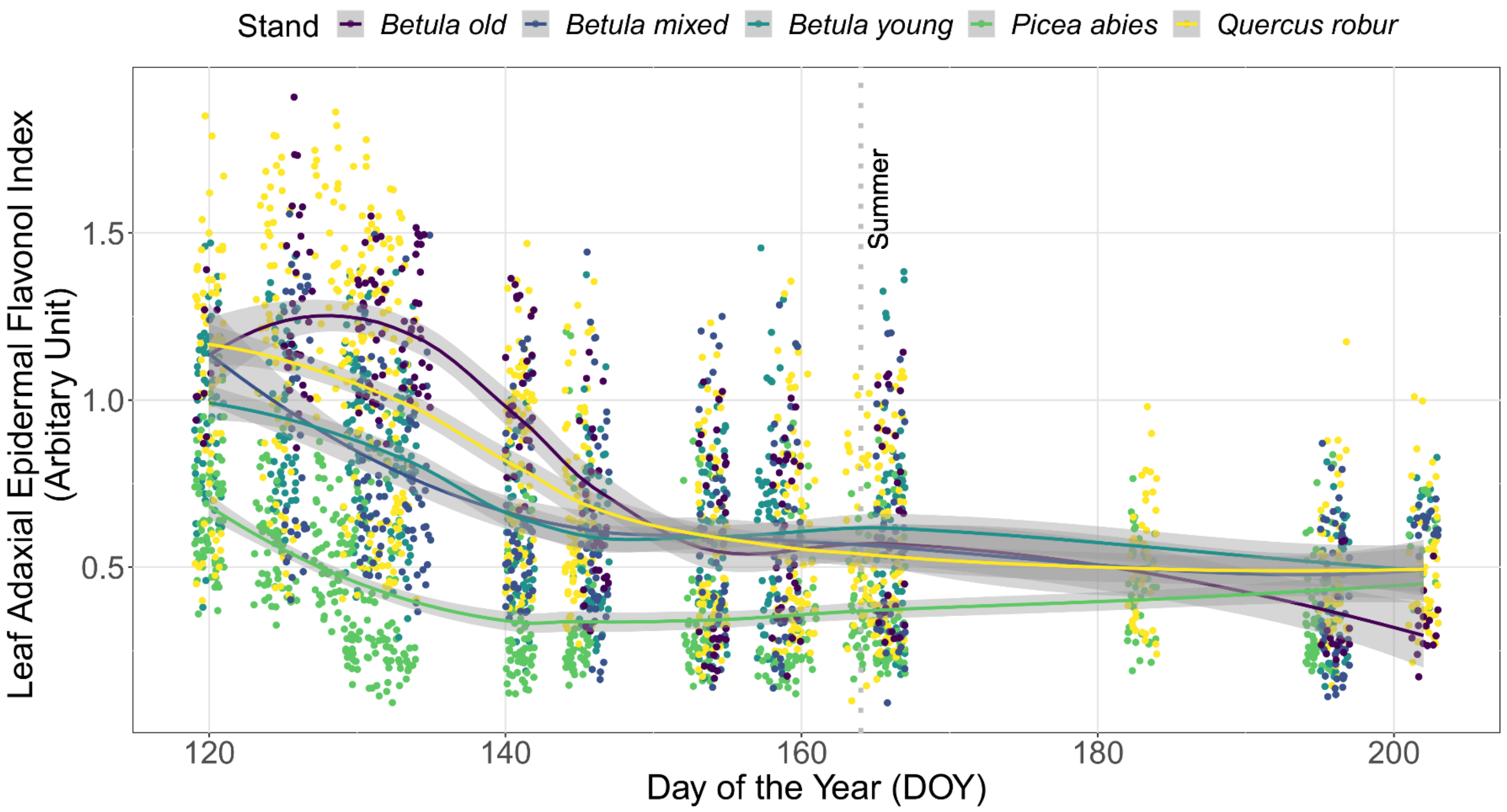

Figure S4. Leaf epidermal flavonol index (I_{flav}) from understorey species growing in five forest stands measured during spring and summer in 2016. A total of 6 understorey species were measured in the different stands. The trend lines are given by a loess fit to the cloud of points for each stand with 95% CI (grey band). Each point represents a measurement from an individual plant. The presented data are cut off on the same final DOY as in 2015 for ease of comparison. The vertical grey dotted line indicates the approximate beginning of summer with respective mean daily air temperatures continuously above +10 ° C degrees.

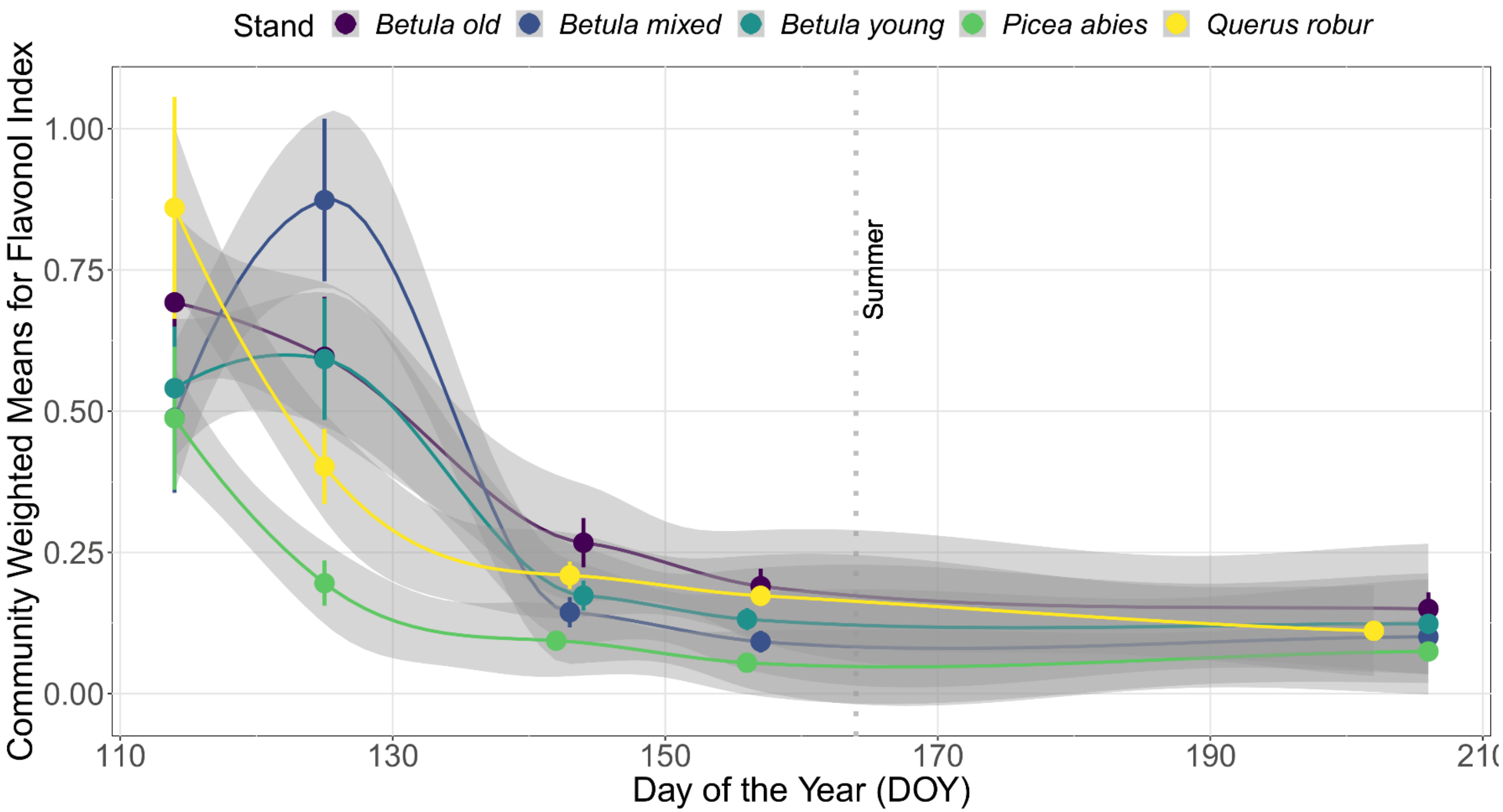

Figure S5. Understorey community weighted means (± SE) of the leaf epidermal flavonol index (I_{flav}) measured on five occasions during spring and summer 2015 from plants growing in five different stands. The trend lines are given by a loess fit to the set of values comprising each point for each stand, with 95 % CI (grey band). The vertical grey dotted line indicates the approximate beginning of summer with respective mean daily air temperatures continuously above +10 ° C degrees.

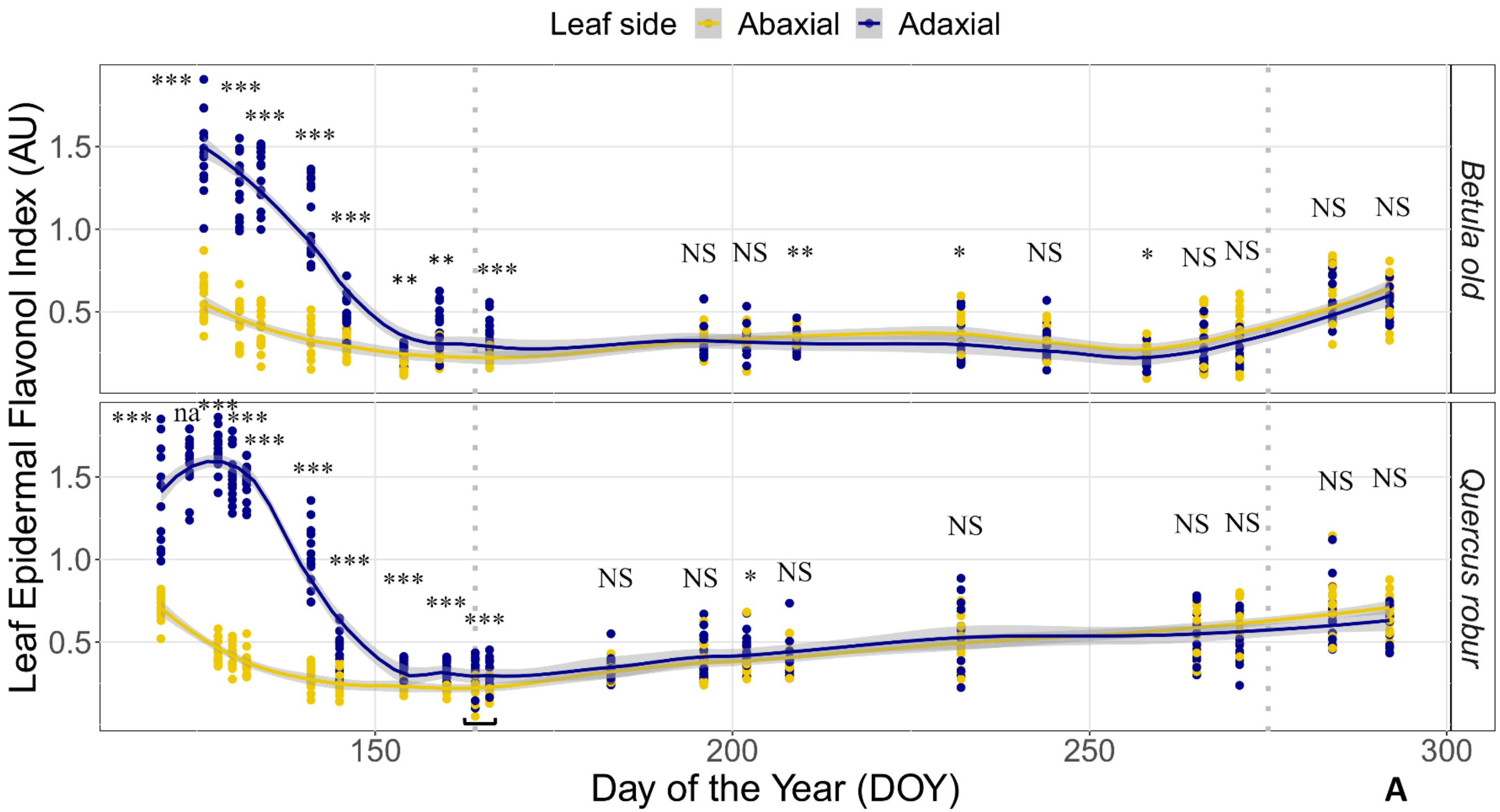
Leaf side
Abaxial
Adaxial
Leaf Epidermal Flavonol Index (AU)
Day of the Year (DOY)
Betula old
Quercus robur
A

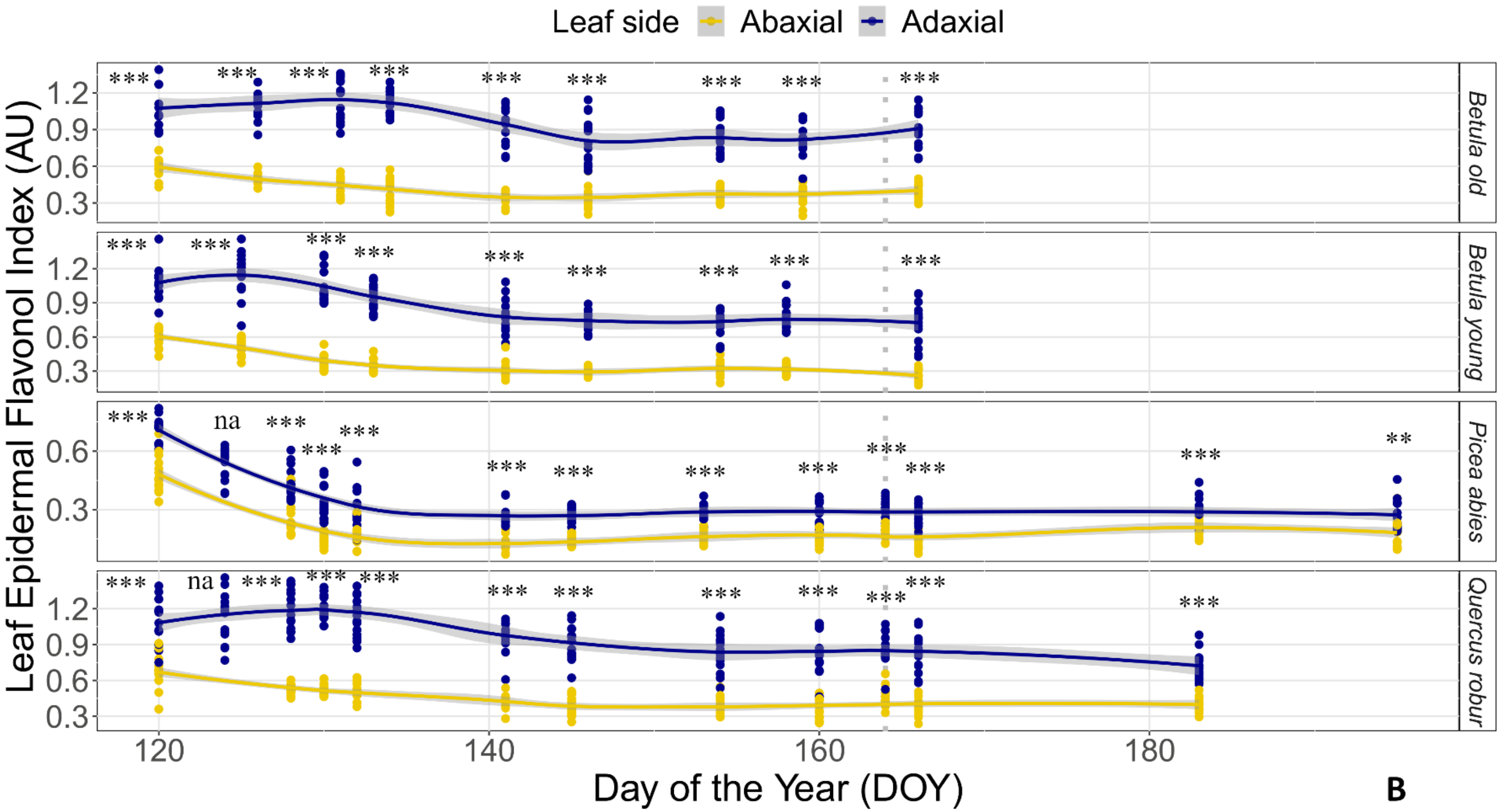

Leaf side
Abaxial
Adaxial
Leaf Epidermal Flavonol Index (AU)
Day of the Year (DOY)
Betula old
Betula young
Picea abies
Quercus robur
na
B

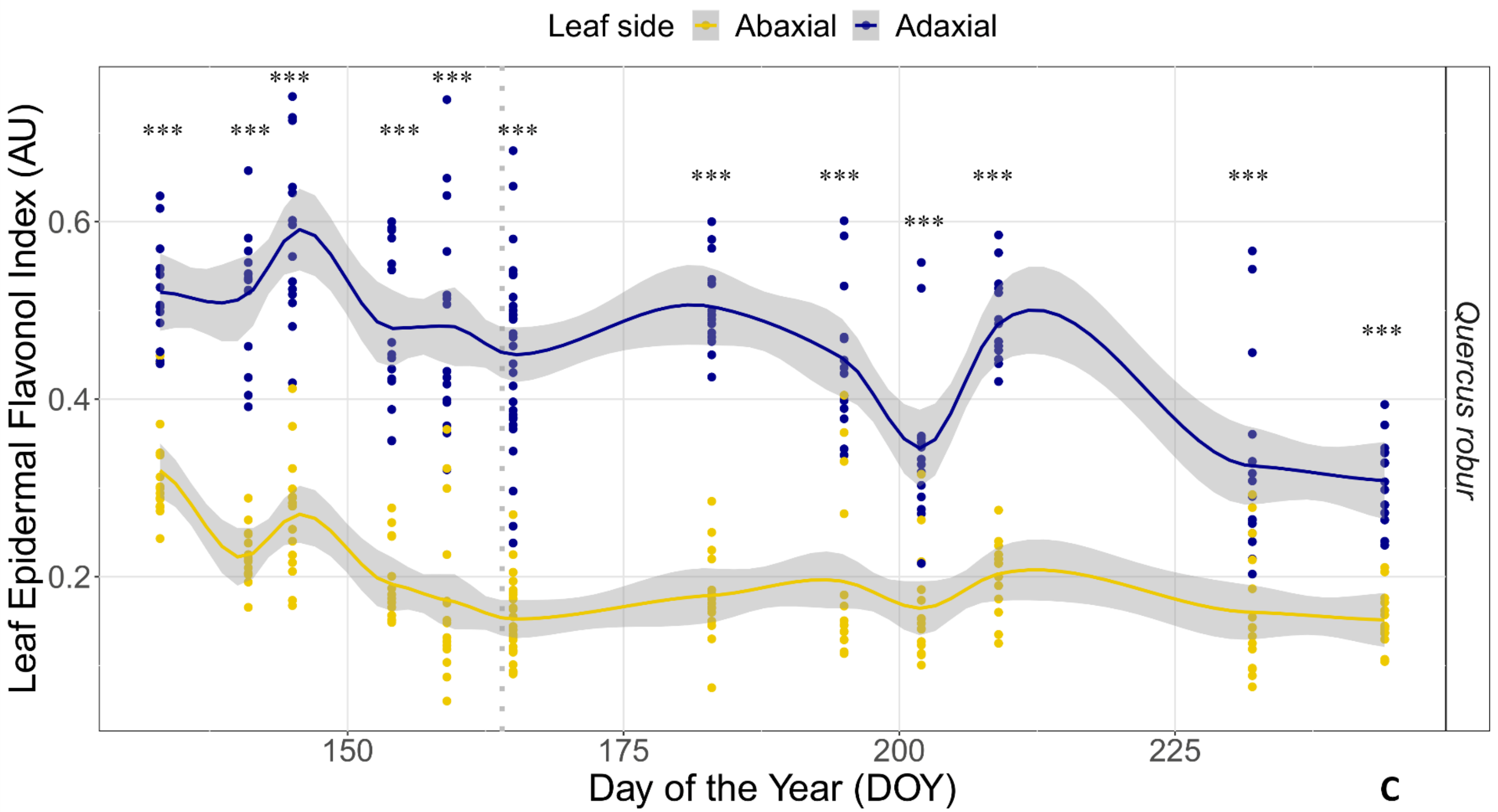
Leaf side
Abaxial
Adaxial
Leaf Epidermal Flavonol Index (AU)
0.6
0.4
0.2
***
Quercus robur
150
175
200
225
Day of the Year (DOY)
C

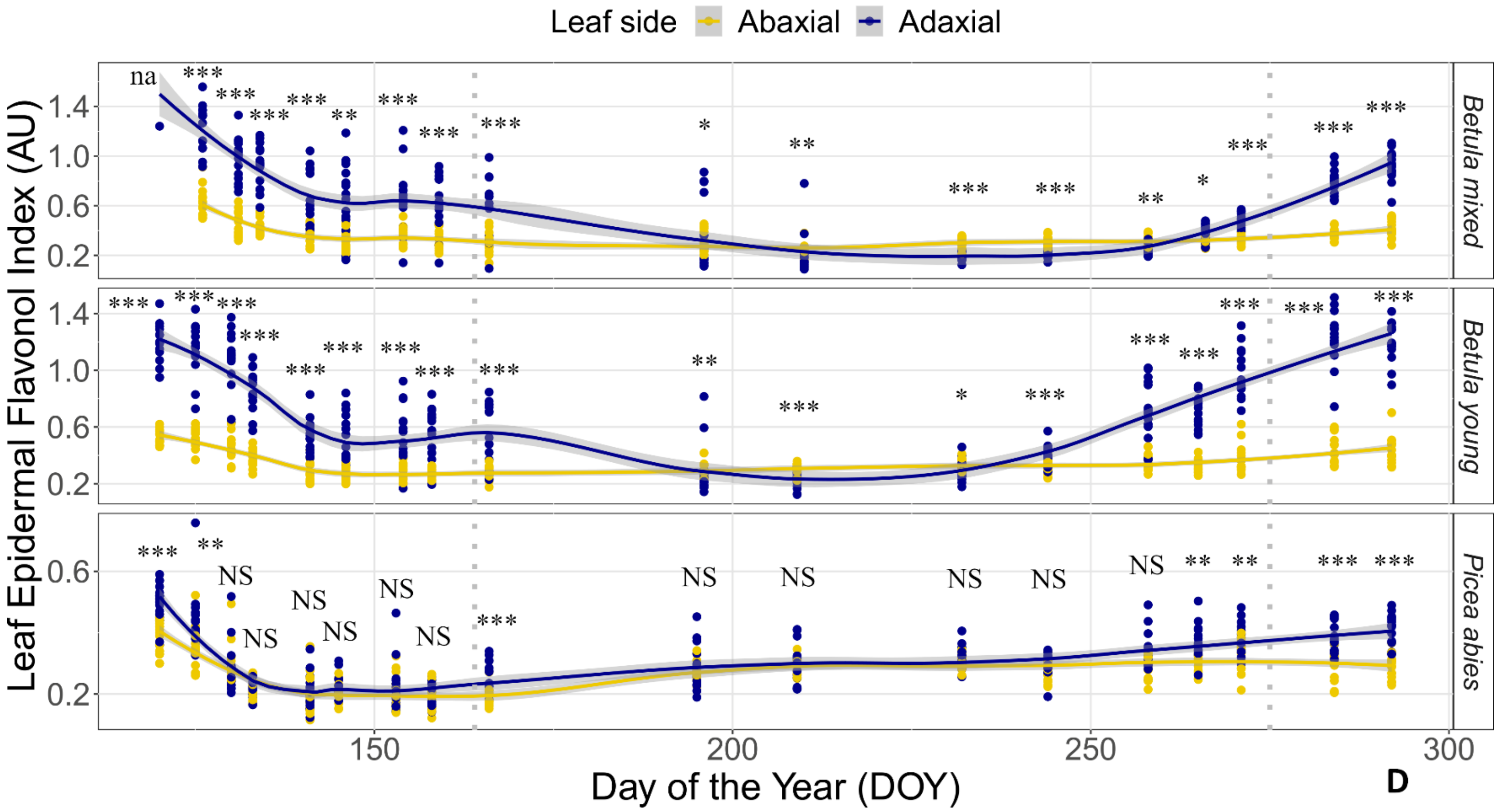

Leaf side
Abaxial
Adaxial
Leaf Epidermal Flavonol Index (AU)
Day of the Year (DOY)
Betula mixed
Betula young
Picea abies
na
NS
150
200
250
300

D

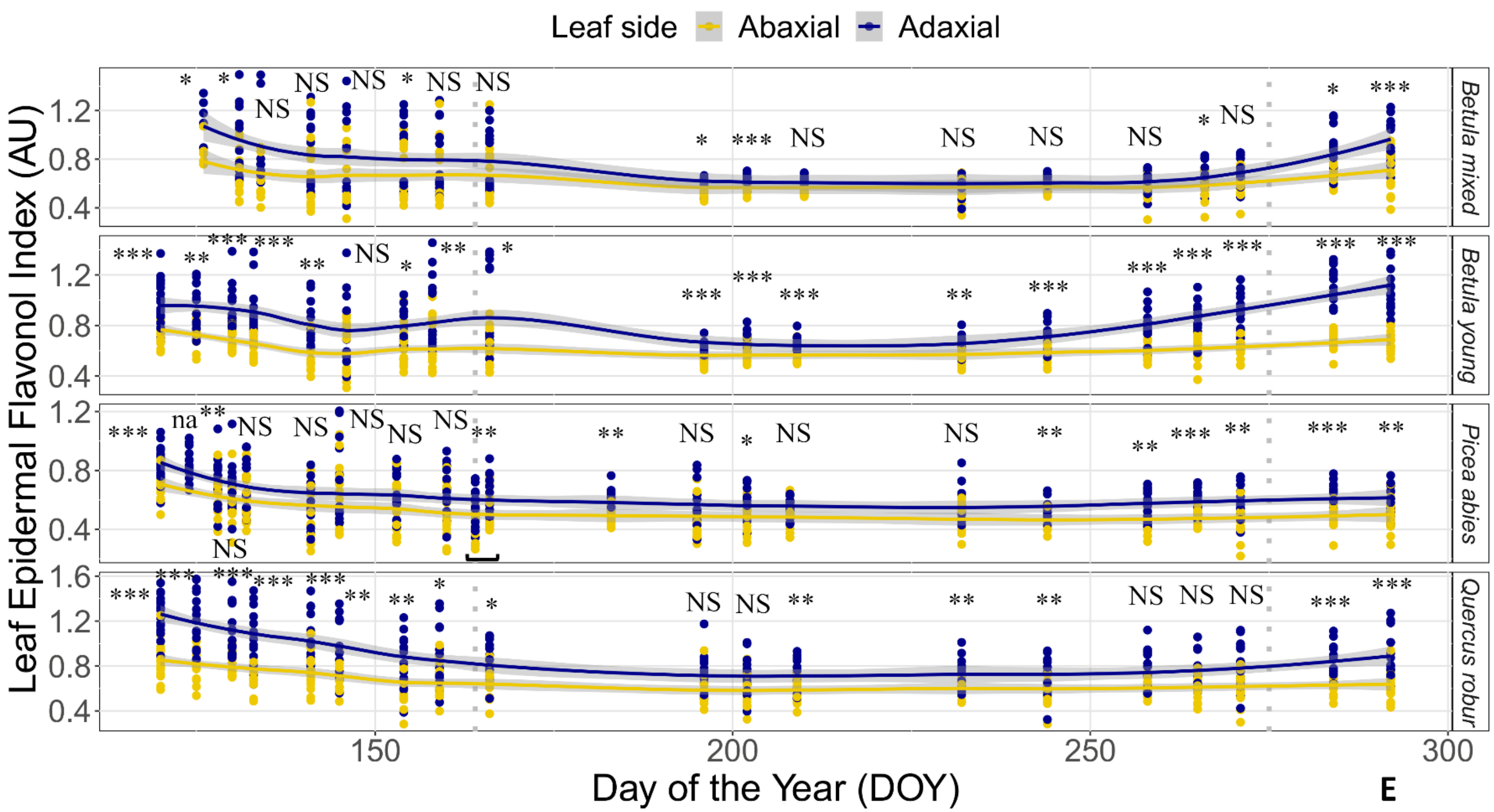

Leaf side
Abaxial
Adaxial
Leaf Epidermal Flavonol Index (AU)
Day of the Year (DOY)
Betula mixed
Betula young
Picea abies
Quercus robur
E

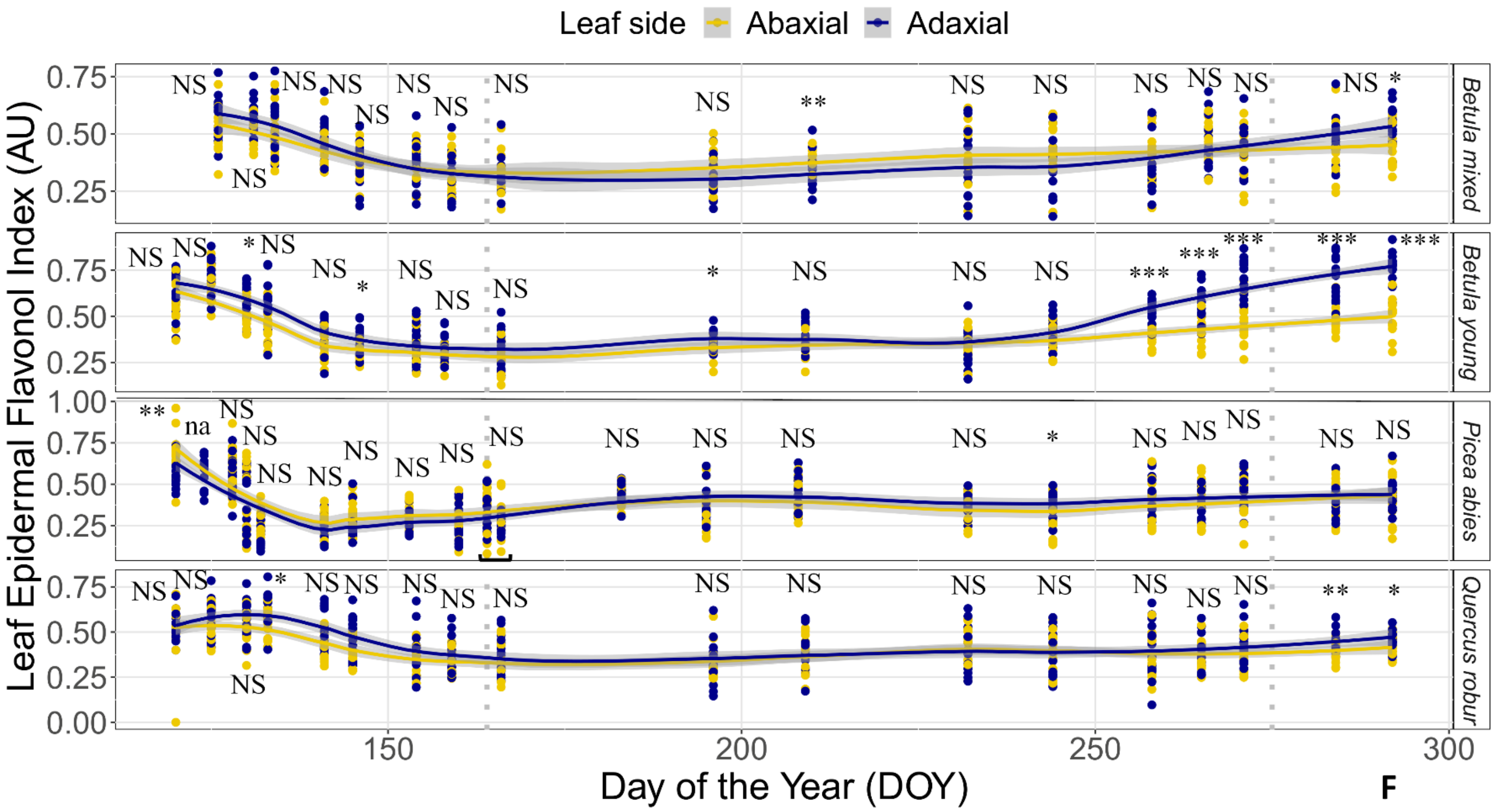

Figure S6. Seasonal changes in flavonol index (I_{flav}, Arbitary Units) of leaf adaxial (blue) and abaxial (yellow) sides from six understorey species (**A**: *Aegopodium podagraria*, **B**: *Anemone nemorosa*, **C**: *Convallaria majalis*, **D**: *Fragaria vesca*, **E**: *Hepatica nobilis* and **F**: *Oxalis acetosella*) growing in different stands (deciduous *Betula* old, mixed and young; evergreen *Picea abies*; deciduous *Quercus robur).* Fifteen individuals from both leaf sides were measured each time from each species from all stands where they were present. Stands with a low sample size are not shown. The trend lines are given by a loess fit, with species-specific adjustments in span, to the cloud of points for each leaf side and 95 % CI (grey band). The vertical grey dotted lines indicate the approximate beginning of summer and autumn with respective mean daily air temperatures continuously above +10 ° C (DOY 164) and below +10 ° C degrees (DOY 275). Significant differences between leaf sides tested with an appropriate test for the data (Student's/ Welch *t*-test or non-parametric Wilcoxon test, see Table S9 caption) are indicated: * <0.05, **≤0.01, ***≤0.001. The y-axis is adjusted to stand-related I_{flav} values.

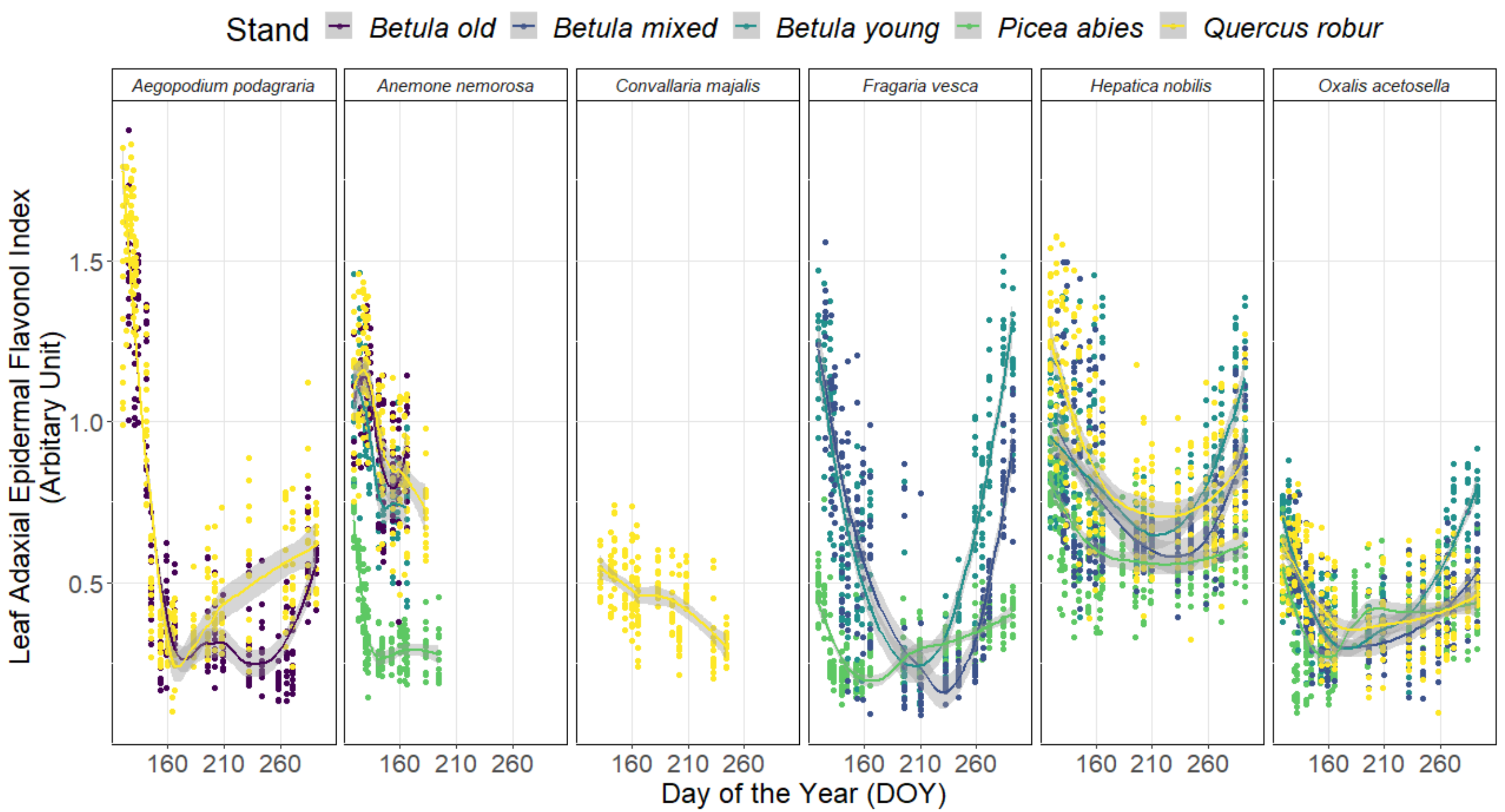

Figure S7. Trends in leaf flavonol index (I_{flav}) of six understorey species (*A. podagraria*, *A. nemorosa*, *C. majalis*, *F. vesca*, *H. nobilis*, *O. acetosella*) measured throughout the growing season of 2016 from different stands. The trend lines are given by a loess fits to the cloud of points for each stand, with each point representing an individual measurement from a plant. The grey band is 95 % confidence interval.

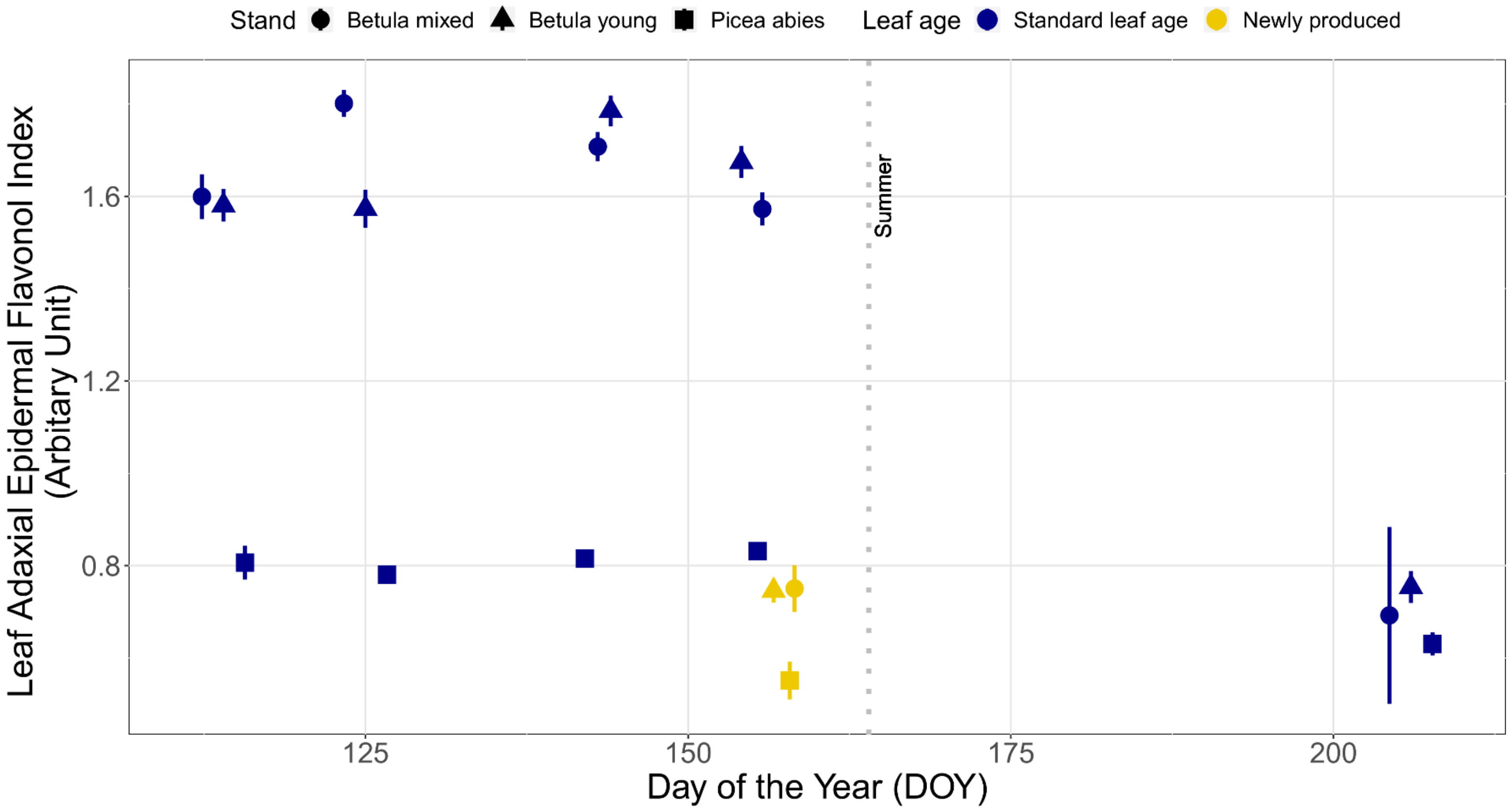

Figure S8. Changes in mean (± SE) flavonol index (I_{flav}) of understorey species *Vaccinium vitis-idaea* from different stands during spring and summer 2015. Sampling on DOY 156/157 was done from individuals with concurrent different aged leaves whereby the standard leaf age refers to first distal adult leaf of the main stem and newly produced leaves refer to yet to be fully matured, bright green leaves. Number of plants measured on DOY 114 = 29, DOY 125 = 30, DOY 142-144 = 23, DOY 156/157 = 36 and DOY 206 = 20. The vertical grey dotted line indicates the approximate beginning of summer with respective mean daily air temperatures continuously above +10 ° C degrees.

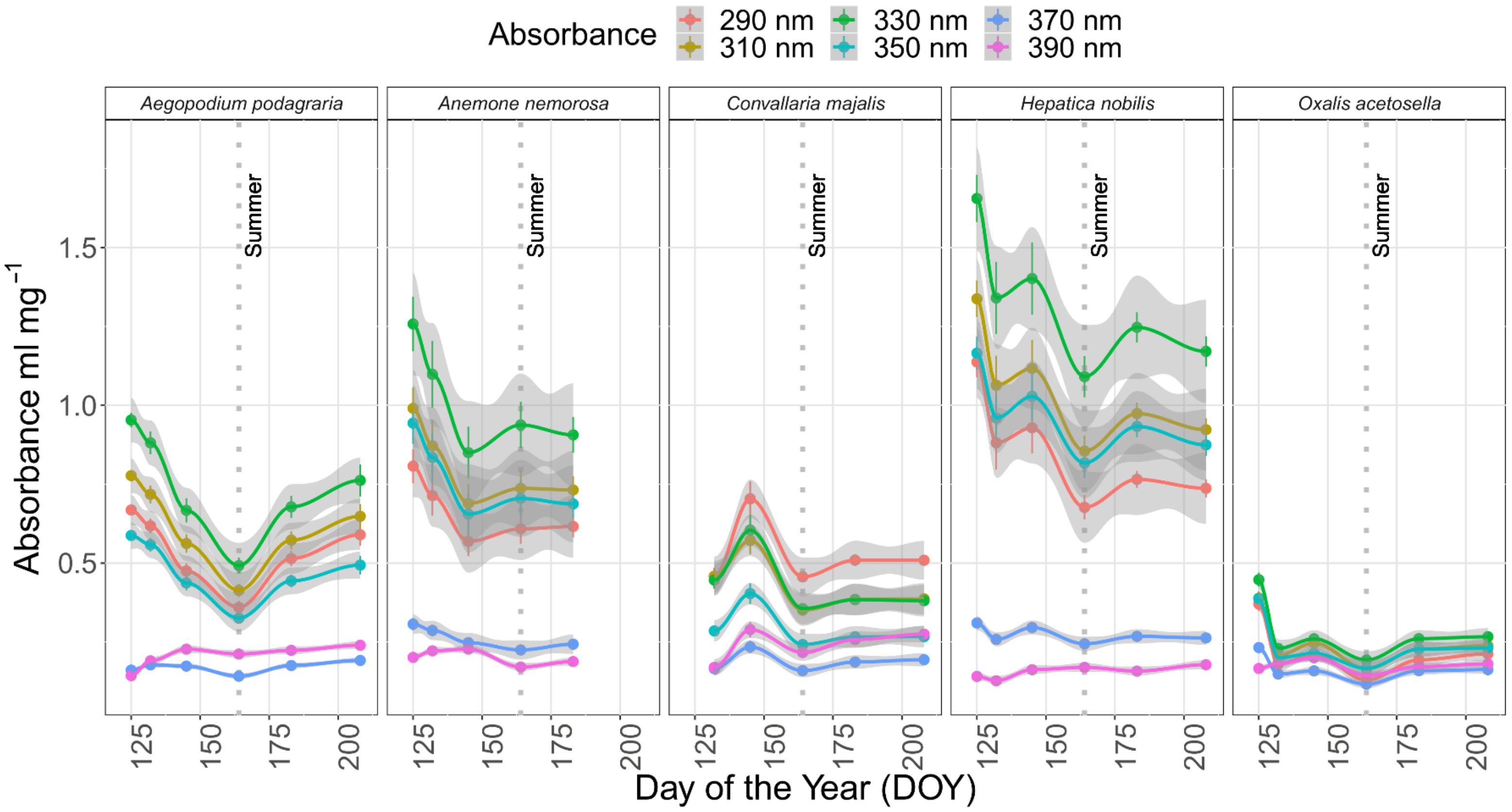

Figure S9: Mean (± SE) absorbance (ml mg $^{-1}$ of fresh weight) of flavonoid extracts from leaves of five understorey species (*n* = 15) during spring and summer 2016. Individuals of *A. podagraria, A. nemorosa, C. majalis* were collected from the deciduous *Quercus robur* stand, and individuals of *A. nemorosa*, *H. nobilis and O. acetosella* were collected from the evergreen *Picea abies* stand. The trend lines are given by loess fits to the set of values comprising each point for each wavelength with 95 % CI (grey band). The vertical grey dotted lines indicate the approximate beginning of summer with respective mean daily air temperatures continuously above +10 ° C degrees.

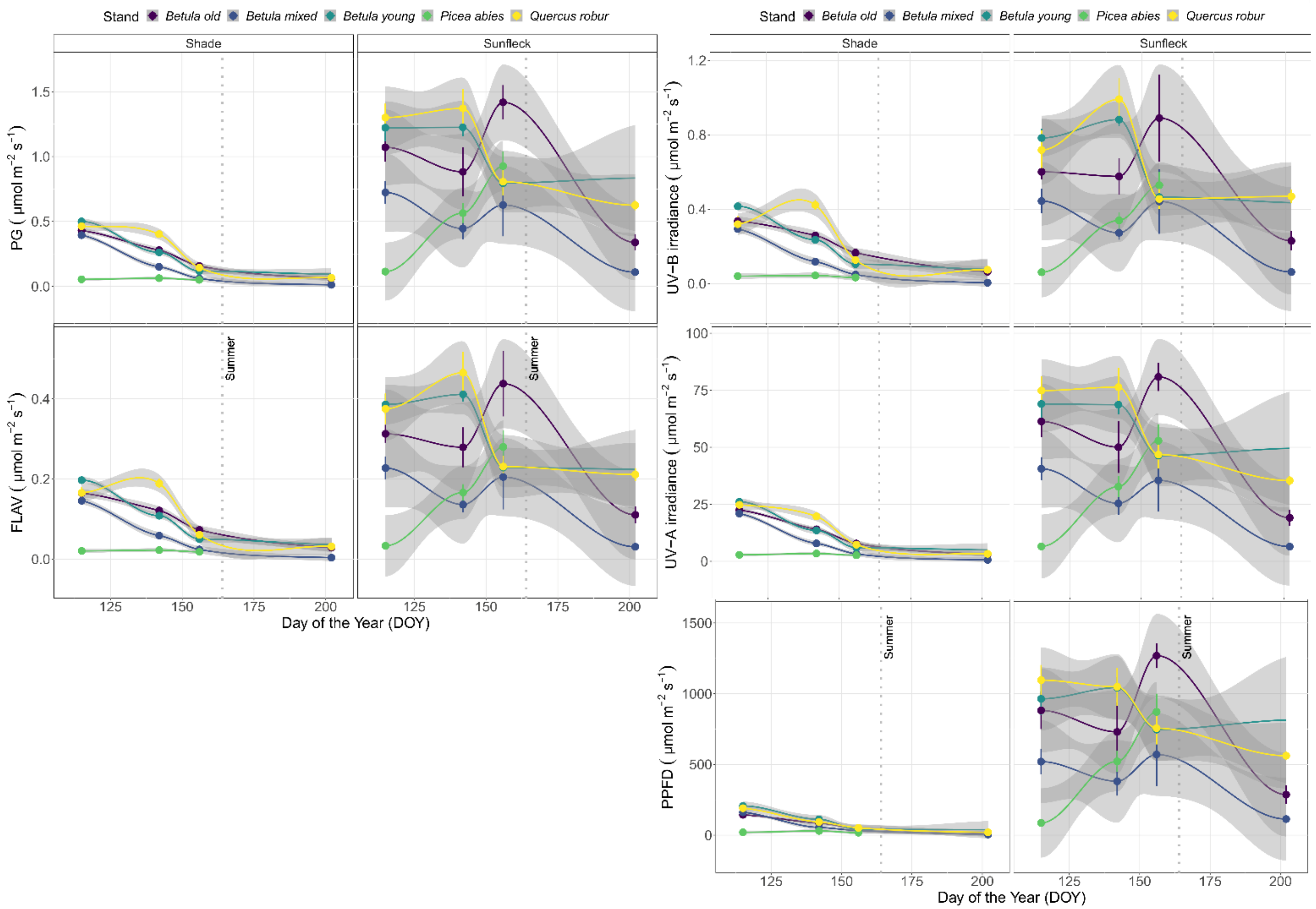

Figure S10: Stand-specific trends in mean (± SE) unweighted UV-B and UV-A irradiance, photosynthetic photon flux density (PPFD), and effective UV doses calculated according to BSWFs for plant growth (PG, Flint & Caldwell, 2003) and flavonoid accumulation (FLAV, Ibdah et al., 2002) in understorey shade and sunflecks during spring and summer 2015. The trend lines are given by a loess fit to the set of values from each measurement point (n = 4) for each stand with 95 % CI (grey band). The vertical grey dotted lines indicate the approximate beginning of summer with respective mean daily air temperatures continuously above +10 ° C degrees. There are no data from DOY 202 for the *Picea abies* stand, nor from 3/4 measurement points in the *Betula* young stand due to the fibre optic cable to the diffuser breaking.

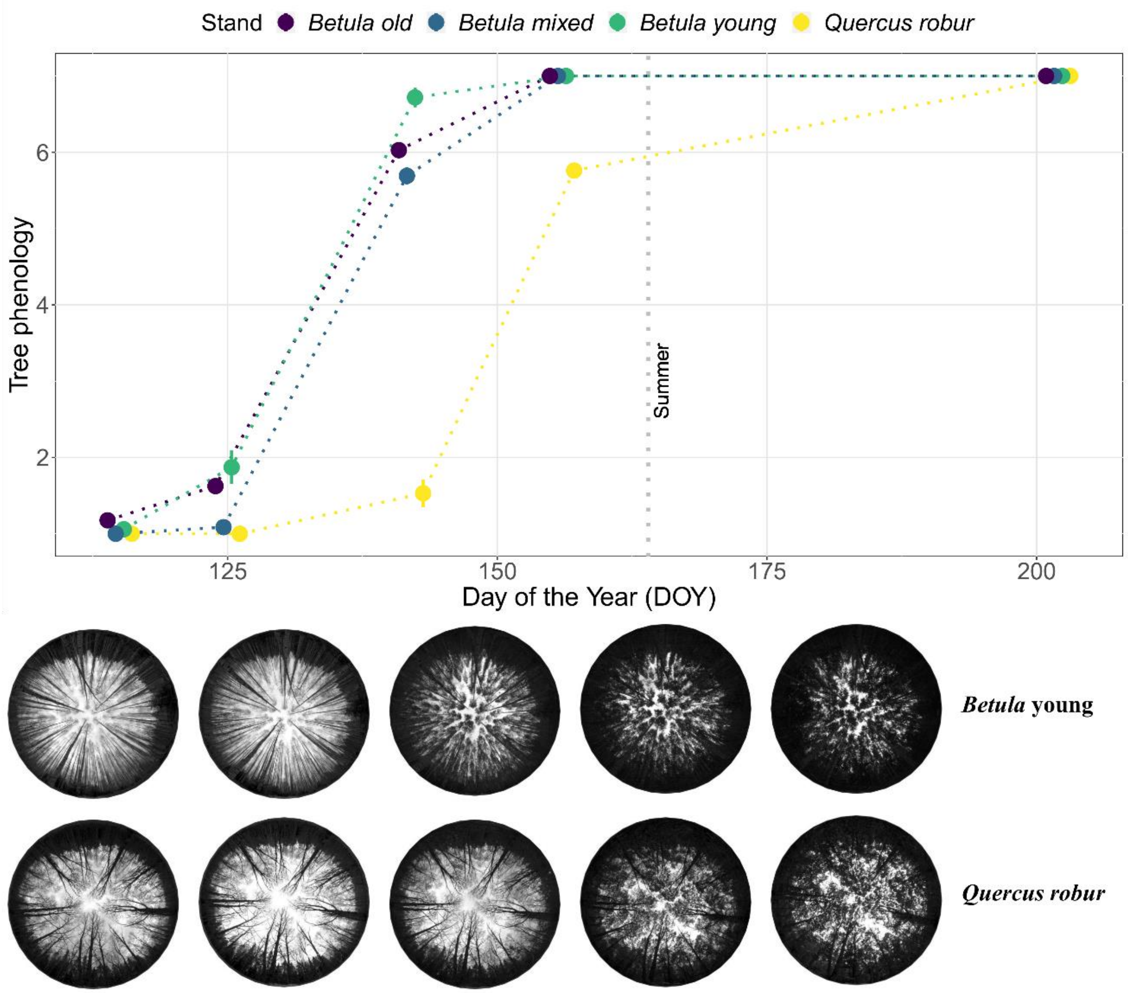

Figure S11. Mean (± SE) tree phenology surveyed from five stands (*n* =12-16) during spring and summer 2015. Tree phenology was surveyed at three canopy heights. The phenology scale ranges from: dormant buds = 1 to closed canopy = 7. Example hemispherical photographs from two stands with differing phenology, taken on the same DOYs as the five phenology surveys were made, are presented below the chart. The vertical grey dotted line indicates the approximate beginning of summer with respective mean daily air temperatures continuously above +10 ° C degrees.

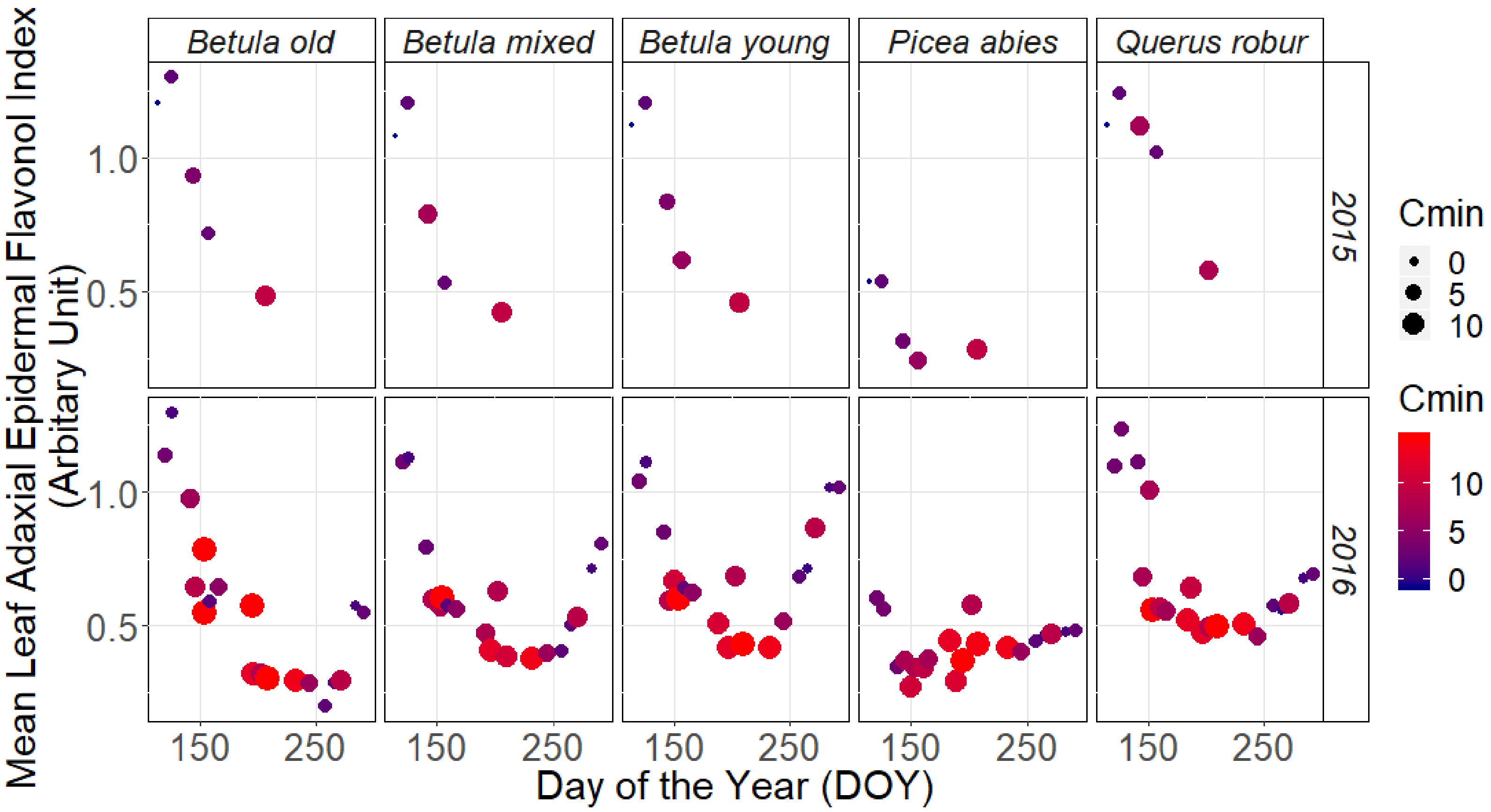

Figure S12: Mean flavonol index (I_{flav}) compared to minimum daily air temperature (°C min; value indicated by colour and size of the points) change with DOY in five different stands over two consecutive years 2015 and 2016. Temperature data is obtained from the LBS weather station outside the forest, hence no stand-related adjustments are made, although the mean I_{flav} is stand-specific.

References

Hämet-Ahti, L., Suominen, J., Ulvinen, T., & Uotila, P. (eds.) (1998). Retkeilykasvio (Field Flora of Finland). Helsinki: Finnish Museum of Natural History, Botanical Museum.

Uemura, S. (1994). Patterns of leaf phenology in forest understorey. *Canadian Journal of Botany*, *72*, 409-414. doi: 10.1139/b94-055.
