# Supplementary material for: Seasonal Patterns in Spectral Irradiance and Leaf UV-A Absorbance Under Forest Canopies
Source: Front Plant Sci. 2020 Feb 18;10:1762. doi: 10.3389/fpls.2019.01762 (PMC7040076; doi:10.3389/fpls.2019.01762)
Supplement: Supplementary file 2 [file DataSheet_2.pdf]

Supplementary Material Appendix 2: Details of the data analyses

1. Repeated optical leaf trait measurements from understorey plants

Modelling the change in time with flavonol index, I_{flav} was complex as repeated measurements of the same leaves on a plant would be expected to be autocorrelated, and similar local light conditions at the measurement points could produce high spatial correlations i.e. within stands/measurement points/species. Consequently, to the sake of simplicity to visually compare trends, we decided use loess-based fits (obtained with R function "loess") to data from different stands with 95% confidence intervals (CI).
It was difficult to closely fit the trends in I_{flav} using a simple function. We tried several approaches, for instance fitting 2nd and 3rd order polynomial functions to trends in I_{flav} with DOY from different stands. Although all the terms were significant at the 5 % level, R^2-values were low ranging from 0.31 to 0.54 between different stands thus indicating a poor fit of the model (A2 Figure S1). Furthermore, visual inspection of the residuals plotted against fitted values showed problems with the model fit of these polynomials.

```
> summary(MBeo) #Betula old

Call:
lm(formula = FlvYKS ~ poly(DOY, 3), data = Beo)

Residuals:
     Min       1Q   Median       3Q      Max 
-0.61423 -0.19647 -0.02335  0.19430  0.75170 

Coefficients:
               Estimate Std. Error t value Pr(>|t|)    
(Intercept)    0.865845   0.009991  86.663  < 2e-16 ***
poly(DOY, 3)1 -6.952780   0.260916 -26.648  < 2e-16 ***
poly(DOY, 3)2  1.849290   0.260916   7.088 3.43e-12 ***
poly(DOY, 3)3  1.771379   0.260916   6.789 2.47e-11 ***
---
Signif. codes:  0 ‘***’ 0.001 ‘**’ 0.01 ‘*’ 0.05 ‘.’ 0.1 ‘ ’ 1

Residual standard error: 0.2609 on 678 degrees of freedom
Multiple R-squared:  0.5433,	Adjusted R-squared:  0.5412 
F-statistic: 268.8 on 3 and 678 DF,  p-value: < 2.2e-16

> summary(MBeob) #Betula mixed

Call:
lm(formula = FlvYKS ~ poly(DOY, 3), data = Beob)

Residuals:
     Min       1Q   Median       3Q      Max 
-0.75694 -0.20260 -0.01394  0.14061  1.18784 

Coefficients:
              Estimate Std. Error t value Pr(>|t|)    
(Intercept)    0.72015    0.01119  64.348  < 2e-16 ***
poly(DOY, 3)1 -6.29844    0.29736 -21.181  < 2e-16 ***
poly(DOY, 3)2  2.77382    0.29736   9.328  < 2e-16 ***
poly(DOY, 3)3  2.06010    0.29736   6.928 9.68e-12 ***
---
Signif. codes:  0 ‘***’ 0.001 ‘**’ 0.01 ‘*’ 0.05 ‘.’ 0.1 ‘ ’ 1

Residual standard error: 0.2974 on 702 degrees of freedom
Multiple R-squared:  0.454,	Adjusted R-squared:  0.4516 
F-statistic: 194.5 on 3 and 702 DF,  p-value: < 2.2e-16

> summary(MBey) #Betula young
```

```
Call:
lm(formula = FlvYKS ~ poly(DOY, 3), data = Bey)

Residuals:
     Min       1Q   Median       3Q      Max 
-0.60075 -0.22208 -0.02675  0.17763  1.00183 

Coefficients:
              Estimate Std. Error t value Pr(>|t|)    
(Intercept)    0.76764    0.01149  66.815  < 2e-16 ***
poly(DOY, 3)1 -5.89320    0.29404 -20.042  < 2e-16 ***
poly(DOY, 3)2  2.18374    0.29404   7.427 3.51e-13 ***
poly(DOY, 3)3  1.72128    0.29404   5.854 7.62e-09 ***
---
Signif. codes:  0 ‘***’ 0.001 ‘**’ 0.01 ‘*’ 0.05 ‘.’ 0.1 ‘ ’ 1

Residual standard error: 0.294 on 651 degrees of freedom
Multiple R-squared:   0.43,	Adjusted R-squared:  0.4274 
F-statistic: 163.7 on 3 and 651 DF,  p-value: < 2.2e-16

> summary(MPic)  #Picea abies

Call:
lm(formula = FlvYKS ~ poly(DOY, 3), data = Pic)

Residuals:
     Min       1Q   Median       3Q      Max 
-0.39786 -0.11196 -0.02046  0.07906  0.73904 

Coefficients:
              Estimate Std. Error t value Pr(>|t|)    
(Intercept)     0.3350     0.0063  53.183  < 2e-16 ***
poly(DOY, 3)1  -1.8066     0.1604 -11.266  < 2e-16 ***
poly(DOY, 3)2   2.0405     0.1604  12.724  < 2e-16 ***
poly(DOY, 3)3   0.4429     0.1604   2.762  0.00591 ** 
---
Signif. codes:  0 ‘***’ 0.001 ‘**’ 0.01 ‘*’ 0.05 ‘.’ 0.1 ‘ ’ 1

Residual standard error: 0.1604 on 644 degrees of freedom
Multiple R-squared:  0.3152,	Adjusted R-squared:  0.312 
F-statistic: 98.82 on 3 and 644 DF,  p-value: < 2.2e-16

> summary(MQrob) #Quercus robur

Call:
lm(formula = FlvYKS ~ poly(DOY, 3), data = Qrob)

Residuals:
     Min       1Q   Median       3Q      Max 
-0.88653 -0.18393 -0.01151  0.17778  0.80369 

Coefficients:
               Estimate Std. Error t value Pr(>|t|)    
(Intercept)    0.984381   0.008928 110.260  < 2e-16 ***
poly(DOY, 3)1 -7.189278   0.277769 -25.882  < 2e-16 ***
poly(DOY, 3)2 -1.507044   0.277769  -5.426 7.31e-08 ***
poly(DOY, 3)3  0.716330   0.277769   2.579   0.0101 *  
---
Signif. codes:  0 ‘***’ 0.001 ‘**’ 0.01 ‘*’ 0.05 ‘.’ 0.1 ‘ ’ 1

Residual standard error: 0.2778 on 964 degrees of freedom
Multiple R-squared:  0.4227,	Adjusted R-squared:  0.421 
F-statistic: 235.3 on 3 and 964 DF,  p-value: < 2.2e-16
```

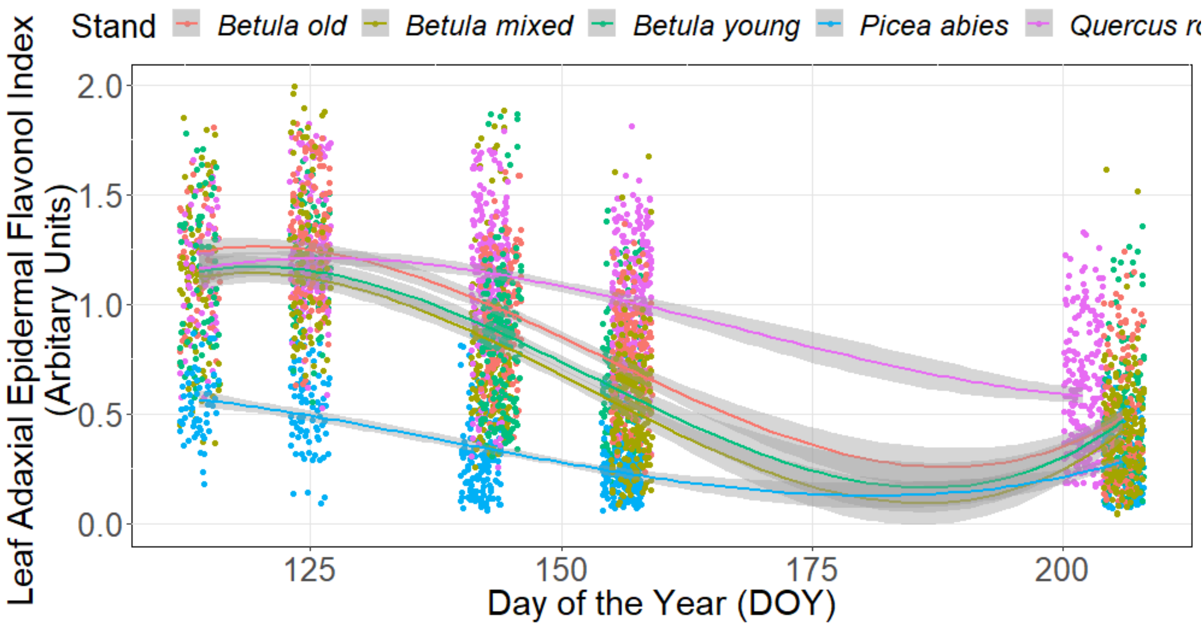

A2 Figure S1. A 3rd order polynomial function fitted to I_{flav} data from different stands in 2015.

To investigate whether temperature was an important driver of changes in adaxial I_{flav} during the consecutive years 2015-2016, we chose minimum daily air temperature out of the co-linear weather-related variables as explanatory variable in the model. This decision was based on high correlation with I_{flav} and expected response of I_{flav} to low temperature spells. Furthermore, we examined the relationship between minimum air temperature and abaxial I_{flav} separately for 2016.

We found additive models to give a better fit than linear regression models for both adaxial and abaxial I_{flav} (A2 Figure S2). The model selection was based on a combination of visual inspection (normality, outliers, residuals plotted against fitted values & residuals plotted against explanatory variables), comparison AIC/BIC and t -and F-statistics when suitable. Out of different models explaining changes in adaxial I_{flav}, we found the best model to be GAMM with correlations structure (R function corCompSymm) and variance structure (R function VarIdent) allowing for different residual spread in different stands (A2 Figure S3). However, for the abaxial I_{flav} models we did not find a satisfactory fit by simply adding a variance structure (R function VarComb structure to allow different spread over values of explanatory variables, as well as in different stands which did not converge) and/or correlation structure (R function corCompSymm or CorAr1) to GAMM. Instead we tested complicated GAMMs including global and/or group-level smoothers with differing smoothness with or without shared trend and/or interaction term. However, the resulting model had still low R^2-values, and low edf values, although model selection process and visual inspection indicated better fit for smoothing models (A2 Figure S4).

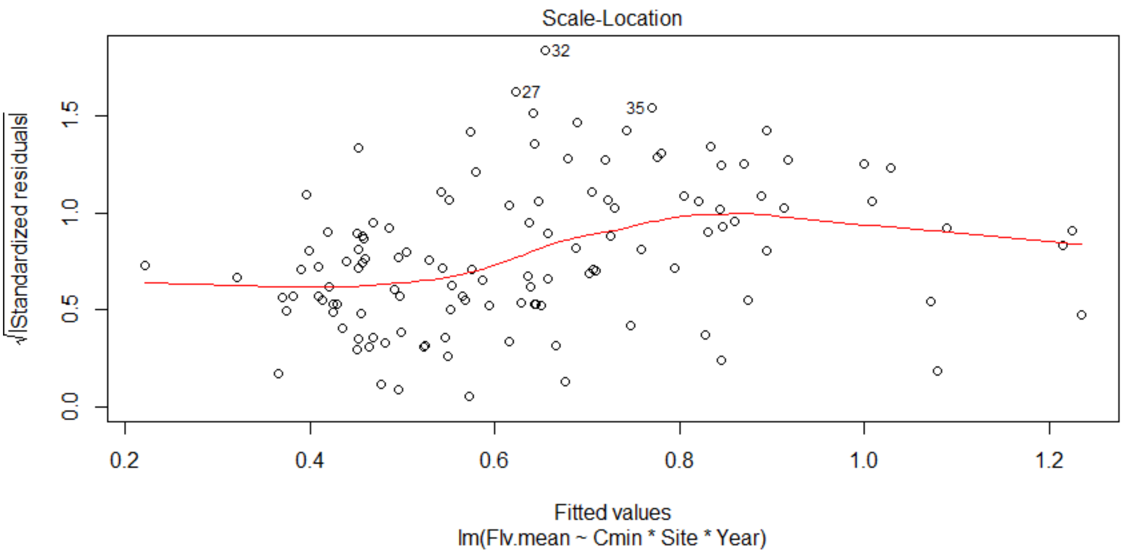

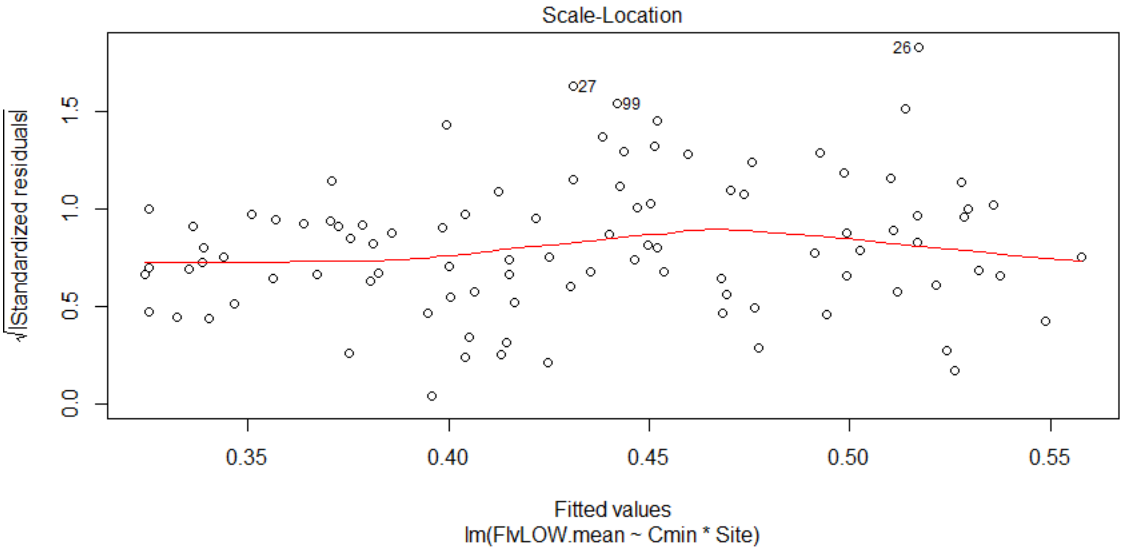

A2 Figure S2. Standardised residuals of linear regression model for adaxial (above) and abaxial (below) I_{flav} with minimum daily air temperature as explanatory variable, plotted against fitted values showing problems i.e. pattern (above) and differences in spread of residuals (below) related to the model.

The best models chosen in the model selection process:
Adaxial I_{flav}:

```
> anova(M0a2$gam)

Family: gaussian
Link function: identity

Formula:
Flv.mean ~ s(Cmin, bs = "cr") + Site

Parametric Terms:
     df     F  p-value
Site  4 9.922 6.68e-07

Approximate significance of smooth terms:
          edf Ref.df     F  p-value
s(Cmin) 1.881  1.881 18.17 1.71e-06
```

```
> summary(M0a2$gam)

Family: gaussian
Link function: identity

Formula:
Flv.mean ~ s(Cmin, bs = "cr") + Site

Parametric coefficients:
            Estimate Std. Error t value Pr(>|t|)
(Intercept)  0.69903    0.12979   5.386 4.08e-07 ***
SiteBeob    -0.01346    0.08132  -0.166   0.8688
SiteBey      0.07121    0.08379   0.850   0.3972
SitePic     -0.22757    0.10063  -2.261   0.0257 *
SiteQrob     0.09700    0.08019   1.210   0.2290
---
Signif. codes:  0 ‘***’ 0.001 ‘**’ 0.01 ‘*’ 0.05 ‘.’ 0.1 ‘ ’ 1

Approximate significance of smooth terms:
          edf Ref.df     F  p-value
s(Cmin) 1.881  1.881 18.17 1.71e-06 ***
---
Signif. codes:  0 ‘***’ 0.001 ‘**’ 0.01 ‘*’ 0.05 ‘.’ 0.1 ‘ ’ 1

R-sq.(adj) =  0.471
  Scale est. = 0.1127    n = 118
```

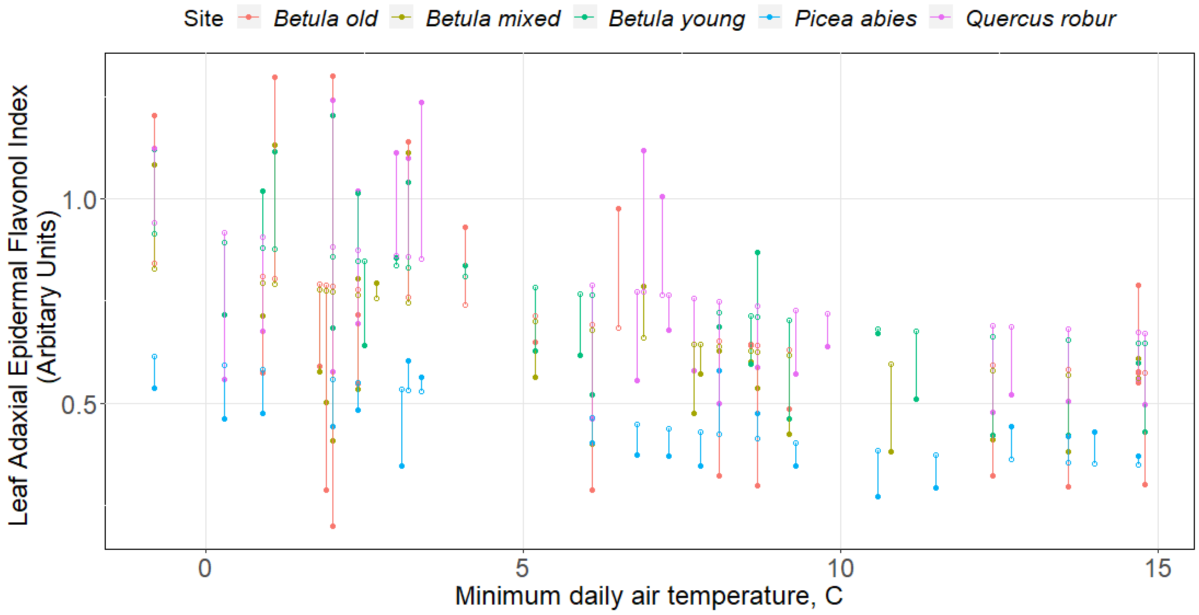

A2 Figure S3. Actual (filled circles) and predicted (empty circles) values from final model predicting changes in adaxial I_{flav} with minimum daily air temperature (C min) as explanatory variable.

Abaxial I_{flav}:

```
> M0$gam %>% summary()

Family: gaussian
Link function: identity

Formula:
FlvLOW.mean ~ s(Cmin, bs = "tp") + s(Site, k = 3, bs = "re")

Parametric coefficients:
            Estimate Std. Error t value Pr(>|t|)
(Intercept)  0.42611    0.02269   18.78   <2e-16 ***
---
```

```
Signif. codes:  0 ‘***’ 0.001 ‘**’ 0.01 ‘*’ 0.05 ‘.’ 0.1 ‘ ’ 1

Approximate significance of smooth terms:
          edf Ref.df     F  p-value    
s(Cmin) 1.069  1.069 10.97   0.0013 ** 
s(Site) 3.639  4.000 10.40 9.51e-08 ***
---
Signif. codes:  0 ‘***’ 0.001 ‘**’ 0.01 ‘*’ 0.05 ‘.’ 0.1 ‘ ’ 1

R-sq.(adj) =  0.328   
  Scale est. = 0.0030476  n = 97
```

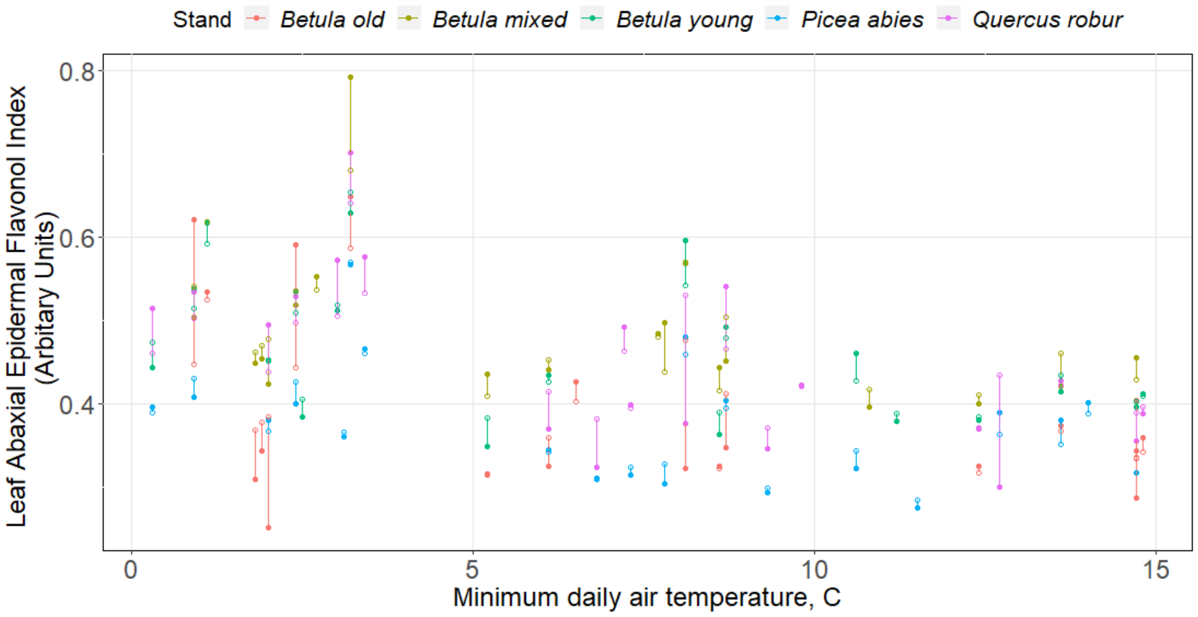

A2 Figure S4. Actual (filled circles) and predicted (empty circles) values from the final model for changes in abaxial I_{flav} with minimum daily air temperature as explanatory variable.

2. Comparison of optically measured I_{flav} and extracted flavonoids

In this section we tested the consistency of trends derived from the optical leaf-clip method i.e. I_{flav} compared with whole leaf flavonoid extracts. We used the mean absorbance of leaf extracts from UV region (UV-B, UV-A and combination of UV-B + UV-A), since we found a weak relationship between absorbance of leaf extracts at 375 nm and I_{flav}. All these regions were co-linear (A2 Figure S5) and fitting them all in the same model was not possible, hence we made separate models for each explanatory variable. The relationship in some measurements was non-linear and fitting a species-specific linear model showed clear patterns in visual inspection of residuals plotted against fitted values, indicating a violation of homogeneity. We fitted GLS with different variance structures (allowing for heterogeneity of residuals among different measurement times, but assuming homogeneity within one), which indicated a need for further extension of temporal correlation structure in some models. If species-specific GLS did not pass model selection process, we tried more complex models, namely GAMM. Nevertheless, some low edf values indicating a linear relationship were found in GAMM, but in most cases the relationship was not linear for all samples within a species (also see results section for possible explanation).

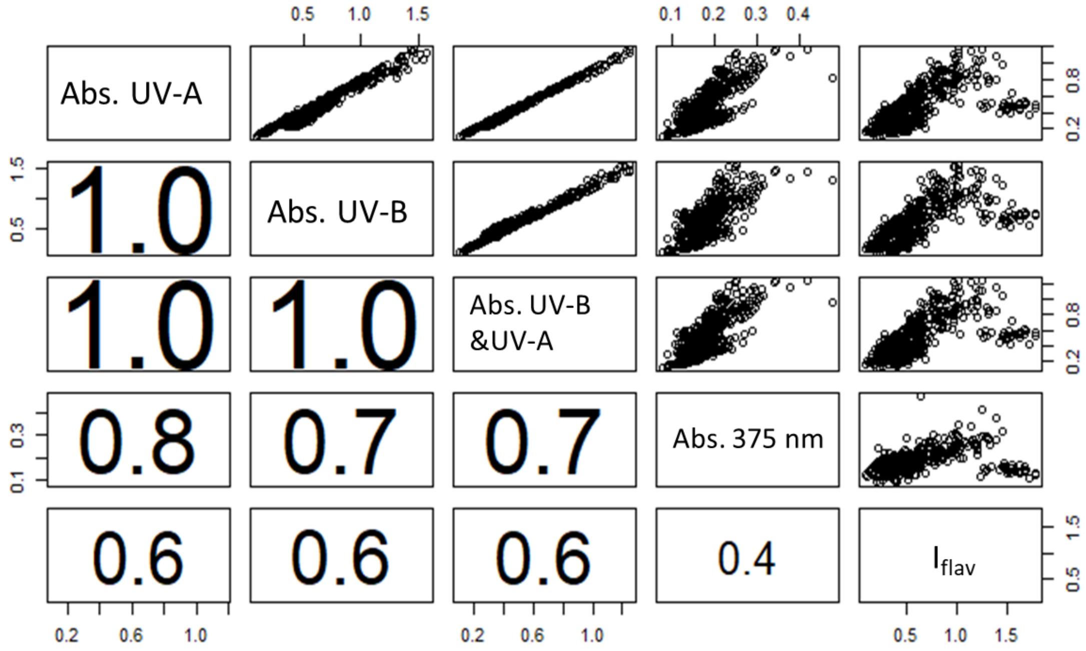

A2 Figure S5. Collinearity between explanatory variables: mean absorbance of leaf extracts calculated over different wavelength regions (UV-B, UV-A, combination of UV-B & UV-A) and absorbance at 375 nm. Final variable is the explanatory variable i.e. optically measured I_{flav}.

The best species-specific models chosen in the model selection process:

Aegopodium podagraria:
The linear regression model had clear problems when residuals were plotted against fitted values (A2 Figure S6). Although seemingly the extended GLS gave a good fit in this species, the predicted values were all negative. Since we kept iterating between finding a good model in visual inspection and did not find a solution within these models, we tried more complex GAMM. The selected model allowed smoothers to change over different measurement times (A2 Figure S7). Throughout the model selection process, models with mean absorbance over UV-B region as explanatory variable gave the best fit.

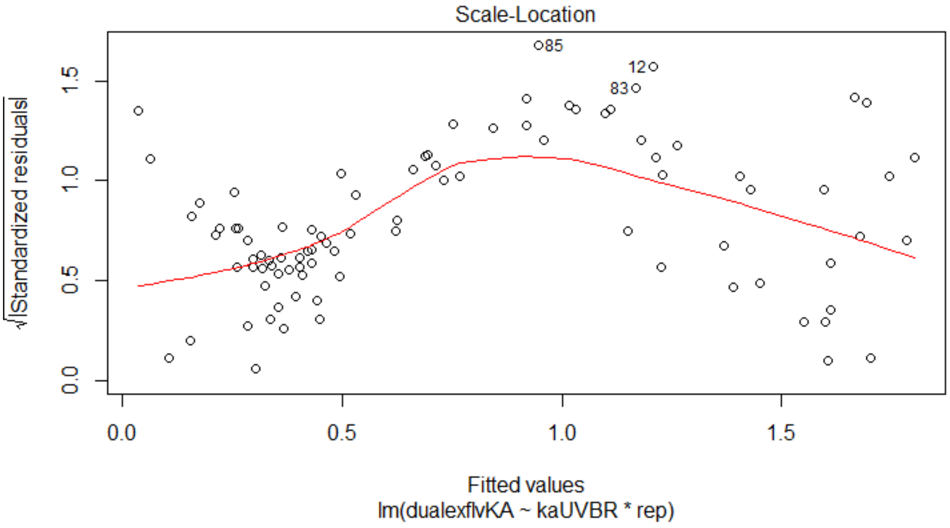

A2 Figure S6. Standardised residuals of the linear regression model plotted against fitted values (*A. podagraria*).

```
> anova(MG2ev2c$gam)

Family: gaussian
Link function: identity

Formula:
dualexflvKA ~ s(kaUVBR, by = REP, bs = "cr") + rep

Parametric Terms:
    df    F p-value
rep  1 1205  <2e-16

Approximate significance of smooth terms:
                 edf Ref.df       F  p-value
s(kaUVBR):REP2 1.000  1.000   7.544  0.00765
s(kaUVBR):REP3 7.488  7.488 107.416  < 2e-16
s(kaUVBR):REP4 3.032  3.032 258.394  < 2e-16
s(kaUVBR):REP5 5.453  5.453  50.290  < 2e-16
s(kaUVBR):REP6 2.746  2.746  33.759 3.86e-14

> summary(MG2ev2c$gam)

Family: gaussian
Link function: identity

Formula:
dualexflvKA ~ s(kaUVBR, by = REP, bs = "cr") + rep

Parametric coefficients:
             Estimate Std. Error t value Pr(>|t|)
(Intercept)  1.863440   0.037380   49.85   <2e-16 ***
rep         -0.247701   0.007134  -34.72   <2e-16 ***
---
Signif. codes:  0 ‘***’ 0.001 ‘**’ 0.01 ‘*’ 0.05 ‘.’ 0.1 ‘ ’ 1

Approximate significance of smooth terms:
                 edf Ref.df       F  p-value
s(kaUVBR):REP2 1.000  1.000   7.544  0.00765 **
s(kaUVBR):REP3 7.488  7.488 107.416  < 2e-16 ***
s(kaUVBR):REP4 3.032  3.032 258.394  < 2e-16 ***
s(kaUVBR):REP5 5.453  5.453  50.290  < 2e-16 ***
s(kaUVBR):REP6 2.746  2.746  33.759 3.86e-14 ***
---
Signif. codes:  0 ‘***’ 0.001 ‘**’ 0.01 ‘*’ 0.05 ‘.’ 0.1 ‘ ’ 1

R-sq.(adj) =   0.95
  Scale est. = 0.023886  n = 90
```

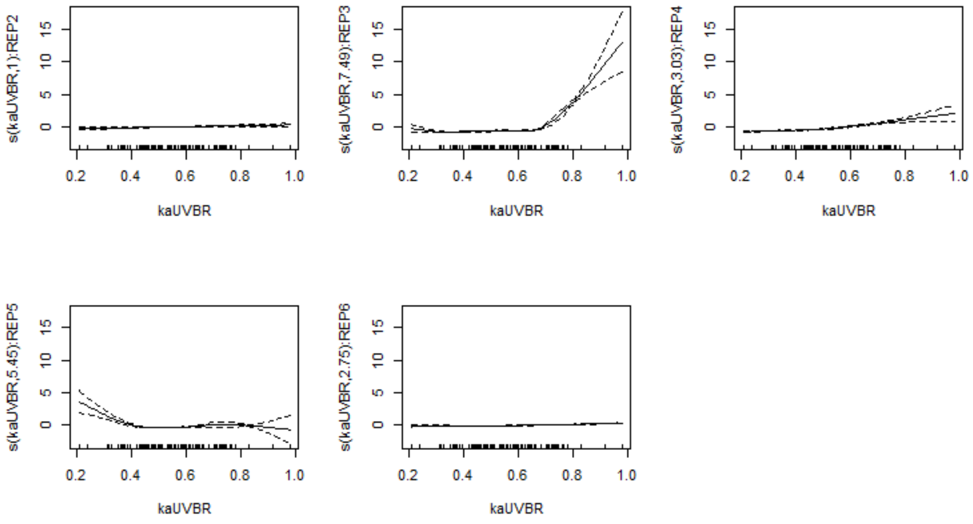

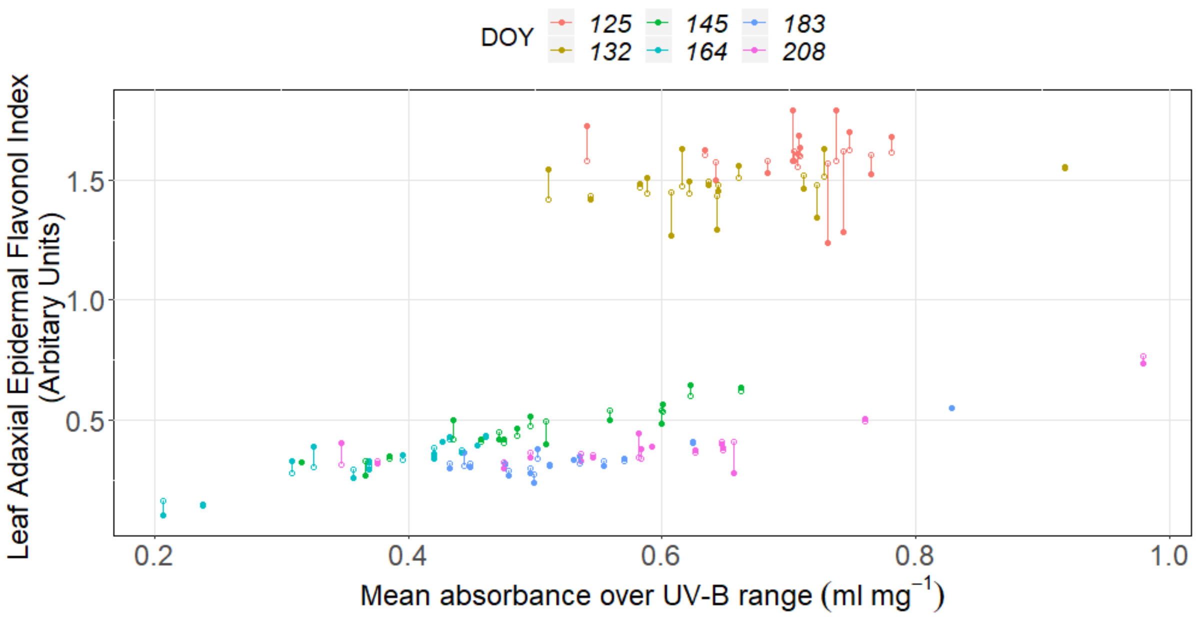

A2 Figure S7. Actual (filled circles) and predicted (empty circles) values from the final model of *A. podagraria* for changes in adaxial I_{flav} with mean absorbance over UV-B region as explanatory variable.

Anemone nemorosa:

Both the linear regression model and the extended GLS showed clear problems in visual inspection of the residuals plotted against fitted values (A2 Figure S8). Tests indicated a better fit with smoothing models over linear models. Throughout the model selection process, the best GAMMs had absorbance over UV-A region or some over UV-B and UV-A region combined as explanatory variable (A2 Figure S9). Compared to the other species, a variance structure allowing for different spread of residuals over the values of explanatory variable and in different stands was better than a variance structure allowing for different spread over different measurement times.

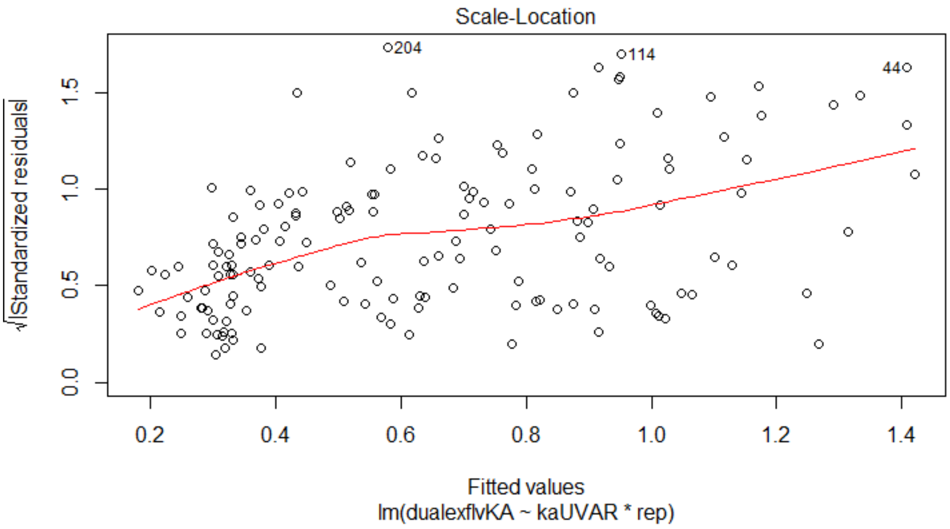

A2 Figure S8. Standardised residuals of linear regression model plotted against fitted values showing increase in residual spread (*A. nemorosa*).

```
> anova(AnM0f$gam)

Family: gaussian
Link function: identity

Formula:
dualexflvKA ~ s(kaUVAR, bs = "cr") + rep + stand

Parametric Terms:
      df     F  p-value
rep    1 36.91 1.04e-08
stand  1 51.32 3.69e-11

Approximate significance of smooth terms:
            edf Ref.df     F p-value
s(kaUVAR) 2.389  2.389 50.45  <2e-16

> summary(AnM0f$gam)

Family: gaussian
Link function: identity

Formula:
dualexflvKA ~ s(kaUVAR, bs = "cr") + rep + stand

Parametric coefficients:
                    Estimate Std. Error t value Pr(>|t|)
(Intercept)         0.599384   0.021986  27.262  < 2e-16 ***
rep                -0.031367   0.005163  -6.075 1.04e-08 ***
standQuercus robur  0.285287   0.039825   7.164 3.69e-11 ***
---
Signif. codes:  0 ‘***’ 0.001 ‘**’ 0.01 ‘*’ 0.05 ‘.’ 0.1 ‘ ’ 1

Approximate significance of smooth terms:
            edf Ref.df     F p-value
s(kaUVAR) 2.389  2.389 50.45  <2e-16 ***
---
Signif. codes:  0 ‘***’ 0.001 ‘**’ 0.01 ‘*’ 0.05 ‘.’ 0.1 ‘ ’ 1

R-sq.(adj) =  0.875
  Scale est. = 0.028652  n = 150
```

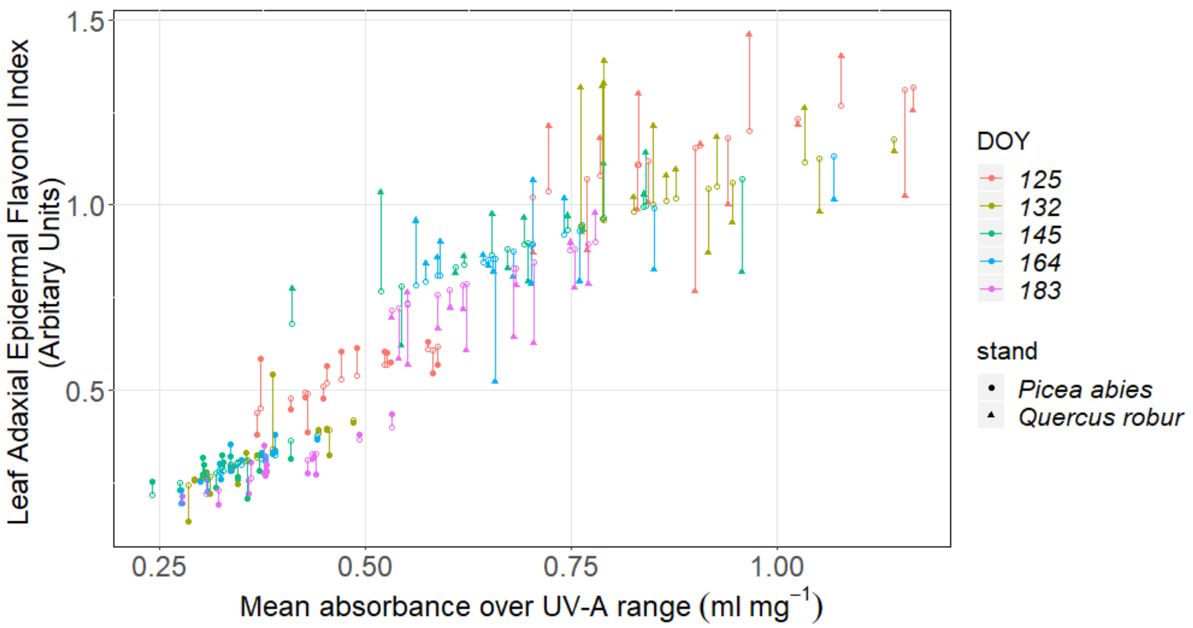

A2 Figure S9. Actual (filled circles) and predicted (empty circles) values from the final model of *A. nemorosa* for changes in adaxial I_{flav} with mean absorbance over UV-A region as explanatory variable.

Convallaria majalis:
The linear regression model showed clear problems in visual inspection (A2 Figure S10). Furthermore, all linear regressions had particularly low R^2-values in this species, indicating a poor fit of the model. GLS with extensions taking into account heterogeneity, did not improve model performance, thus we moved on to using GAMM. Throughout the model selection process, the models with UV-B region or with UV-B and UV-A region combined as explanatory variables were found best suited for *C. majalis* data (A2 Figure S11). The selected model in this species was not as good as for other species, which might have been related to lower I_{flav} values in general.

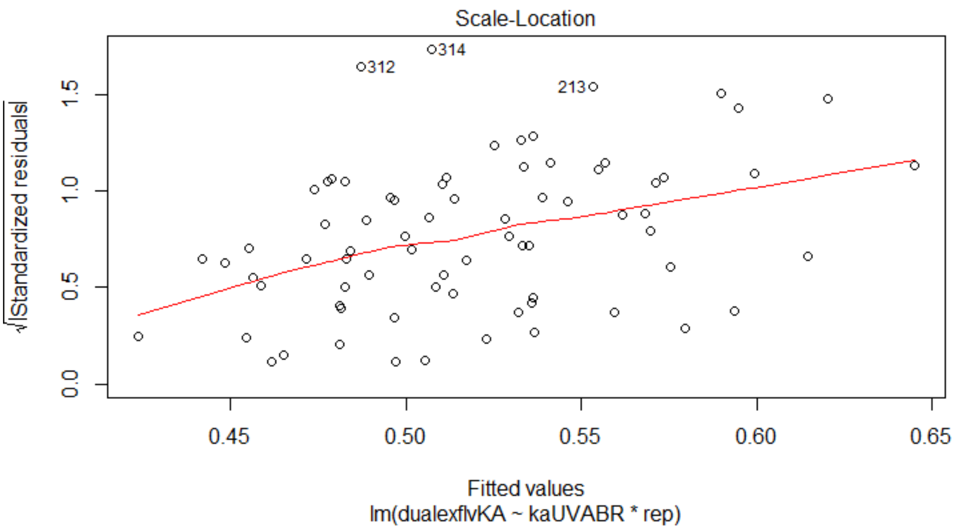

A2 Figure S10. Standardised residuals of the linear regression model plotted against fitted values showing clear problems related to the model after exclusion of obvious outliers (*C. majalis*).

```
> anova(CoM0b$gam)
```

```
Family: gaussian
Link function: identity

Formula:
dualexflvKA ~ s(kaUVBR, by = rep, bs = "cr")

Approximate significance of smooth terms:
              edf Ref.df     F  p-value
s(kaUVBR):rep   2      2 24.53 3.61e-09
> summary(CoMOb$gam)

Family: gaussian
Link function: identity

Formula:
dualexflvKA ~ s(kaUVBR, by = rep, bs = "cr")

Parametric coefficients:
            Estimate Std. Error t value Pr(>|t|)
(Intercept)  0.56620    0.01947   29.07   <2e-16 ***
---
Signif. codes:  0 ‘***’ 0.001 ‘**’ 0.01 ‘*’ 0.05 ‘.’ 0.1 ‘ ’ 1

Approximate significance of smooth terms:
              edf Ref.df     F  p-value
s(kaUVBR):rep   2      2 24.53 3.61e-09 ***
---
Signif. codes:  0 ‘***’ 0.001 ‘**’ 0.01 ‘*’ 0.05 ‘.’ 0.1 ‘ ’ 1

R-sq.(adj) =  0.393
  Scale est. = 0.0032233  n = 74
```

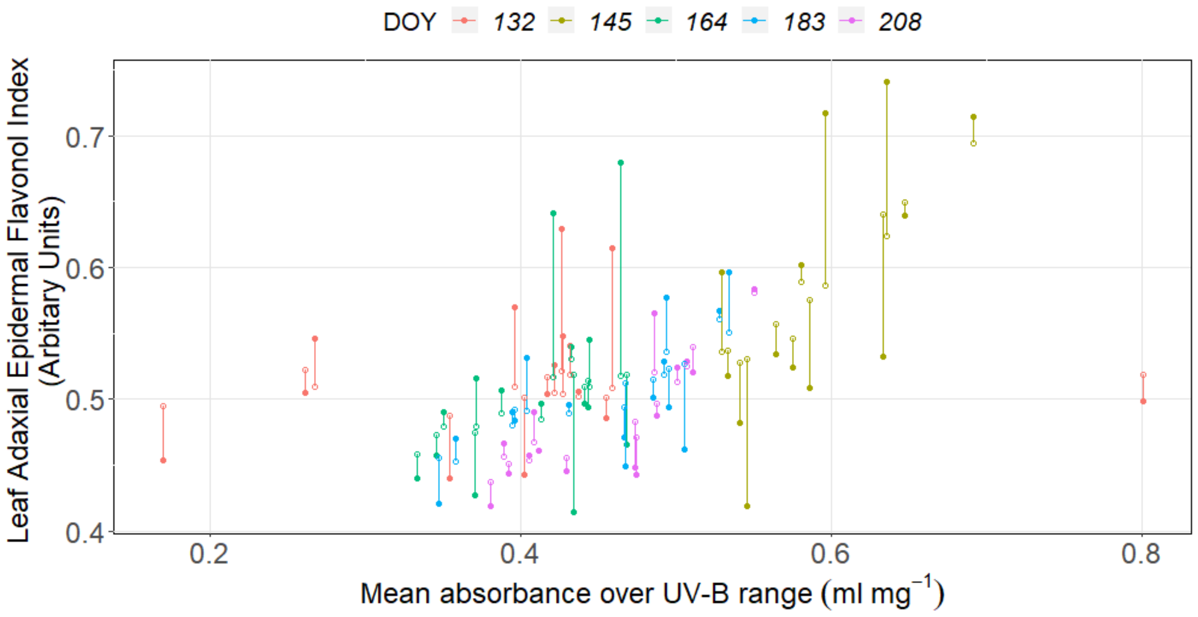

A2 Figure S11. Actual (filled circles) and predicted (empty circles) values from the final model of *C. majalis* for changes in adaxial I_{flav} with mean absorbance over UV-A region as explanatory variable.

Hepatica nobilis:
The linear regression model showed problems in the model with visual inspection (A2 Figure S12). However, the best model was found with GLS including a variance structure allowing

for different residual spread over UV-A region and for different measurement times. Throughout, best models for *H. nobilis* were found with UV-A region as explanatory variable (A2 Figure S13).

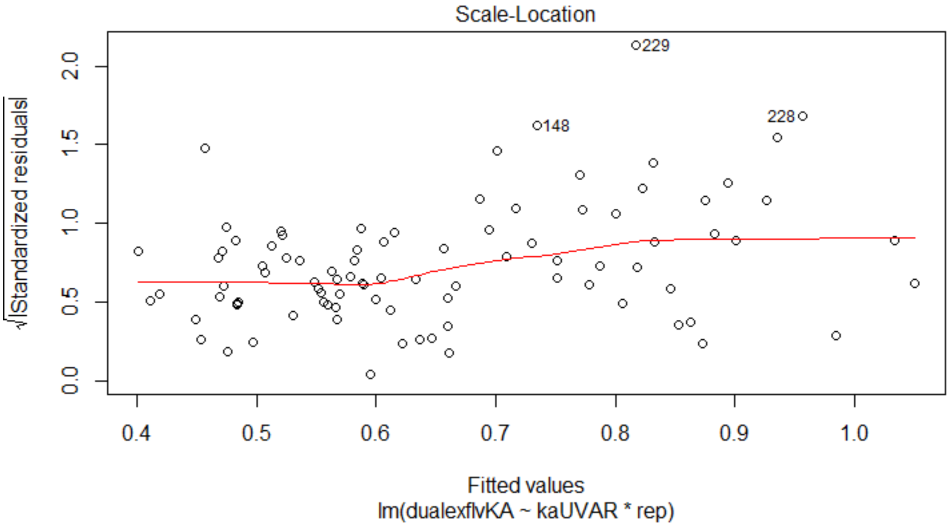

A2 Figure S12. Standardised residuals of linear regression model plotted against fitted values (*H. nobilis*).

```
> anova(HeM0d)
Denom. DF: 81
            numDF  F-value p-value
(Intercept)     1 9290.398  <.0001
kaUVAR          1  307.125  <.0001
rep             5    8.640  <.0001

> summary(HeM0d)
Generalized least squares fit by REML
  Model: dualexflvKA ~ kaUVAR + rep
  Data: md
        AIC       BIC   logLik
  -161.7291 -130.6012 93.86453

Combination of variance functions:
 Structure: Different standard deviations per stratum
 Formula: ~1 | rep
 Parameter estimates:
        1         2         3         4         5         6
1.0000000 0.9876570 1.9610512 0.8534609 0.4856048 0.7890333
Variance function:
 Structure: fixed weights
 Formula: ~kaUVAR

Coefficients:
                 Value  Std.Error   t-value p-value
(Intercept)  0.2049816 0.05012602  4.089324  0.0001
kaUVAR       0.7488770 0.05501857 13.611351  0.0000
rep2        -0.0269363 0.03006979 -0.895792  0.3730
rep3        -0.0769696 0.04530713 -1.698841  0.0932
rep4        -0.1120020 0.02990409 -3.745375  0.0003
rep5        -0.1158037 0.02495876 -4.639803  0.0000
rep6        -0.1344136 0.02803492 -4.794506  0.0000

 Correlation:
       (Intr) kaUVAR rep2   rep3   rep4   rep5
kaUVAR -0.905
rep2   -0.622  0.355
rep3   -0.359  0.176  0.395
rep4   -0.728  0.471  0.670  0.417
rep5   -0.697  0.370  0.735  0.465  0.781
```

```
rep6   -0.675  0.390  0.675  0.425  0.723  0.791

Standardized residuals:
       Min         Q1        Med         Q3        Max 
-1.9499090 -0.6905911 -0.1036591  0.4043757  2.9875873 

Residual standard error: 0.09070943 
Degrees of freedom: 88 total; 81 residual
```

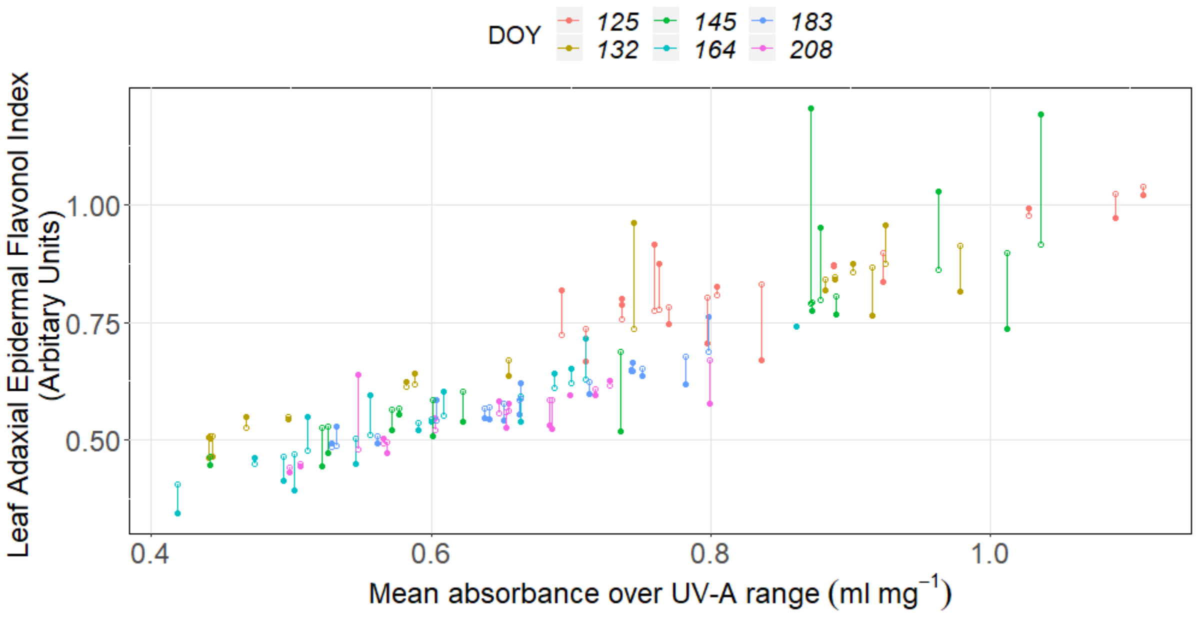

A2 Figure S13. Actual (filled circles) and predicted (empty circles) values from the final model of *H. nobilis* for changes in adaxial I_{flav} with mean absorbance over UV-A region as explanatory variable.

Oxalis acetosella:

The linear regression model showed patterns in visual inspection (A2 Figure S14) and GLS extended with different variance structures did not fully fix problems related to different spreads over different UV regions. Smoothing models indicated a better fit and thus we used GAMM (although 3 measurement times had edf values of 1). For some reason linear regression models and GLS gave a better fit with models using absorbance over UV-B and UV-A region as explanatory variable, but smoothing models using absorbance over UV-B as explanatory variable were considered best (A2 Figure S15). Visual inspection of the final model was adequate.

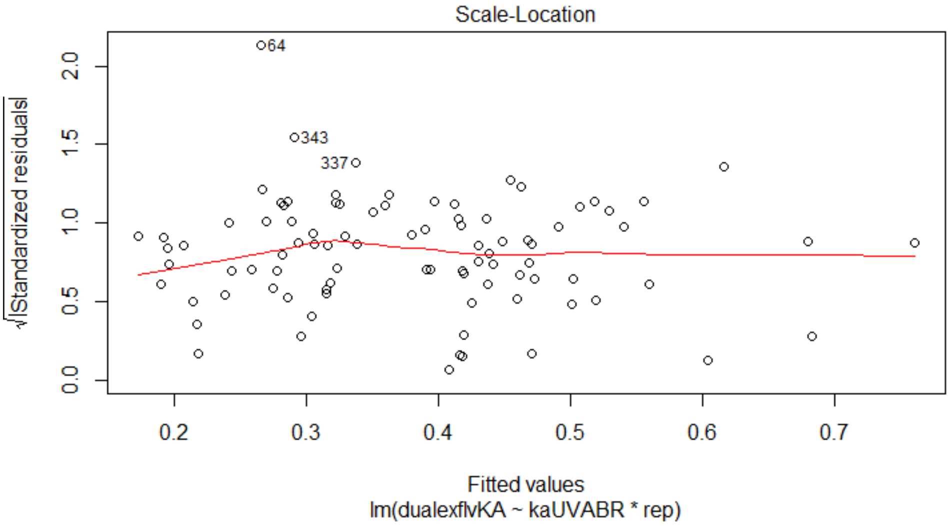

A2 Figure S14. Standardised residuals of linear regression model plotted against fitted values showing some problems related to the model (*O. acetosella*).

```
> anova(OxM0a$gam)

Family: gaussian
Link function: identity

Formula:
dualexflvKA ~ s(kaUVBR, by = REP, bs = "cr") + rep

Parametric Terms:
    df     F  p-value
rep  1 16.75 0.000107

Approximate significance of smooth terms:
                 edf Ref.df      F  p-value
s(kaUVBR):REP2 3.963  3.963 94.801  < 2e-16
s(kaUVBR):REP3 5.354  5.354 61.625  < 2e-16
s(kaUVBR):REP4 1.000  1.000 37.339 2.85e-08
s(kaUVBR):REP5 1.000  1.000  6.693   0.0116
s(kaUVBR):REP6 1.000  1.000 22.844 7.90e-06

> summary(OxM0a$gam)

Family: gaussian
Link function: identity

Formula:
dualexflvKA ~ s(kaUVBR, by = REP, bs = "cr") + rep

Parametric coefficients:
             Estimate Std. Error t value Pr(>|t|)
(Intercept)  0.596800   0.029827  20.009  < 2e-16 ***
rep         -0.024271   0.005931  -4.093 0.000107 ***
---
Signif. codes:  0 ‘***’ 0.001 ‘**’ 0.01 ‘*’ 0.05 ‘.’ 0.1 ‘ ’ 1

Approximate significance of smooth terms:
                 edf Ref.df      F  p-value
s(kaUVBR):REP2 3.963  3.963 94.801  < 2e-16 ***
s(kaUVBR):REP3 5.354  5.354 61.625  < 2e-16 ***
s(kaUVBR):REP4 1.000  1.000 37.339 2.85e-08 ***
s(kaUVBR):REP5 1.000  1.000  6.693   0.0116 *
s(kaUVBR):REP6 1.000  1.000 22.844 7.90e-06 ***
---
Signif. codes:  0 ‘***’ 0.001 ‘**’ 0.01 ‘*’ 0.05 ‘.’ 0.1 ‘ ’ 1
```

```
R-sq.(adj) =  0.886
  Scale est. = 0.0091049  n = 89
```

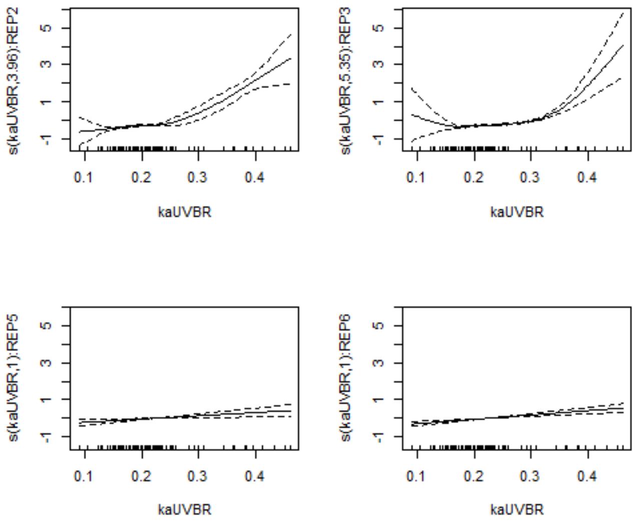

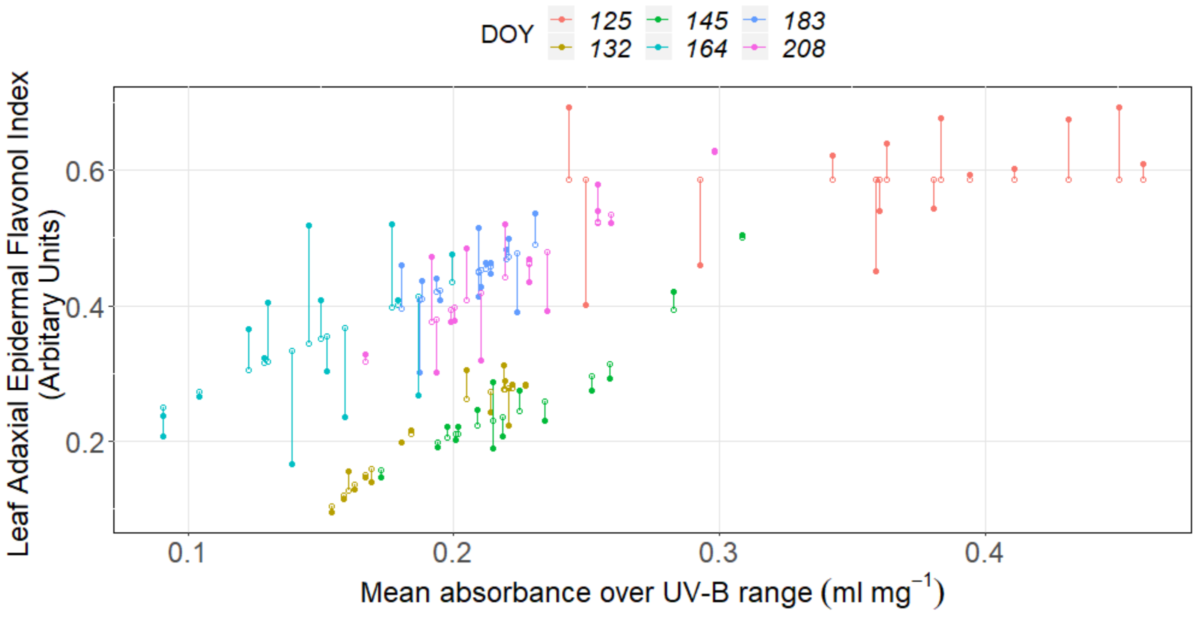

A2 Figure S15. Actual (filled circles) and predicted (empty circles) values from the final model of *O. acetosella* for changes in adaxial I_{flav} with mean absorbance over UV-B region as explanatory variable.

3. Relating understorey I_{flav} and understorey spectral irradiance

In this section we compared the obtained I_{flav} values with corresponding spectral irradiance measurements. However, a direct comparison of these data was not possible, since our observational unit was different for I_{flav} measurements (plant individual) compared to understorey irradiance measurements (measurement point). Instead we used the mean I_{flav} calculated for each measurement point to match our observational units. We considered this approach to be adequate for our objectives to test whether any spectral component aligned well with the coarser changes observed with I_{flav}. However, this approach unfortunately

excluded for instance the possibility to investigate whether different species had more sensitivity for any spectral components. Since these data were compared with the mean I_{flav} from the same measurement point, all different understorey positions i.e. shade, sunfleck or position where radiation was transmitted through canopy i.e. *leaf* position, were compared with the same mean I_{flav}. Including all understorey positions into the same model was not functional, since excluding any of these positions if found non-significant would not be possible. Hence, we made separate models for spectral irradiance from each understorey position to investigate which of these understorey positions (or rather the spectral irradiance) seemed to best explain changes in I_{flav}.

The linear regression models made for different spectral components as explanatory variables (A2 Figure S16), either with or without variable stand in the model showed unwanted patterns in visual inspection of the residuals plotted against fitted values. Furthermore, some models also showed patterns when residuals were plotted against explanatory variables.

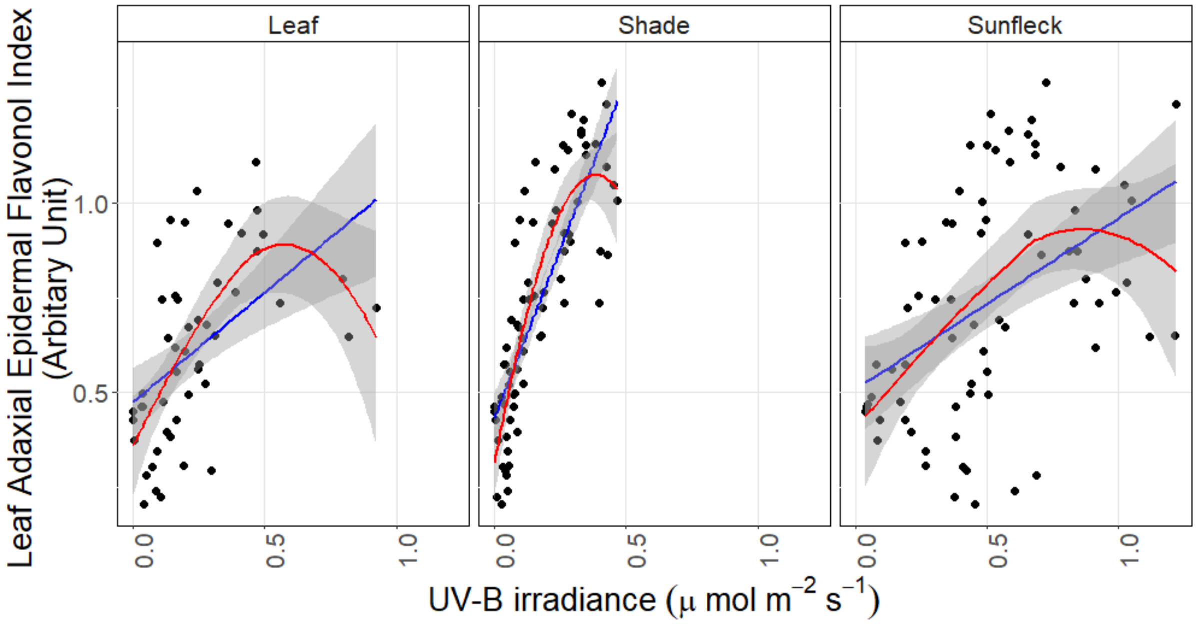

A2 Figure S16. Relationship between mean I_{flav} and unweighted UV-B irradiance measured in different understorey positions with linear regression trendline (blue) and with loess fit (red). The grey band is 95% confidence interval. Adding stands as variable to the models improved the model fit.

Understorey shade:
The selected model for spectral irradiance measured in understorey shade had effective UV dose calculated according to biological spectral weighting function (BSWF) for plant growth (PG, Flint & Caldwell 2003) as explanatory variable. However, the model with UV-A irradiance as explanatory variable was almost similar based on AIC/BIC values. Furthermore, throughout the model selection process best models had PG or UV-A as explanatory variable (A2 Figure S17). However, the difference between these selected models and models using other spectral regions (e.g. PAR) as explanatory variable was not indisputable (R^2-values: 0.86 vs 0.80). The selected model with spectral irradiance from understorey shade as explanatory variable was better in terms of AIC/BIC, R^2-value and visual inspection than the models with spectral irradiance from other understorey positions as explanatory variable.

```
              df       AIC
MG5c$lme      13 -81.47300      #shade with PG(q)
MG5cv2$lme    17 -80.68912      #shade with PG(q)
MG2cv2$lme    17 -78.57355      #shade with UV-A(q)
MG8ev2$lme    19 -14.70789      #sun with UV-A(q)
MG11ev2$lme   19 -14.62560      #sun with PG(q)
MG18bv2c$lme  14 -48.95157      #leaf with GEN(G)(q)
MG18cv2c$lme  14 -48.95157      #leaf with GEN(G)(q)
MG16bv2$lme   13 -43.55993      #leaf with FLAV(q)
```

```
> anova(ShM0$gam)

Family: gaussian
Link function: identity

Formula:
FlvWMean ~ s(PG_q, by = Stand, bs = "cr")

Approximate significance of smooth terms:
                              edf Ref.df     F  p-value
s(PG_q):StandBetula mixed   1.799  1.799 53.58 3.11e-13
s(PG_q):StandBetula old     1.000  1.000 98.24  < 2e-16
s(PG_q):StandBetula young   3.277  3.277 11.94 9.20e-07
s(PG_q):StandPicea abies    1.000  1.000 29.87 7.06e-07
s(PG_q):StandQuercus robur 3.560  3.560 20.22 7.23e-11

> summary(ShM0$gam)

Family: gaussian
Link function: identity

Formula:
FlvWMean ~ s(PG_q, by = Stand, bs = "cr")

Parametric coefficients:
            Estimate Std. Error t value Pr(>|t|)
(Intercept)  0.77057    0.03553   21.69   <2e-16 ***
---
Signif. codes:  0 ‘***’ 0.001 ‘**’ 0.01 ‘*’ 0.05 ‘.’ 0.1 ‘ ’ 1

Approximate significance of smooth terms:
                              edf Ref.df     F  p-value
s(PG_q):StandBetula mixed   1.799  1.799 53.58 3.11e-13 ***
s(PG_q):StandBetula old     1.000  1.000 98.24  < 2e-16 ***
s(PG_q):StandBetula young   3.277  3.277 11.94 9.20e-07 ***
s(PG_q):StandPicea abies    1.000  1.000 29.87 7.06e-07 ***
s(PG_q):StandQuercus robur 3.560  3.560 20.22 7.23e-11 ***
---
Signif. codes:  0 ‘***’ 0.001 ‘**’ 0.01 ‘*’ 0.05 ‘.’ 0.1 ‘ ’ 1

R-sq.(adj) =  0.857
  Scale est. = 0.010762  n = 73
```

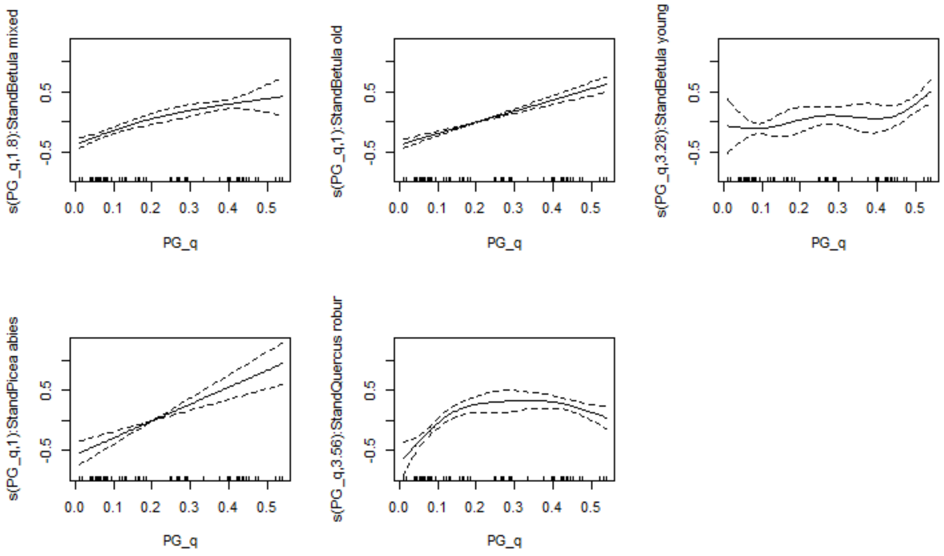

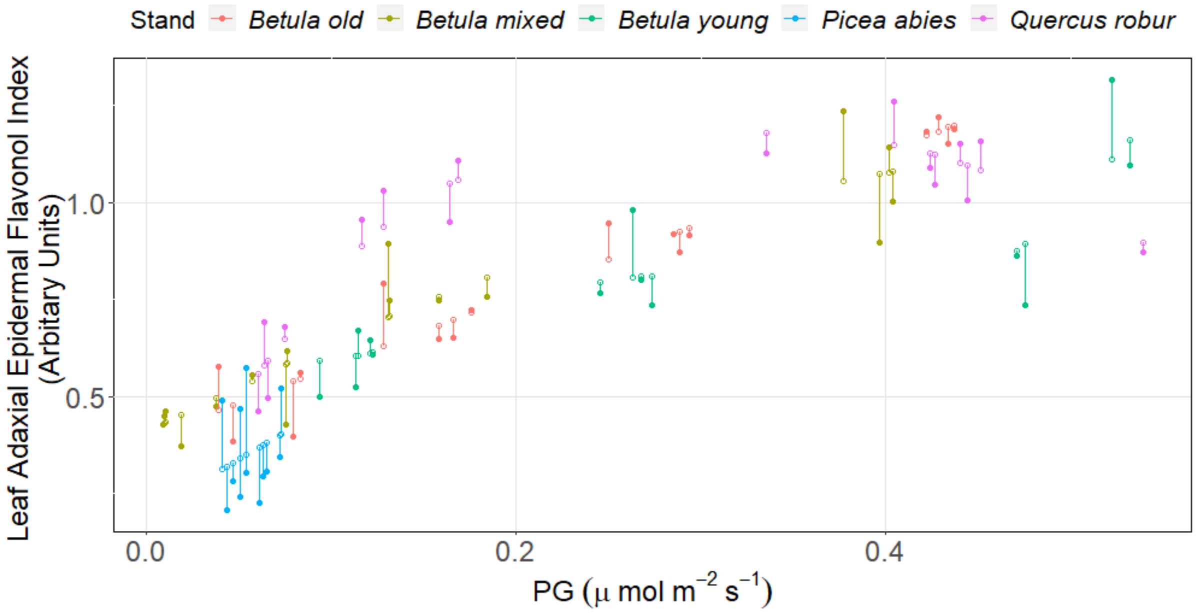

A2 Figure S17. Actual (filled circles) and predicted (empty circles) values from the final model for changes in mean adaxial I_{flav} with effective UV dose calculated according to BSWF for plant growth (PG) as explanatory variable. Spectral irradiance is measured from understorey shade.

Similar model for understorey shade with unweighted UV-A irradiation as explanatory variable:

```
> anova(ShM0$gam)

Family: gaussian
Link function: identity

Formula:
FlvWMean ~ s(UVA_q, by = Stand, bs = "cr")

Approximate significance of smooth terms:
                              edf Ref.df     F  p-value
s(UVA_q):StandBetula old    1.000  1.000 97.76  < 2e-16
```

```
s(UVA_q):StandBetula mixed  1.858   1.858 51.61 2.23e-13
s(UVA_q):StandBetula young  3.018   3.018 14.18 2.60e-07
s(UVA_q):StandPicea abies   1.000   1.000 25.09 4.28e-06
s(UVA_q):StandQuercus robur 3.662   3.662 19.81 1.58e-10
> summary(ShM0$gam)

Family: gaussian 
Link function: identity 

Formula:
FlvWMean ~ s(UVA_q, by = Stand, bs = "cr")

Parametric coefficients:
            Estimate Std. Error t value Pr(>|t|)    
(Intercept)  0.76902    0.03763   20.44   <2e-16 ***
---
Signif. codes:  0 '***' 0.001 '**' 0.01 '*' 0.05 '.' 0.1 ' ' 1

Approximate significance of smooth terms:
                              edf Ref.df     F  p-value    
s(UVA_q):StandBetula old    1.000  1.000 97.76  < 2e-16 ***
s(UVA_q):StandBetula mixed  1.858  1.858 51.61 2.23e-13 ***
s(UVA_q):StandBetula young  3.018  3.018 14.18 2.60e-07 ***
s(UVA_q):StandPicea abies   1.000  1.000 25.09 4.28e-06 ***
s(UVA_q):StandQuercus robur 3.662  3.662 19.81 1.58e-10 ***
---
Signif. codes:  0 '***' 0.001 '**' 0.01 '*' 0.05 '.' 0.1 ' ' 1

R-sq.(adj) =   0.85   
  Scale est. = 0.010834  n = 73
```

Understorey sunflecks:
The selected model for spectral irradiance measured in understorey sunflecks had UV-A irradiation as explanatory variable (A2 Figure S18). Throughout the model selection process, the best models had UV-A irradiation or effective UV dose calculated according to BSWF for plant growth (PG, Flint & Caldwell, 2003) as explanatory variable. However, as seen from A2 Figure S18, the model predicts opposite trend for the *Picea abies* stand compared to all the other stands, whereby highest UV-A irradiance results in lowest I_{flav} values which makes the model biologically less reliable and reduces its explanatory value.

```
> anova(SuM0c$gam)

Family: gaussian 
Link function: identity 

Formula:
FlvWMean ~ s(UVA_q, by = STAND, bs = "cr") + Stand

Parametric Terms:
      df     F p-value
Stand  4 2.674  0.0407

Approximate significance of smooth terms:
                              edf Ref.df      F  p-value
s(UVA_q):STANDBetula mixed  2.734  2.734  7.313 0.001328
s(UVA_q):STANDBetula young  1.827  1.827  1.341 0.238057
s(UVA_q):STANDPicea abies   2.154  2.154 22.891 1.05e-08
s(UVA_q):STANDQuercus robur 2.174  2.174  7.542 0.000878
> summary(SuM0c$gam)

Family: gaussian 
Link function: identity 

Formula:
FlvWMean ~ s(UVA_q, by = STAND, bs = "cr") + Stand
```

```
Parametric coefficients:
                      Estimate Std. Error t value Pr(>|t|)    
(Intercept)            0.75482    0.15064   5.011 5.38e-06 ***
StandBetula old        0.06550    0.20957   0.313   0.7557    
StandBetula young     -0.11357    0.24486  -0.464   0.6445    
StandPicea abies      -0.46743    0.19901  -2.349   0.0223 *  
StandQuercus robur     0.05072    0.21200   0.239   0.8118    
---
Signif. codes:  0 ‘***’ 0.001 ‘**’ 0.01 ‘*’ 0.05 ‘.’ 0.1 ‘ ’ 1

Approximate significance of smooth terms:
                                edf Ref.df      F  p-value    
s(UVA_q):STANDBetula mixed    2.734  2.734  7.313 0.001328 ** 
s(UVA_q):STANDBetula young    1.827  1.827  1.341 0.238057    
s(UVA_q):STANDPicea abies     2.154  2.154 22.891 1.05e-08 ***
s(UVA_q):STANDQuercus robur 2.174  2.174  7.542 0.000878 ***
---
Signif. codes:  0 ‘***’ 0.001 ‘**’ 0.01 ‘*’ 0.05 ‘.’ 0.1 ‘ ’ 1

R-sq.(adj) =  0.799
  Scale est. = 0.033364  n = 72
```

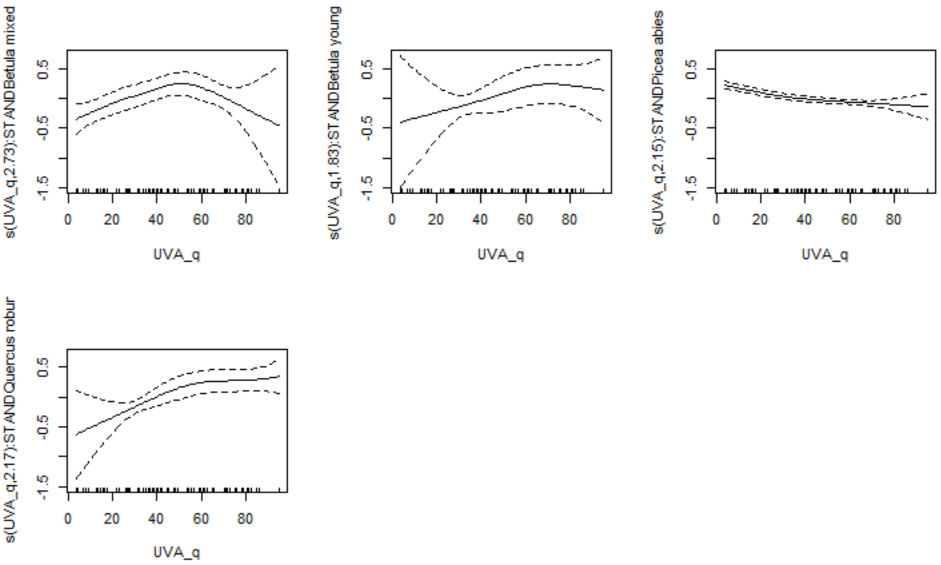

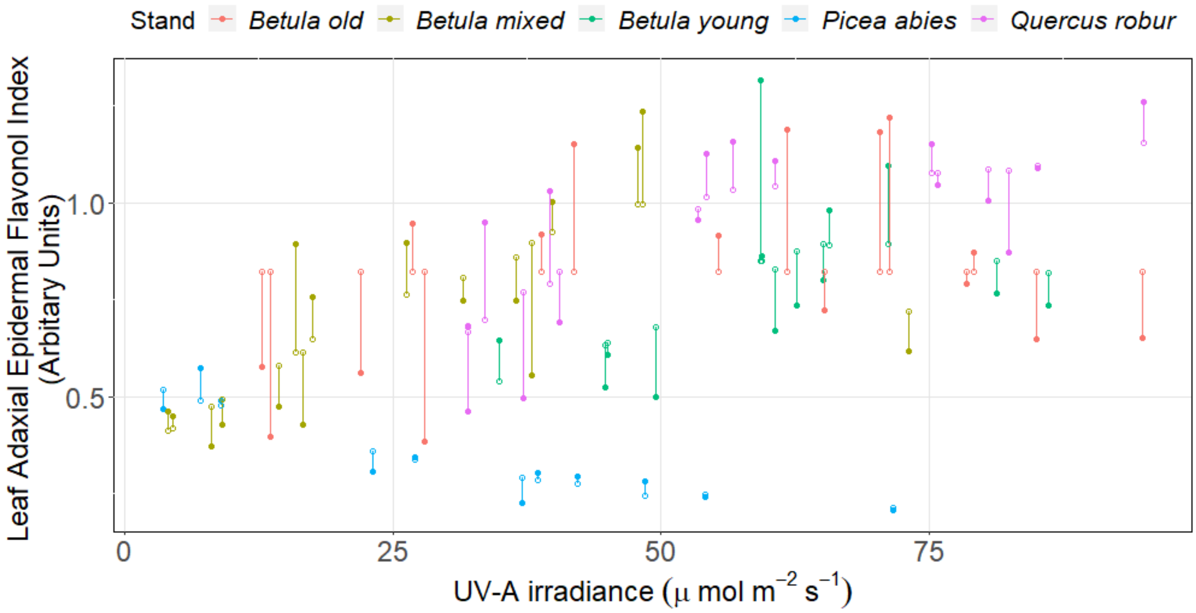

A2 Figure S18. Actual (filled circles) and predicted (empty circles) values from the final model for changes in mean adaxial I_{flav} with unweighted UV-A as explanatory variable. Spectral irradiance is measured from understorey sunflecks.

Understorey *leaf* position:

The selected model for spectral irradiance measured in understorey *leaf* position had effective UV dose calculated according to BSWF for flavonoid accumulation (FLAV, Ibdah et al., 2002) as explanatory variable (A2 Figure S19). However, the final model seemed to explain changes in mean I_{flav} poorly compared to spectral irradiance from other understorey positions. The selected model showed some problems in visual inspection of residuals plotted against fitted values, but we could not further improve the model by adding different variance structures.

```
> anova(LeM0$gam)

Family: gaussian
Link function: identity

Formula:
FlvWMean ~ s(FLAV_q, bs = "cr") + Stand

Parametric Terms:
      df     F p-value
Stand  4 1.534   0.211

Approximate significance of smooth terms:
          edf Ref.df     F  p-value
s(FLAV_q)   1      1 33.14 6.55e-07

> summary(LeM0$gam)

Family: gaussian
Link function: identity

Formula:
FlvWMean ~ s(FLAV_q, bs = "cr") + Stand

Parametric coefficients:
                    Estimate Std. Error t value Pr(>|t|)
(Intercept)         0.594128   0.128221   4.634 3.77e-05 ***
StandBetula mixed   0.095459   0.182560   0.523    0.604
```

```
StandBetula young    0.001803   0.180744   0.010    0.992
StandPicea abies    -0.246314   0.183350  -1.343    0.187
StandQuercus robur   0.178318   0.181278   0.984    0.331
---
Signif. codes:  0 '***' 0.001 '**' 0.01 '*' 0.05 '.' 0.1 ' ' 1

Approximate significance of smooth terms:
          edf Ref.df     F  p-value
s(FLAV_q)   1      1 33.14 6.55e-07 ***
---
Signif. codes:  0 '***' 0.001 '**' 0.01 '*' 0.05 '.' 0.1 ' ' 1

R-sq.(adj) =  0.687
  Scale est. = 0.018422  n = 46
```

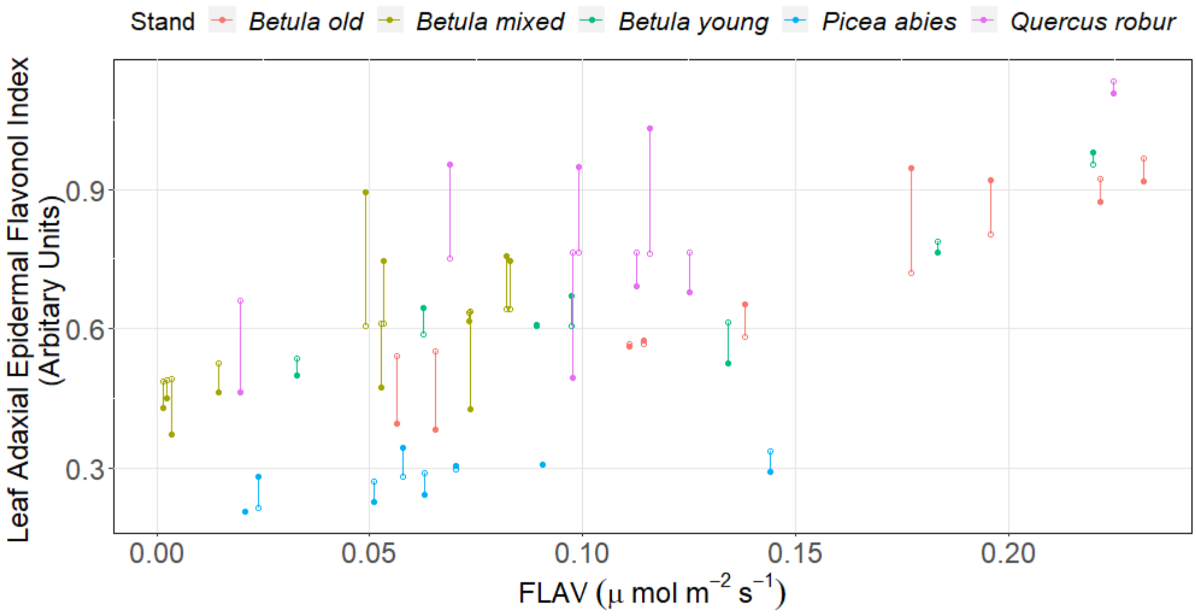

A2 Figure S19. Actual (filled circles) and predicted (empty circles) values from the final model for changes in mean adaxial I_{flav} with effective UV dose calculated according to BSWF for flavonoid accumulation (FLAV) as explanatory variable. Spectral irradiance is measured from understorey *leaf* position.
