# Supplementary material for: Seasonal Patterns in Spectral Irradiance and Leaf UV-A Absorbance Under Forest Canopies
Source: Front Plant Sci. 2020 Feb 18;10:1762. doi: 10.3389/fpls.2019.01762 (PMC7040076; doi:10.3389/fpls.2019.01762)
Supplement: Supplementary file 3 [file DataSheet_3.pdf]

Supplementary Material Appendix 3: Estimation of stand characteristics, canopy phenology, plant area index (PAI) and sunfleck duration

MATERIALS AND METHODS

Surveys of stand characteristics around the measurement points

A circular plot was defined (radius 3.99 m) around each measurement point. Each plot was surveyed for basal area of trees (with a relascope), tree and seedling density, and for the DBH of the 3-4 nearest trees. These measurements were intended to characterise the environment in the immediate vicinity of the measurement points rather than the whole stand.

Estimation of canopy phenology, PAI and sunfleck duration

Canopy tree phenology was surveyed from 3-4 trees adjacent to every measurement point at three different canopy heights (lowest to highest branches) five times during the spring 2015. The plant area index, PAI (m^2 m^{-2}), at the measurement points was calculated from hemispherical photographs taken with Sigma 4.5 mm f2.8 EX DC HSM circular fisheye lens (Sigma Corporation of America, USA) combined with Nikon D7100 (Nikon corporation, Japan) camera body with CMOS 24 MP image sensor in RAW format. Photographs were taken at the same height (40 cm) from the ground as irradiance measurements, levelled on a tripod. Sampling was done during overcast weather to maximise sky homogeneity and contrast between the canopy and sky. A minimum of three pictures with same aperture f 20/22 and ISO 200, but with different exposure time were acquired from each measurement point per DOY. Exposure time was determined manually through inspection with the shortest exposure maximising potential gaps, and the longest exposure excluding any overexposure at the canopy top to obtain the correct range of PAI. All photos were purposefully underexposed compared to automated exposure setting.

Pre-processing of photos was done according to Macfarlane et al. (2014) with some updated changes by the author of the protocol. This protocol produces a gamma corrected and contrast-stretched blue channel 8-bit jpeg photos from the RAW files in order to reduce the variation related to different exposure time. To further reduce unwanted variation related to different exposure times and inconsistent performance of the automated binarisation algorithm (Nobis & Hunziker, 2005), especially with heterogeneous sky conditions, the binarisation of the pre-processed images was also made by using the Floyd-Steinberg dithering option in InfranView (version 4.44, Irfan Skiljan, Wiener Neustadt, Austria) and by standard InfanView binarisation algorithm. All photos were then analysed with Hemisfer 2.16 (Patric Schleppi, Swiss Federal Institute for Forest, Snow and Landscape Research WSL) applying the automatic threshold algorithm (Nobis & Hunziker, 2005) to estimate PAI. Although data was acquired with all equations and corrections, in the analysis only Miller et al. (1967) was used for solving the gap fraction inversion model and foliage clumping was taken into account according to Chen & Cihlar (1995), combined with the non-linearity and slope correction method (Schleppi, 2007). Sunfleck duration on the measurement days was calculated with Gap Light Analyser (GLA, version 2.0, Simon Fraser University, BC, Canada) using the same thresholds determined in Hemisfer software. Finally, the mean PAI and sunfleck duration were calculated for each measurement point and time (DOY) from photos with different exposures. The differences with mean PAI or sunfleck duration

calculated with different binarisation methods were investigated by Pearson's correlations and using ANOVA separately for each DOY.

RESULTS

Mean PAI and sunfleck duration calculated for photos binarised with Floyd-Steinberg dithering and InfranView standard binarisation method were highly correlated, and the grayscale photos binarised via the Nobis & Hunziker (2005) automated binarisation algorithm had slightly weaker relationship with other binarisation methods (A3 Table S1). This was likely due to visually obvious difficulties in performance of the automated algorithm, mostly stemming from intentionally different exposure times of the photographs and occasional cloud cover heterogeneity. PAI estimate calculated with software Hemisfer and two different PAI estimates calculated with software GLA with four and five rings were also highly correlated (r = 0.98/0.99 respectively, $n = 1193$, $p < 0.001$), indicating similarity in estimates between the two software programs and reassuring us of the reliability of sunfleck duration estimates calculated with GLA. Furthermore, surveyed canopy phenology was also highly correlated both with mean PAI and sunfleck duration as expected (A3 Table S1).

Both PAI and sunfleck duration were significantly different among the stands when tested separately for each measurement time (A3 Table S2) and these results did not change when *Picea abies* stand was excluded from the analyses (data not shown). As expected, PAI was stable in the *Picea abies* stand with mean PAI ranging between 3.8-4.0 through season when calculated from pictures binarised with standard binarisation algorithm (A3 Table S2). In the *Betula* old and *Quercus robur* stands the increase in PAI and decrease in sunfleck duration was found later than those in *Betula* mixed and *Betula* young stands (A3 Table S2).

The strongest relationships between mean PAI or sunfleck duration and spectral irradiance (UV-B, UV-A, PAR and effective UV doses calculated according to biological spectral weighting functions: FLAV (Ibdah et al., 2002), PG (Flint & Caldwell, 2003), GEN (G) (Green et al., 1974) action spectrum) were clearly those in understorey shade (A3 Table S1). The relationship between PAR and both mean PAI or sunfleck duration in all understorey positions was weaker than that between UV-B or UV-A irradiance and PAI or sunfleck duration (A3 Table S1). Effective UV doses calculated for different plant functions also had strong relationships with mean PAI and sunfleck duration, especially FLAV and PG spectral weighting functions (A3 Table S1). Mean flavonol index calculated for each measurement point was highly correlated with respective mean PAI and sunfleck duration, except in *Picea abies* stand where no correlation was found (A3 Table S3). Interestingly, this relationship also seemed slightly weaker compared to other stands in *Quercus robur* stand (A3 Table S3). In general, surveyed canopy phenology estimates had a strong relationship with mean flavonol index as well (r = -0.84, $n = 80$, $p < 0.001$).

A3 Table S1. The relationship between Plant Area Index (PAI, m^2 m^{-2}) or sunfleck duration in minutes (sf min) calculated with different methods, and different spectral regions or tree phenology. Spectral photon irradiance (µmol m^{-2} s^{-1}) was measured in understorey sunflecks, shade, *leaf* position, where *leaf* position refers to radiation that is transmitted through the canopy of leaves. Effective UV doses (µmol m^{-2} s^{-1}) were calculated according to biological spectral weighting function for flavonoid accumulation (FLAV action spectrum, Ibdah et al. 2002), for plant growth (PG action spectrum, Flint & Caldwell 2003) and for mathematical formulation for generilised plant action spectrum (GEN(G), Green et al. 1974).

Understorey position	Calculation	Binarisation method	FS†		Automated binarisation		PAR (PPFD)		UV-B		UV-A		FLAV		PG		GEN(G)		Tree phenology	
			r	Sig.	*r*	Sig.	*r*	Sig.	*r*	Sig.	*r*	Sig.	*r*	Sig.	*r*	Sig.	*r*	Sig.	*r*	Sig.
Sunfleck	PAI	Standard	0.998	***	0.90	***	-0.43	***	-0.50	***	-0.54	***	-0.53	***	-0.55	***	-0.34	**	0.84	***
	PAI	FS†			0.89	***	-0.44	**	-0.51	***	-0.55	***	-0.54	***	-0.56	***	-0.35	**	0.84	***
	PAI	Automated					-0.30	*	-0.38	**	-0.42	***	-0.42	***	-0.43	***	-0.24	*	0.80	***
Leaf	PAI	Standard	0.995	***	0.88	***	-0.40	**	-0.58	***	-0.49	***	-0.58	***	-0.52	***	-0.58	***	0.62	***
	PAI	FS†			0.85	***	-0.42	**	-0.61	***	-0.51	***	-0.60	***	-0.54	***	-0.60	***	0.64	***
	PAI	Automated					-0.29	*	-0.45	**	-0.36	*	-0.45	**	-0.39	**	-0.47	***	0.46	**
Shade	PAI	Standard	0.998	***	0.91	***	-0.81	***	-0.90	***	-0.90	***	-0.92	***	-0.91	***	-0.85	***	0.84	***
	PAI	FS†			0.90	***	-0.82	***	-0.91	***	-0.91	***	-0.92	***	-0.92	***	-0.85	***	0.84	***
	PAI	Automated					-0.72	***	-0.83	***	-0.83	***	-0.84	***	-0.84	***	-0.79	***	0.80	***
Sunfleck	Sf min	Standard	0.998	***	0.93	***	0.49	***	0.57	***	0.61	***	0.61	***	0.61	***	0.41	***	-0.85	***
	Sf min	FS†			0.91	***	0.50	***	0.58	***	0.61	***	0.62	***	0.62	***	0.42	***	-0.82	***
	Sf min	Automated					0.42	***	0.50	***	0.53	***	0.53	***	0.54	***	0.34	**	-0.87	***
Leaf	Sf min	Standard	0.997	***	0.96	***	0.47	***	0.61	***	0.56	***	0.62	***	0.58	***	0.58	***	-0.57	***
	Sf min	FS†			0.95	***	0.49	***	0.63	***	0.57	***	0.63	***	0.59	***	0.60	***	-0.58	***
	Sf min	Automated					0.47	***	0.58	***	0.54	***	0.59	***	0.56	***	0.56	***	-0.52	***
Shade	Sf min	Standard	0.998	***	0.93	***	0.77	***	0.92	***	0.90	***	0.93	***	0.91	***	0.88	***	-0.85	***
	Sf min	FS†			0.91	***	0.76	***	0.91	***	0.88	***	0.92	***	0.90	***	0.88	***	-0.82	***
	Sf min	Automated					0.74	***	0.87	***	0.86	***	0.88	***	0.87	***	0.83	***	-0.87	***

Sunfleck *n*=72, leaf *n*=49 and shade *n*=73. † Floyd-Steinberg dithering method. Significance levels: * <0.05, **≤0.01, ***≤0.001

A3 Table S2. Mean ± (SE) Plant Area Index (PAI, m^2 m^{-2}) and sunfleck duration in minutes (sf min) in five forest stands during spring and summer 2015, and differences between the stands.

DOY	Calculation	Binarisation method	*Betula* old	*Betula* mixed	*Betula* young	*Picea abies*	*Quercus robur*	All mean ± (SE)	F (df 4,15)	Sig.
115	PAI	Standard	1.10±0.13	1.32±0.06	1.17±0.04	3.88±0.15	1.11±0.09	1.72±0.25	146.3	***
	PAI	FS†	1.10±0.10	1.31±0.05	1.12±0.03	3.75±0.13	1.09±0.06	1.67±0.24	198.7	***
	PAI	Automated	2.06±1.29	1.98±0.40	1.09±0.05	5.06±0.26	1.12±0.12	2.26±0.42	7.0	**
125	PAI	Standard	0.80±0.02	1.14±0.04	1.09±0.06	3.66±0.17	1.12±0.04	1.56±0.24	202.0	***
	PAI	FS†	0.83±0.03	1.13±0.04	1.06±0.04	3.63±0.15	1.12±0.05	1.55±0.24	248.3	***
	PAI	Automated	0.70±0.03	1.20±0.06	1.00±0.06	4.71±0.21	1.02±0.03	1.73±0.35	261.4	***
142	PAI	Standard	1.36±0.02	2.22±0.06	2.60±0.08	3.97±0.14	1.17±0.03	2.26±0.23	205.3	***
	PAI	FS†	1.36±0.02	2.15±0.05	2.40±0.05	3.95±0.10	1.16±0.02	2.20±0.23	379.7	***
	PAI	Automated	2.16±0.08	3.46±0.21	4.51±0.13	5.45±0.09	1.24±0.05	3.36±0.35	186.3	***
156	PAI	Standard	2.38±0.07	3.50±0.05	3.61±0.17	4.00±0.22	2.75±0.11	3.25±0.15	22.2	***
	PAI	FS†	2.30±0.07	3.37±0.04	3.43±0.13	3.95±0.17	2.58±0.09	3.13±0.15	35.8	***
	PAI	Automated	3.76±0.37	4.68±0.09	4.83±0.17	5.22±0.35	4.46±0.57	4.59±0.18	2.4	NS
202	PAI	Standard	2.85±0.14	3.93±0.06	3.82±0.09	3.84±0.18	3.34±0.08	3.56±0.10	14.7	***
	PAI	FS†	2.82±0.13	3.81±0.06	3.66±0.06	3.81±0.16	3.22±0.07	3.47±0.10	17.9	***
	PAI	Automated	3.65±0.24	4.78±0.08	4.83±0.13	4.77±0.17	4.35±0.15	4.47±0.12	9.6	***
115	Sf min	Standard	332.36±16.18	291.40±11.08	284.73±8.99	29.80±3.33	344.52±22.22	256.56±27.11	86.2	***
	Sf min	FS†	329.18±15.30	285.03±10.61	272.23±11.08	34.10±3.30	335.56±16.20	251.22±25.99	104.2	***
	Sf min	Automated	304.16±168.19	220.25±94.51	304.30±20.09	14.34±5.24	348.72±56.58	238.36±146.54	8.7	***
125	Sf min	Standard	364.64±9.16	315.05±13.83	344.14±4.77	51.31±10.65	396.88±19.84	294.40±28.99	120.2	***
	Sf min	FS†	354.96±10.30	307.11±15.00	340.76±3.61	58.38±10.83	393.91±23.02	291.03±28.01	90.2	***
	Sf min	Automated	398.46±9.34	304.36±17.17	365.15±5.78	27.50±6.60	424.31±12.68	303.96±33.32	208.9	***
142	Sf min	Standard	290.82±14.52	148.12±21.16	158.04±7.05	43.67±6.46	435.95±10.69	215.32±31.49	132.5	***
	Sf min	FS†	298.32±14.48	158.03±22.72	175.87±5.12	50.17±6.78	428.37±9.31	222.15±30.23	118.9	***
	Sf min	Automated	155.02±27.95	78.73±37.56	60.76±10.51	22.75±8.12	426.12±30.81	148.68±150.77	159.0	***
156	Sf min	Standard	173.05±12.09	94.86±14.43	95.23±12.32	37.97±7.90	157.00±18.38	111.62±12.37	16.3	***
	Sf min	FS†	187.50±12.20	104.21±14.19	105.29±10.51	44.78±8.45	175.60±18.93	123.48±13.13	19.2	***
	Sf min	Automated	98.85±24.46	56.54±15.42	43.82±16.30	20.34±11.32	80.25±42.43	59.96±35.66	6.2	**
202	Sf min	Standard	71.50±10.97	39.44±2.54	78.19±5.69	48.51±8.18	82.36±10.47	64.00±5.07	5.4	**
	Sf min	FS†	80.73±10.62	46.43±3.43	88.18±6.57	53.60±8.11	94.83±9.46	72.76±5.45	7.2	**
	Sf min	Automated	37.89±15.77	23.06±1.92	40.00±6.29	31.06±6.93	45.14±13.36	35.43±13.61	1.9	NS

Starting values for analyses are means extracted from 3-5 photos analysed for PAI and sunfleck duration i.e. approximately 16 photos in total for each stand per DOY. † Floyd-Steinberg dithering method. Significance levels: * <0.05, **≤0.01, ***≤0.001

A3 Table S3. Relationship between mean flavonol index (I_{flav}) and mean Plant Area Index (PAI, $m^2\ m^{-2}$) or sunfleck duration in minutes (sf min) with standard binarisation method.

Stand	Calculation	*r*	Sig.
Betula old	PAI	-0.94	***
Betula mixed	PAI	-0.94	***
Betula young	PAI	-0.84	***
Picea abies	PAI	-0.22	NS
Quercus robur	PAI	-0.79	***
All	PAI	-0.90	***
Betula old	Sf min	0.94	***
Betula mixed	Sf min	0.90	***
Betula young	Sf min	0.87	***
Picea abies	Sf min	-0.08	NS
Quercus robur	Sf min	0.77	***
All	Sf min	0.89	***

Significance levels: * <0.05, **≤0.01, ***≤0.001

REFERENCES

Chen, J. M., & Cihlar, J. (1995). Plant canopy gap-size analysis theory for improving optical measurements of leaf-area index. *Applied Optics, 34*(27), 6211-6222. doi:10.1364/AO.34.006211

Flint, S. D., & Caldwell, M. M. (2003). Field testing of UV biological spectral weighting functions for higher plants. *Physiologia Plantarum, 117*(1), 145-153. doi:10.1034/j.1399-3054.2003.1170118.x

Green, A. E. S., Sawada, T., & Shettle, E. P. (1974). The middle ultraviolet reaching the ground. *Photochemistry and Photobiology, 19*(4), 251-259. doi:10.1111/j.1751-1097.1974.tb06508.x

Ibdah, M., Krins, A., Seidlitz, H. K., Heller, W., Strack, D., & Vogt, T. (2002). Spectral dependence of flavonol and betacyanin accumulation in mesembryanthemum crystallinum under enhanced ultraviolet radiation. *Plant, Cell & Environment, 25*(9), 1145-1154. doi:10.1046/j.1365-3040.2002.00895.x

Macfarlane, C., Ryu, Y., Ogden, G. N., & Sonnentag, O. (2014). Digital canopy photography: Exposed and in the raw. *Agricultural and Forest Meteorology, 197*, 244-253. doi:10.1016/j.agrformet.2014.05.014

Miller, J. B. (1967). A formula for average foliage density. *Australian Journal of Botany, 15*(1), 141-144. doi:10.1071/BT9670141

Nobis, M., & Hunziker, U. (2005). Automatic thresholding for hemispherical canopy-photographs based on edge detection. *Agricultural and Forest Meteorology, 128*(3), 243-250. doi:10.1016/j.agrformet.2004.10.002

Schleppi, P., Conedera, M., Sedivy, I., & Thimonier, A. (2007). Correcting non-linearity and slope effects in the estimation of the leaf area index of forests from hemispherical photographs. *Agricultural and Forest Meteorology, 144*(3), 236-242. doi:10.1016/j.agrformet.2007.02.004
